# Structural characterization of CAS SH3 domain selectivity and regulation reveals new CAS interaction partners

**DOI:** 10.1038/s41598-017-08303-4

**Published:** 2017-08-14

**Authors:** Jakub Gemperle, Rozálie Hexnerová, Martin Lepšík, Petr Tesina, Michal Dibus, Marian Novotný, Jan Brábek, Václav Veverka, Daniel Rosel

**Affiliations:** 1 Department of Cell Biology, Faculty of Science, Charles University, Vinicna 7, Prague, Czech Republic; 2 Institute of Organic Chemistry and Biochemistry of the Czech Academy of Sciences, Flemingovo nam. 2, Prague, Czech Republic

## Abstract

CAS is a docking protein downstream of the proto-oncogene Src with a role in invasion and metastasis of cancer cells. The CAS SH3 domain is indispensable for CAS-mediated signaling, but structural aspects of CAS SH3 ligand binding and regulation are not well understood. Here, we identified the consensus CAS SH3 binding motif and structurally characterized the CAS SH3 domain in complex with ligand. We revealed the requirement for an uncommon centrally localized lysine residue at position +2 of CAS SH3 ligands and two rather dissimilar optional anchoring residues, leucine and arginine, at position +5. We further expanded the knowledge of CAS SH3 ligand binding regulation by manipulating tyrosine 12 phosphorylation and confirmed the negative role of this phosphorylation on CAS SH3 ligand binding. Finally, by exploiting the newly identified binding requirements of the CAS SH3 domain, we predicted and experimentally verified two novel CAS SH3 binding partners, DOK7 and GLIS2.

## Introduction

Mammalian Crk-associated substrate (CAS), a major substrate of Src kinase, plays an important role in oncogenic transformation mediated by the v-*crk* and v-*src* oncogenes (reviewed by ref. 1). Tyrosine phosphorylated CAS is enriched in focal adhesions and podosome rosettes^2, 3^. In Src-transformed cells, CAS expression is required to promote cell invasiveness and lung metastasis^4^. Furthermore, increased levels of the human ortholog of CAS, BCAR1, are associated with exacerbated prognosis in breast cancer patients^5^.

CAS serves as an adaptor protein in multiprotein signaling complexes. CAS consists of an N-terminal Src Homology 3 (SH3) domain, a large central substrate domain (SD) formed by 15 repeats of the YxxP motif followed by a serine-rich domain, and a C-terminal part composed of binding sites for the SH2 and SH3 domains of Src (YDYVHL and RPLPSPP, respectively) and a CAS-family C-terminal homology domain (reviewed in ref. 1). The SD domain contains multiple tyrosine phosphorylation sites and is essential for the invasive and metastatic properties of CAS^4^. The phosphorylation of tyrosines in SD by Src family kinases enables CAS interactions with the Crk and Nck adapters^6, 7^. The extent of this phosphorylation is regulated by the CAS SH3 domain, which mediates the interaction of CAS with polyproline motifs of various kinases (FAK, PYK2/RAFTK, FRNK), phosphatases (PTP1B, PTP-PEST), and other proteins (C3G, CMS, CIZ and Vinculin)^8–15^. The CAS SH3 domain is indeed important for tyrosine phosphorylation of the SD, as experiments with truncated CAS lacking the SH3 domain showed a decreased level of SD tyrosine phosphorylation^4, 16^. The tyrosine phosphorylation of SD can be enhanced by mechanical extension^17^, and this finding suggested a mechanism allowing CAS to function as a primary mechanosensor^18^. Notably, the critical role of CAS SH3 in mechanosensing and SD phosphorylation-mediated mechanotransduction was described recently^19^. Finally, signaling mediated by the CAS SH3 domain may be regulated by changing the affinity of the SH3 domain to its ligands though phosphorylation of Tyr12 within the SH3 domain^20, 21^.

The SH3 domain is a small protein interaction module of approximately 60 amino acids. Its conserved β-sandwich architecture is composed of five antiparallel β strands connected by three loops (n-Src loop, RT loop, and Distal loop) and a short 3_10_ helix. The minimal consensus sequence for SH3 domain ligands is represented by the PxxP binding motif, which interacts with two xP dipeptide binding pockets formed on the SH3 surface. The SH3 domain binding specificity is further defined by a negatively charged third cleft called the specificity zone/pocket, which binds a positively charged residue that is usually present at the N- or C-terminus of the pseudo-symmetrical PxxP motif and drives ligands on the SH3 domain either in an N-to-C (Class II ligands) or C-to-N (Class I ligands) orientation^22^. Flanking residues, also known as short distance elements (SDEs), that bind to less conserved portions of the SH3 surface can additionally increase the binding specificity and affinity^23^.

**Figure 1:**
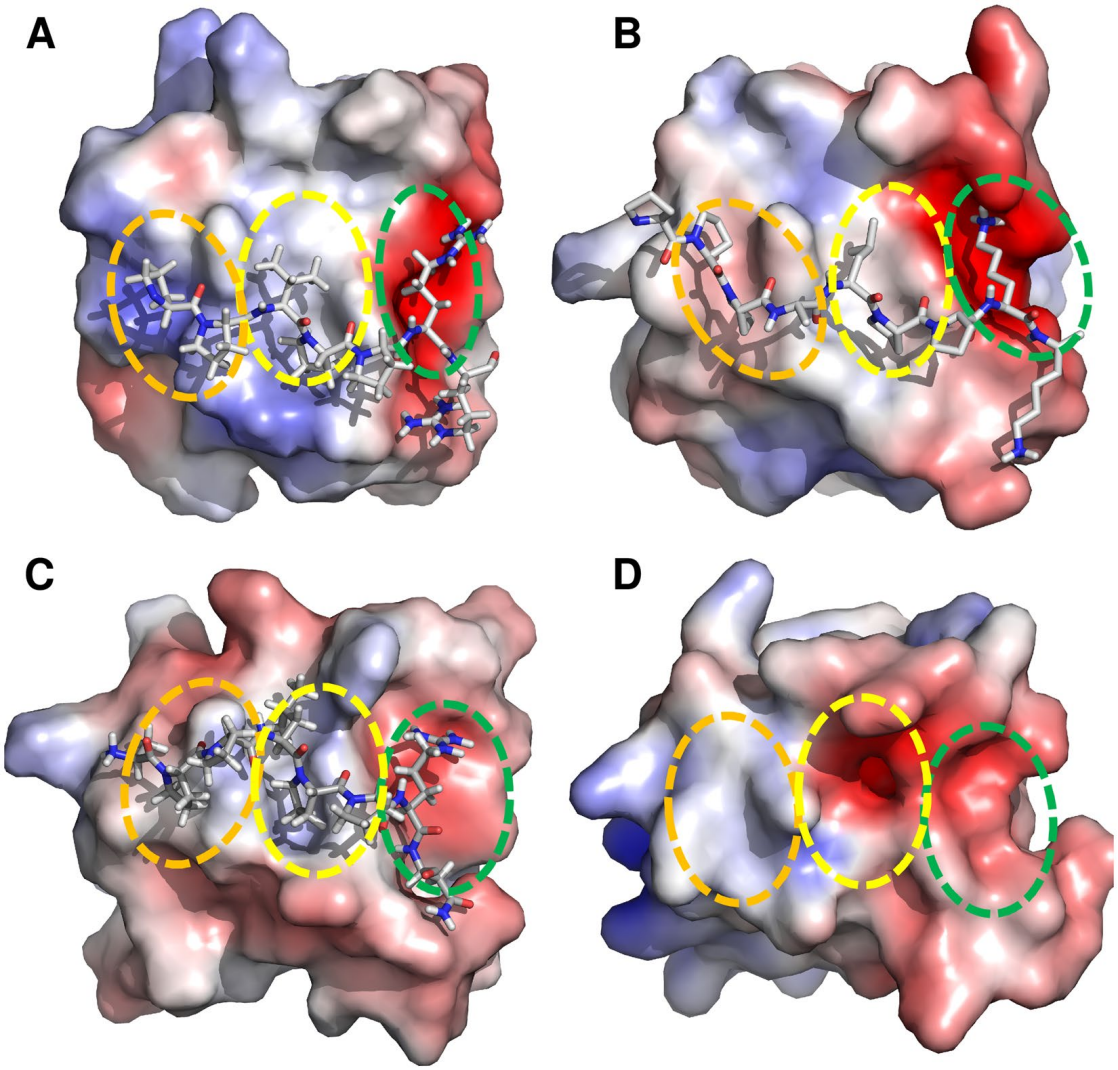
SH3 domains of proteins involved in the CAS signaling circuit. (**A**) (Grb2, PDB code 1AZE), (**B**) (Crk, PDB code 1CKA), (**C**) (Src, PDB code 1QWE), (**D**) (CAS, PDB code 1WYX). The first and second xP dipeptide-binding pockets are highlighted with orange and yellow circles, respectively. The third pocket/zone is highlighted with a green circle. SH3 domains are shown in electrostatic potential surface representation (APBS colored in the range from −5 to +5).

SH3 domains generally bind to their targets with a relatively low affinity (K_d_ = 5–100 µM) and moderate specificity^24^. Comparison of the CAS SH3 domain with SH3 domains of proteins involved in the same signaling circuit, such as the well-defined SH3 domains of Crk, Src, and Grb2, reveals a difference in the hydrophobicity of the second xP dipeptide-binding pocket (Fig. 1). While the second binding pocket in all these SH3 domains is mostly hydrophobic, the CAS SH3 domain also includes a negatively charged Glu17 residue. The presence of this unusual negatively charged cleft could contribute to the CAS SH3 domain specificity, perhaps recognizing unusual polyproline rich sequences. However, a systematic screen for CAS SH3 domain ligand preferences has not yet been performed, and despite the known structure of the CAS SH3 domain, the structural determinants of its regulation are not well understood.

By using a multidisciplinary approach combining molecular and structural biology, biophysics, bioinformatics, and computational modeling, we determined the CAS SH3 binding motif and obtained solution structures for the CAS SH3 domain in complex with its natural peptide ligands^8, 10^. These structural data, together with characterization of thermodynamic parameters by isothermal titration calorimetry (ITC) and modeling, allowed us to investigate the affinity contributions from the indispensable centrally located lysine and conserved (arginine) and unusual (leucine) anchoring amino acids in the binding peptides. Based on the structural data, we predicted the effect of Tyr12 phosphorylation. Furthermore, based on the determination of the high-affinity consensus motif, we identified novel CAS SH3 binding partners, DOK7 and GLIS2, and confirmed their interactions in living cells.

## Results

### The CAS SH3 domain recognizes an unconventional class II SH3 binding motif

**Figure 2:**
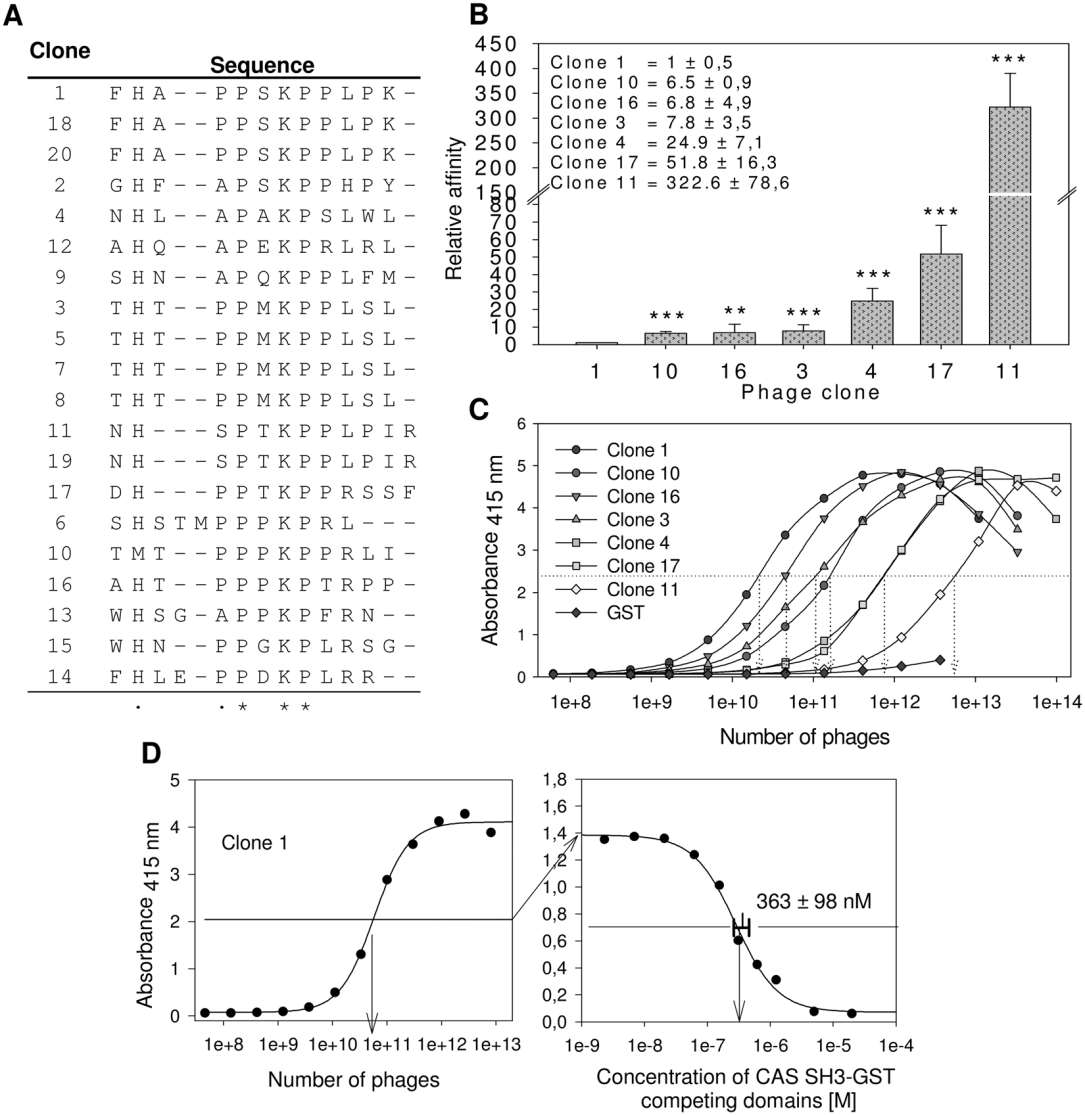
Screening of the CAS SH3 domain binding motif using phage display. (**A**) Sequences of 20 aligned clones. (**B**) ELISA quantification of the relative affinities of seven phage-displayed peptides towards the GST-fused CAS SH3 domain. Relative affinities were calculated based on the binding affinity of clone 1 (the tightest binder). The data are expressed as average ± standard deviation from five independent experiments. Statistical significance (**p < 0.01, ***p < 0.001) was determined on log-transformed data by one-way repeated-measures ANOVA followed by Tukey’s post-hoc test. (**C**) Representative curves of relative affinities of seven phage-displayed peptides towards the GST-fused CAS SH3 domain. GST alone with clone 1 was used as a negative control. **D** Representative graph for the EC_50_ value (363 +/− 98 nM) for clone 1 binding obtained from seven independent experiments.

The indispensable role of the CAS SH3 domain for CAS/BCAR1-mediated signaling is well-documented. However, to date the CAS SH3 domain-binding motif has not been precisely defined. To identify the binding motif, we performed phage display with a library composed of 10^12^ M13 phages carrying 12-amino-acid degenerate oligopeptides. For the peptide binding selection, GST-fused CAS SH3 was immobilized on GSH-agarose beads and used for biopanning. After four rounds of panning, 20 phages were isolated and sequenced. This procedure yielded 14 unique sequences encoding CAS SH3 binding peptides (Fig. 2A). The interaction specificity was confirmed by phage ELISA, and qualitative determination of relative binding affinities was performed for seven clones (Fig. 2B,C). We determined an EC_50_ value of 363 nM for clone 1 (FHAPPSKPPLPK), which showed the highest relative affinity (Fig. 2D). This corresponds to an almost 6-fold higher affinity than that previously reported for the interaction of the CAS SH3 domain with FAK-derived peptide^25^, suggesting that we discovered a high-affinity ligand for the CAS SH3 domain.

**Figure 3:**
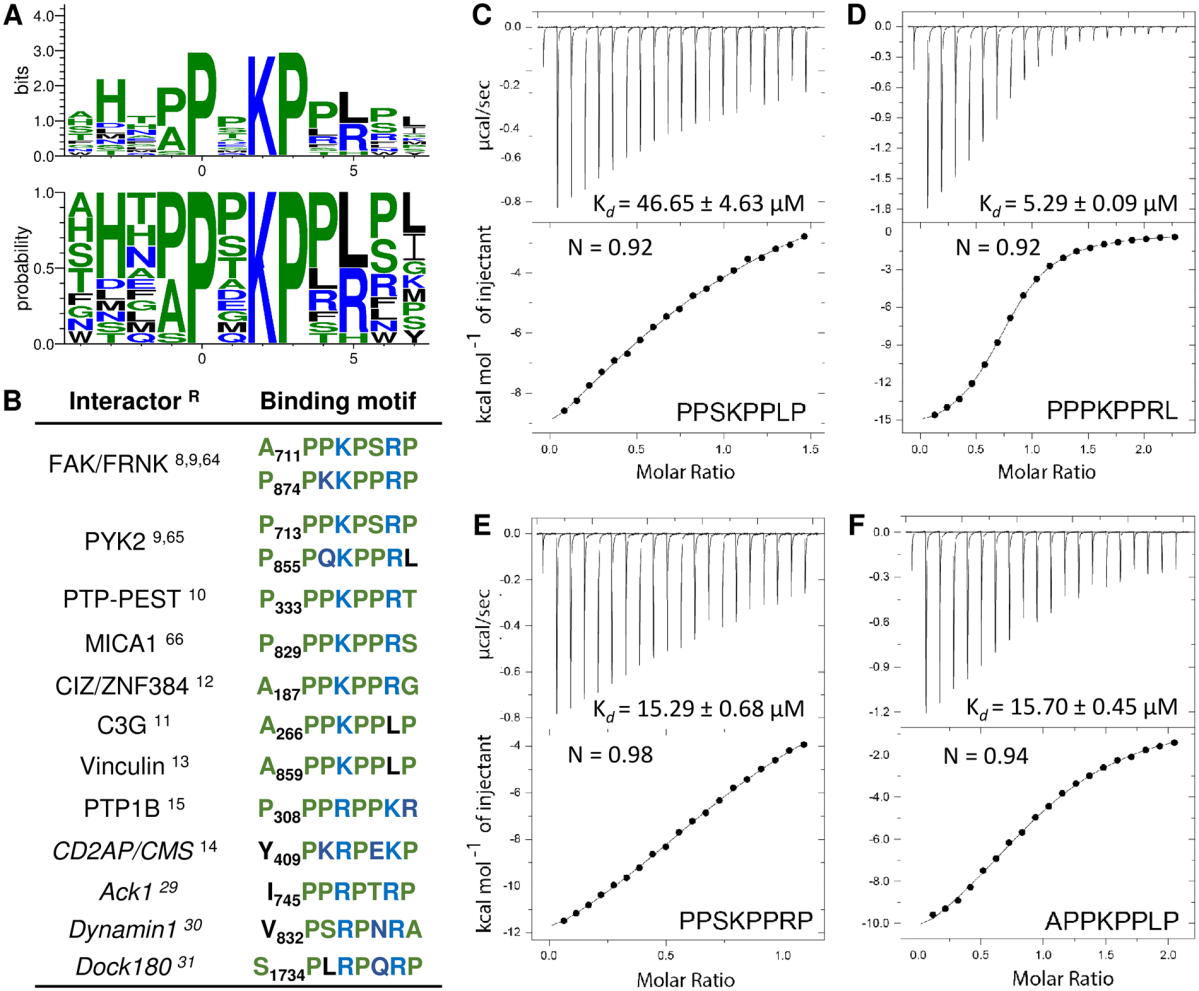
(**A**) The CAS SH3 binding motif based on the 14 unique sequences obtained from phage display. The x-axis shows the residue position relative to proline (position 0)^64–66^. (**B**) The CAS SH3 domain binding interaction partners with their respective binding motifs. Interactors with small differences in binding motif are in italics. References (^R^) are superscripted. (**C**–**E**) Isothermal titration calorimetry (ITC) data obtained for the interaction of CAS SH3 with four synthetic peptides.

Based on the phage display and phage ELISA results, we determined the CAS SH3 domain high-affinity binding motif as an eight-amino-acid sequence: (**A**/**P**)_−1_ **P**_**0**_ × _1_ **K**_**2**_ **P**_**3**_ × _4_(**L**/**R**)_5_Z_6_ (Fig. 3A, Z stands for L/P/S/T). This motif is a class II ligand due to the N-to-C orientation in which position +2 is usually occupied by a hydrophobic residue. Surprisingly, we observed a clear preference for lysine at this central position in all the clones. Alanine or proline are tolerated at position −1, while serine (clone 11) showed a clear negative effect on CAS SH3 binding. We then compared this binding motif to sequences of the 12 known CAS SH3-interacting partners (Fig. 3B). Most of them are in good agreement with the identified motif, with the exception of an arginine to leucine substitution at position +5 (arginine found in 10 cases, leucine only in two). Clearly, there is a strong preference for a positively charged residue.

To analyze amino acid variations at positions −1 and +5 in the binding motif in more detail, we synthesized four octapeptides and determined their thermodynamic parameters of binding using ITC. Two of the peptides corresponded to the core binding motif of two clones with the highest affinities (clones 1 and 10, peptides PPSKPPLP and PPPKPPRL, respectively), one peptide corresponding to clone 1 but with the arginine to leucine substitution at position +5 (PPSKPPRP) and one corresponding to the binding site of CAS on Vinculin (APPKPPLP), which is closely related to both clone 1 and 10 peptides with alanine at position −1 (Fig. 3C–F). All the tested peptides formed equimolar complexes with CAS SH3. However, the obtained dissociation constants (K_*d*_) revealed significant differences in their affinities towards CAS SH3. The PPPKPPRL peptide showed the highest affinity of all tested peptides (K_*d* = _5.29 ± 0.09 µM). The APPKPPLP and PPSKPPRP peptides exhibited lower but comparable affinities (K_*d* = _15.70 ± 0.45 µM and K_*d* = _15.29 ± 0.68 µM, respectively), while the PPSKPPLP peptide was identified as the weakest CAS SH3 ligand of all tested peptides (K_*d* = _46.65 ± 4.63 µM). Based on these results, we conclude that arginine at position +5 is more favorable for CAS SH3 binding than leucine at the same position, whereas the affinity contribution of alanine and proline at position −1 is indistinguishable.

### Structure of the CAS SH3 domain in complex with its physiological ligands provides insight into the binding interface

With the exceptions of C3G^11^ and the recently identified Vinculin^13^, no other binding partner of CAS harbouring leucine at position +5 has been identified to date. Our results, however, suggest that leucine is not only permitted at this position but also contributes considerably to the binding (see Fig. 2A–C). Moreover, despite the known X-ray structure of the free CAS SH3 domain^25^, the structural aspects of CAS SH3 binding domain regulation are not well understood. We thus sought to analyze the structural basis of the CAS SH3 domain binding to its physiological ligands.

We prepared^15^N/^13^C-labeled CAS SH3 domain and determined its solution structure. The complete resonance assignments and high quality 3D^15^N- and^13^C-edited NOESY spectra were used for structural calculation (Supplementary Table S1). The conformation of the solution structure is highly similar to that obtained by X-ray (PDB code 1WYX; backbone RMSD of 0.52 Å for residues 7–65), but unexpectedly and in contrast to the dimeric X-ray structure, the CAS SH3 domain remained monomeric in solution even at ~1 mM concentration. A more detailed analysis revealed that the crystallographic dimer is stabilized by crystal contacts that stimulate the formation of an additional two-stranded β-sheet at the dimer interface that is unambiguously absent in solution (Supplementary Figure S1).

Next, we attempted to structurally characterize complexes of CAS SH3 with Vinculin and PTP-PEST derived molecules using isotopically labeled recombinant protein and unlabeled synthetic peptides. The Vinculin and PTP-PEST binding peptides were chosen as known physiological ligands representing both variants of the binding sequence, with either arginine (PTP-PEST) or leucine (Vinculin) at position +5. Although we obtained complete resonance assignments for the peptide-bound protein, the polyproline character of the peptides prevented their unambiguous resonance assignment required for the comprehensive structural characterization of the complexes. To determine the structure of the CAS SH3 domain with peptide ligands containing a high number of repetitive prolines using NMR, we designed two chimeras with the peptide sequences located at the C-terminus of the SH3 domain. The CAS-Vinculin and CAS-PTP-PEST chimeras contained the sequences corresponding to residues 854–870 and 327–343, respectively. In both chimeras, the binding peptide was fused to the SH3 domain through a Gly/Ser-rich linker peptide. This allowed us to obtain a high overall percentage of assigned resonances (>98%) and complete assignments within the peptide region (Supplementary Table S1). In addition, the spectra of the peptide-bound protein were highly similar to those obtained for chimeras, except for a subset of signals from the peptide portion of the fusion proteins (Supplementary Figure S2). The minimal differences in signal positions can be attributed to the increased binding affinity between the protein and peptide regions in chimeras relative to the two individual molecules.

**Figure 4:**
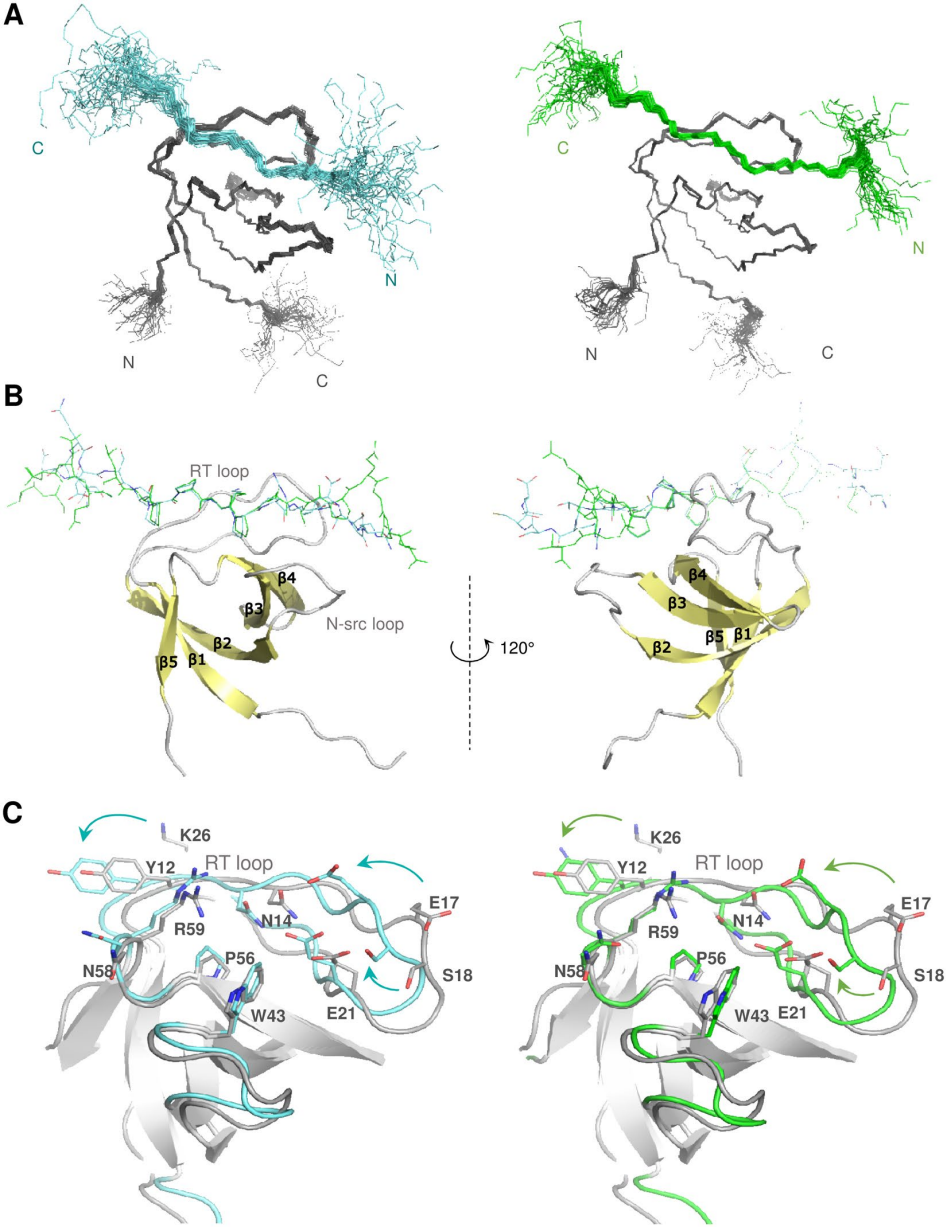
Solution structures of the CAS SH3 domain complexes. (**A**) Set of 40 converged structures of CAS SH3 (dark grey) with PTP-PEST- (cyan, left) and Vinculin-derived peptide (green, right). (**B**) A cartoon representation of the complexes. (**C**) Superimposition of the free CAS SH3 domain (grey) and PTP-PEST- (cyan) and Vinculin-bound (orange) CAS SH3 domains. The β-sheets are in grey.

The NMR analysis of the CAS SH3-peptide ligand chimeras allowed for a detailed characterization of the binding interface between the CAS SH3 domain and the studied peptides (Fig. 4A). As expected, the binding of the peptides does not involve a major conformational rearrangement in the CAS SH3 domain (backbone RMSD of 0.97 Å for Vinculin and 0.98 Å for PTP-PEST, for residues 7–65). Comparison of the free CAS SH3 domain structure and the chimera structures revealed a subtle repositioning of the RT loop, allowing the peptide to maintain contacts with both the RT loop residues (i.e., Asn14, Glu17, Glu21, and Asp20) and residues from the core β-sheet (i.e., Asn58 and Trp43) (Fig. 4B,C). Moreover, the changes in the positions of Asn14, Glu17, and Glu21 and a subtle repositioning of side chain residues situated in the short 3_10_ helix (Val55 and Arg59) were evident from relative chemical shift perturbation analysis of CAS SH3 induced by binding of PTP-PEST or Vinculin peptide (Supplementary Figure S3). The bound structures of the peptides differed in their flexibility. For PTP-PEST, Arg338-Leu343 (positions 5–10) was very flexible, and for Vinculin, the flexible part extended between Glu867 and Val870 (positions 7–10) (Fig. 4A).

**Figure 5:**
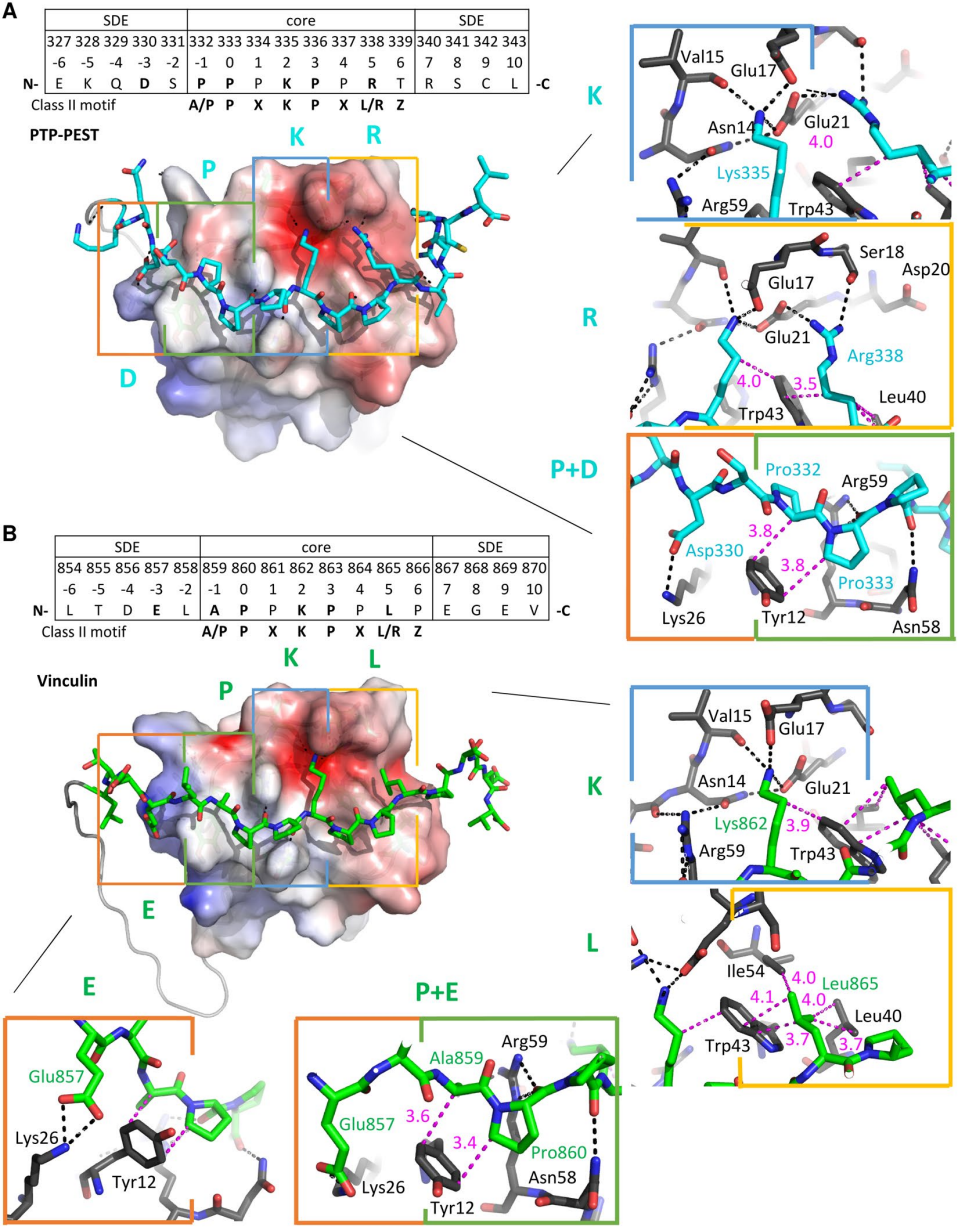
Key interactions between the PTP-PEST- and Vinculin- derived peptides and CAS SH3 domain. (**A**) PTP-PEST- and (**B**) Vinculin-derived peptides are shown in stick representation. The CAS SH3 parts of the complexes are shown by electrostatic potential surface representation (APBS). Four binding pockets are highlighted with rectangles (orange - zero pocket/upstream SDE, green - 1st pocket, blue - 2nd pocket, yellow - 3rd pocket), and the interaction interface is shown on the right in detail (PTP-PEST in cyan, Vinculin in green, CAS SH3 in gray). Capital letters E/D, P, K, L/R represent the key ligand amino acid residue in each CAS SH3 binding pocket. The distances between a pair of residues involved in hydrophobic interaction are shown in pink; hydrogen bonds are represented by black dotted lines.

We utilized the PISA protein interface tool to describe the binding interface of all the NMR generated structural states^26^ (Supplementary Tables S2–7). Both peptides form contacts with the same CAS SH3 residues (specifically Tyr12, Asn14, Glu17, Glu21, Trp43, Pro56, Asn58, and Arg59) mainly *via* lysine (Lys335 in PTP-PEST, Lys862 in Vinculin) as a central anchoring amino acid (K_2_) followed by two proline residues positioned around the Trp43 side chain (Fig. 5). The interaction of CAS SH3 with the PTP-PEST peptide is further stabilized by hydrogen bonds formed between the Arg338 (R_5_) side chain and one of the side chain oxygens of Glu21, Ser18, Glu17, or Asp20 of CAS SH3 (Fig. 5, Supplementary Tables S2–4). In Vinculin peptide, this position is occupied by hydrophobic Leu865 (L_5_) oriented towards Trp43, Ile54, and Leu40 of CAS SH3 (Fig. 5, Supplementary Tables S5–7). The contacts of the peptide ligands with the Tyr12 side chain of SH3 domain are mediated by the main chain Pro332 (P_−1_) and Pro333 (P_0_) in PTP-PEST and by Ala859 (A_−1_) and Pro860 (P_0_) in Vinculin.

### CAS SH3 domain binds to short distance elements

As described previously, the binding specificity and affinity of the ligand can be further increased by binding of the flanking ligand residues (SDEs) to the SH3 domain surface located in the vicinity of the third binding pocket (i.e., C-terminally from the core peptide)^23^. Because our chimeric constructs also included nine amino acids corresponding to the sequence surrounding the core binding sequence on both sides, we were able to assess the potential effect of these flanking sequences on binding. At the C-terminal side of the anchoring Arg338 (R_5_), there were variable H-bond interactions between Arg340 (R_7_) and Gly39, Gln38, or Asp41 of CAS SH3 bound to PTP-PEST (Supplementary Tables S2–4). Interestingly, the N-terminal SDEs formed interactions with the region proximal to the first binding pocket. In PTP-PEST, Asp330 (D_−3_) formed a transient H-bond (with 21–50% occupancy) with Lys26, which alternated with a weak H-bond between Glu327 (E_−6_) and Arg25 (Supplementary Tables S2–4, visualized in Fig. 5). In the case of Vinculin (Supplementary Tables S5−7, visualized in Fig. 5), there was one strong, conserved H-bond (Lys26…Glu857/E_−3_). In addition, Lys26 established irregular weak H-bonds with Thr855 (T_−5_) and Asp856 (D_−4_).

These findings are supported by the results of a sequence alignment of the CAS SH3 domain binding partners. The alignment revealed an enrichment of negatively charged amino acid residues localized N-terminal to the polyproline core, although their exact position is not conserved (Supplementary Figure S4). Similar interactions of negatively charged SDEs with region proximal to the first binding pocket have not been described before; however, a lysine residue corresponding to Lys26 is conserved in 35.6% of human SH3_1 domains within the PFAM database, suggesting that other SH3 domains might utilize a similar mechanism to extend the ligand specificity and binding affinity.

### Binding energy and sequence conservation analysis point to determinants of the CAS SH3 domain complex formation

**Figure 6:**
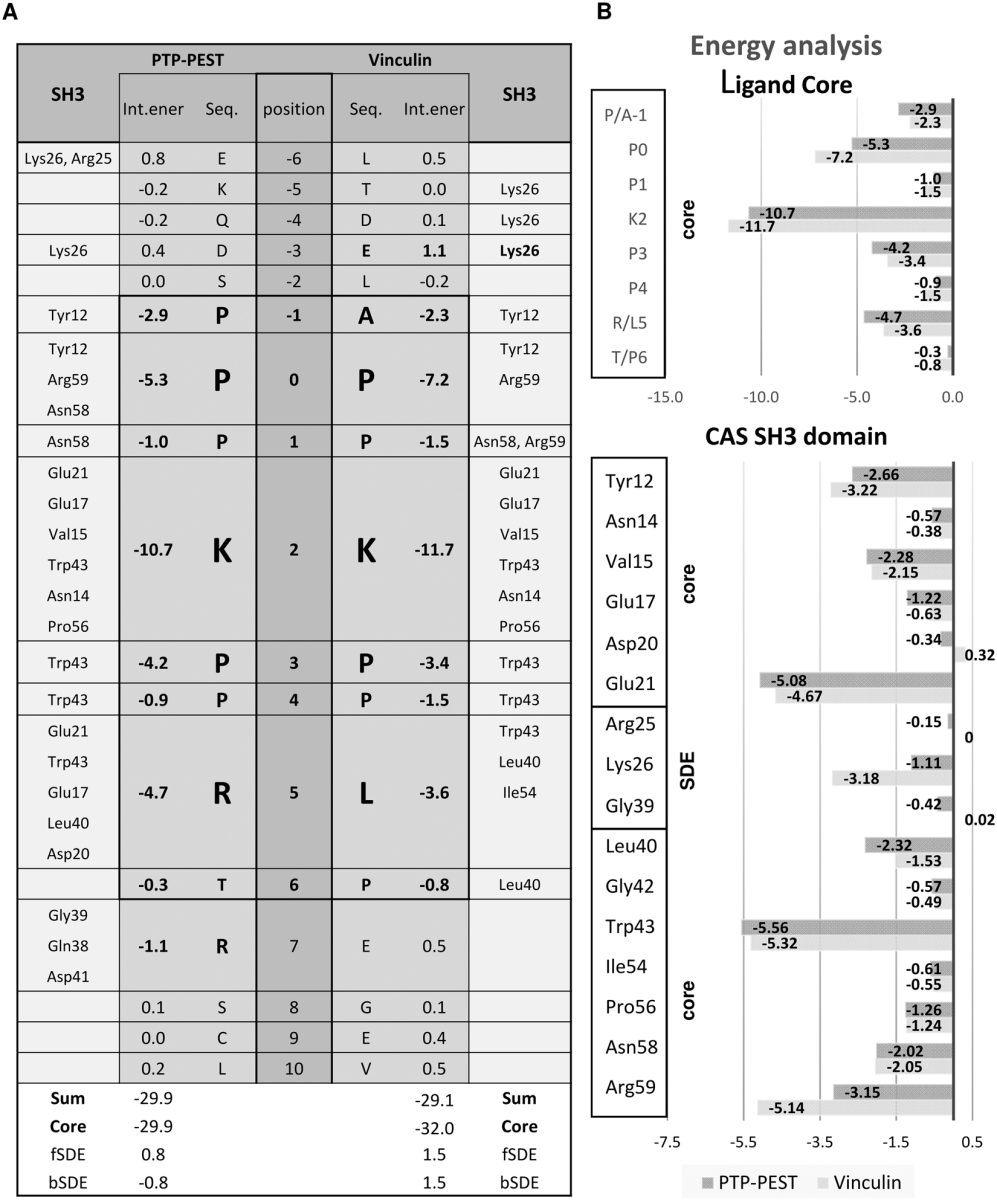
The attractive MM-GBSA energy contributions of individual protein and peptide residues toward binding (in kcal/mol). (**A**) Energy contribution of binding sequences of PTP-PEST or Vinculin to the CAS SH3 domain in fragmented CAS SH3 domain NMR structures. The peptide binding core is highlighted (-1 to 6). (**B**) Upper graph shows the CAS SH3 domain ligand-core binding energy contributions. Lower graph shows the energy contributions of CAS SH3 amino acid residues to binding Vinculin or PTP-PEST. fSDE: N-terminal SDE; bSDE: C-terminal SDE.

To understand the mechanism of the CAS SH3 domain ligand binding in energetic terms, we performed MM-GBSA analysis (see Methods) on all the NMR models. The affinities of the PTP-PEST and Vinculin extended peptides (corresponding to residues 327–343 and 854–870, respectively) towards the CAS SH3 domain were estimated as −57.0 ± 1.6 and −57.8 ± 0.8 kcal.mol^−1^, respectively. This suggests comparable binding affinity, in agreement with the experimentally determined affinities for the PTP-PEST and Vinculin core peptides (332–339 and 859–866) of 5.3 and 15.7 μM, respectively. All energy contributions for PTP-PEST/Vinculin were determined in kcal.mol^−1^ (Fig. 6A,B). The sums of the interaction contributions from the peptide residues (positions −6 to 10) were −29.9/−29.1. Nearly the entire interaction strength comes from the peptide core residues (positions −1 to 6; the energy sums were −29.9/−32.0). The largest contributions come from K_2_ (−10.7/−11.7; Lys335/862), followed by P_0_ (−5.3/−7.2; Pro333/860), R/L_5_ (−4.7/−3.6; Arg338/Leu865), and P_3_ (−4.2/−3.4; Pro336/Pro863). The MM-GBSA analysis of the core peptide binding is consistent with the PISA interaction interface analysis (Supplementary Tables S2–7). In contrast, the MM-GBSA analysis of the contribution of SDE elements to the binding confirmed only the stabilizing role of the C-terminal SDE, specifically Arg340 (R_7_) in PTP-PEST. For the N-terminal SDE, MM-GBSA analysis suggests repulsive rather than attractive forces at position −3. However, it should be noted that the repulsive contribution of Glu857/E_−3_ in Vinculin of 1.1 kcal.mol^−1^ may result from an improper partitioning of interaction energy and that, summing up with the favorable contribution of Lys26 of −3.2 kcal.mol^−1^, this conserved salt bridge contributes −2.0 kcal.mol^−1^ toward binding.

The MM-GBSA energies suggest that strong attractive interactions are derived from the SH3 domain residues Trp43, Glu21, Arg59, and Tyr12 for both PTP-PEST and Vinculin, followed by weaker contributions from Leu40, Val15, Asn58, Pro56, Glu17, and Lys26 (Fig. 6B). These favorable contributions can be linked to individual hydrogen bonds (mediated by Arg59 and Asn58) including salt bridges (Glu21, Glu17, Lys26) as well as nonpolar (dispersion) interactions (Trp43, Tyr12, Leu40, Val15, and Pro56) and are conserved in both PTP-PEST and Vinculin. Glu21 forms a strong salt bridge with K_2_ (Lys335/862), which is in turn locked by a bidentate interaction with the Asn14 side chain (Fig. 5). Arg59 forms conserved bidentate H-bonds *via* its side chain with the backbone of P_0_ (Pro333/860)_._ Asn58 serves as a donor to a less conserved H-bond with P_1_ (Pro334/861) backbone of the ligands. The Glu17 side chain forms a conserved salt bridge with K_2_ (Lys335/862) and a much less conserved salt bridge with the anchoring R_5_ (Arg338) of PTP-PEST. The major contributor toward nonpolar stabilization is Trp43, which establishes many hydrophobic contacts with the nonpolar parts of the side chains of K_2_ (Lys335/862), P_3_ (Pro336/Pro863), and R/L_5_ (Arg338/Leu865). Furthermore, the phenol ring of Tyr12 packs against the side chains of A/P_−1_ (Pro332/Ala859) and P_0_ (Pro333/860). Pro56 is involved in dispersion contacts with the nonpolar parts of the side chains of K_2_ (Lys335/862) and P_3_ (Pro336/Pro863). The role of Leu40 is different in PTP-PEST and Vinculin. In PTP-PEST, due to the flexibility of R_5_ (Arg338) and T_6_ (Thr339), variable nonpolar contacts are formed with the nonpolar parts of their side chains. In Vinculin, however, due to the stability of L_5_ (Leu865) and P_6_ (Pro866), nonpolar interactions with Leu40, Trp43, and Ile54 are conserved.

In most known structures of SH3 domains, at least two out of three ligand binding pockets have hydrophobic/aromatic character, resulting in low binding specificity and therefore relatively high ligand promiscuity. In contrast, the CAS SH3 domain is more polar, due in part to the presence of Glu17 in the second binding pocket. We used PFAM alignment of all human SH3 domains to study the abundance of Glu/Asp residues at the position corresponding to Glu17 in CAS, which permits the binding of the centrally located lysine in the ligand. Within 506 sequences of human SH3_1 domains in the PFAM database, we identified only 12 unique SH3 domains that are likely to bind a centrally located lysine in a manner similar to CAS (Supplementary Figure S5). Of these, seven SH3 domains have glutamate and five have aspartate at the position corresponding to Glu17. The alignment of all human SH3 domains also showed that Leu40, which is important for CAS binding to the ligand, is a CAS-specific feature of the SH3 domain and does not occur in any other human SH3 domain. Interestingly, SH3 domains found by the PFAM search contain two highly conserved aromatic residues localized at the base of the second binding pocket that are substituted with charged amino acids in the CAS SH3 domain (see Supplementary Figure S5; Tyr/Phe to Asn14 and Tyr/Phe to Arg59). This further highlights the uniqueness of the CAS SH3 domain and is potentially responsible for the higher selectivity and affinity of CAS SH3 for ligands with a centrally located lysine.

### CAS SH3 ligand binding is regulated by Src-dependent phosphorylation of Tyr12

**Figure 7:**
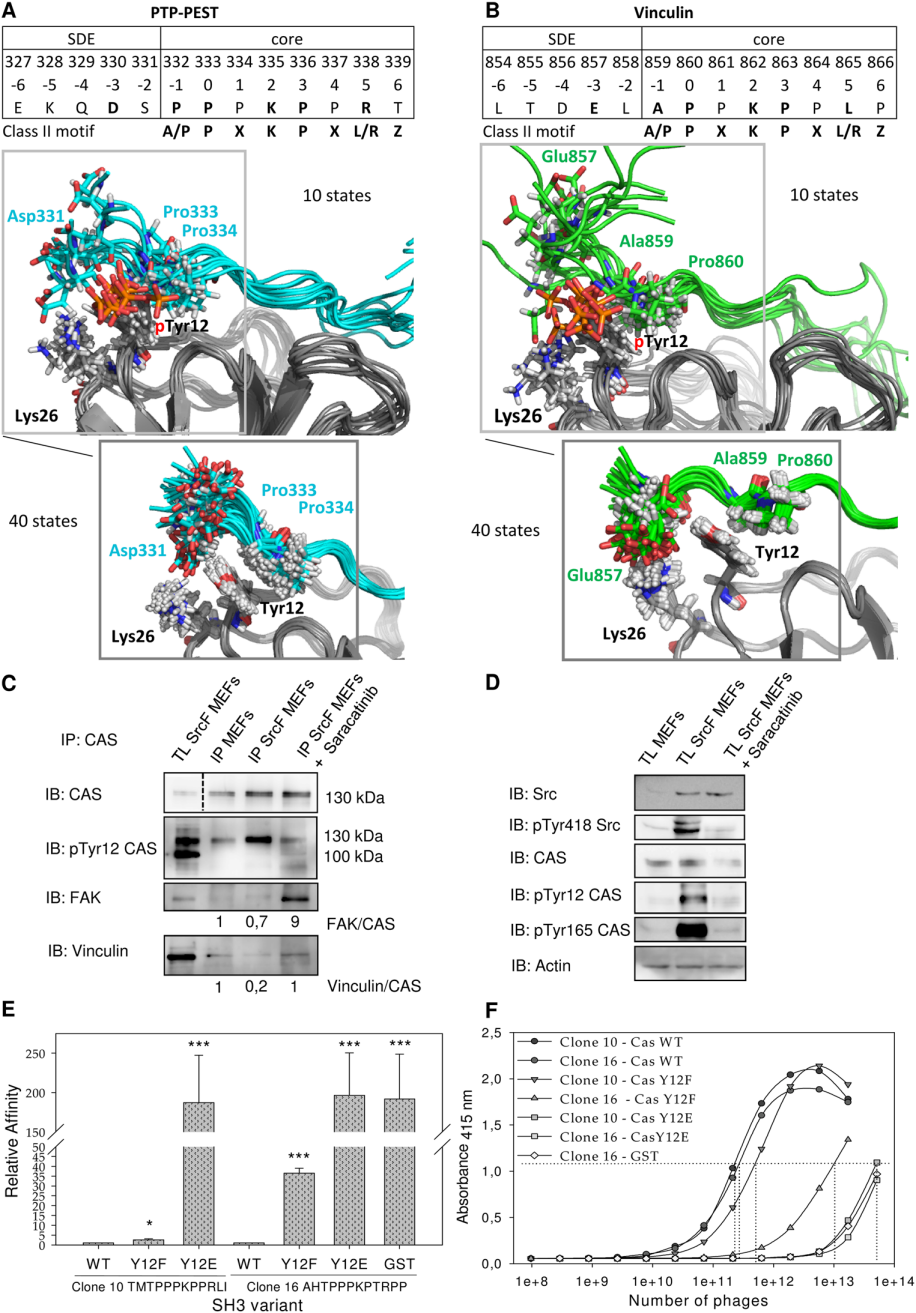
Src phosphorylation of CAS on Tyr12 inhibits CAS binding to FAK and Vinculin. Ten structures from molecular dynamics of phosphoTyr12 CAS SH3 with (**A**) PTP-PEST peptide (cyan) and (**B**) Vinculin peptide (green) are shown. These are compared with 40 NMR structures of unphosphorylated CAS SH3 complexes. (**C**) Co-precipitation analysis of the effect of Src activity on CAS binding capacity. CAS was immunoprecipitated from MEFs and from Src-transformed (SrcF) MEFs (in presence or absence of 5 µM saracatinib), and the amount of co-precipitated FAK and vinculin was analyzed. Numbers indicate the fold-change in ratio of FAK or Vinculin coimmunoprecipitated with CAS. (**D**) Immunoblot analysis of the effect of Src activity on CAS phosphorylation. (**C**,**D**) Blots were cropped from original full-size images (see Supplemental Material). (**E**) Quantification of phage-displayed peptides (clone 10 and clone 16) binding to CAS SH3 variants (WT, Y12F, Y12E) fused with GST or GST alone. The relative affinity represents the binding ratio relative to CAS-WT. The data are expressed as average ± standard deviation from three independent experiments. Statistical significance (*p < 0.05, ***p < 0.001) was determined on log-transformed data by one-way repeated-measures ANOVA followed by Tukey’s post-hoc test. (**F**) Representative curves of phage-displayed peptides (clone 10 and clone 16) binding to CAS SH3 variants (WT, Y12F, Y12E) fused to GST or GST. GST alone with phage clone 16 was used as a negative control.

As we previously suggested^20^, CAS SH3 domain binding and signaling is negatively regulated by phosphorylation of Tyr12. At the structural level, our data indicate that the aromatic ring of Tyr12 is involved in nonpolar interactions with P_−1_/A_−1_ and P_0_ of PTP-PEST/Vinculin (Fig. 5), while its hydroxyl group remains solvent-exposed. The MM-GBSA energy decomposition suggests that Tyr12 is an important stabilizing element of the interaction of CAS SH3 with PTP-PEST/Vinculin (Fig. 6B). We probed the effects of Tyr12 phosphorylation and mutation by molecular dynamics and quantum mechanics (QM). The introduction of the phosphate group adds a −2 charge to the site, and by formation of a salt bridge with Lys26, it disrupts the native Tyr12 to P_0_ and P/A_−1_ CH/π interactions. This is corroborated by QM calculations, which show drops in interaction energy of 21 kcal.mol^−1^ for both PTP-PEST and Vinculin complexes with CAS SH3 upon modification of Tyr12 to phosphorylated Tyr12 (Supplementary Table S8). Furthermore, phosphorylated Tyr12 weakens or displaces the native salt bridges of Lys26 with the conserved Glu857 (E_−3_) for Vinculin and transient Asp330 (D_−3_) for PTP-PEST (Fig. 7A,B), highlighting the importance of acidic upstream SDEs to modulate the effect of phosphorylation on ligand binding.

Though the experimental evidence for the importance of Tyr12 phosphorylation in CAS SH3 binding capacity and CAS signaling is strong, it is based on *in vitro* phosphorylation of isolated SH3 domains or the use of “phosphomimicking” glutamate to substitute Tyr12 (Y12E)^13, 20^. To confirm that Src phosphorylation of CAS on Tyr12 inhibits the SH3 domain-mediated binding capacity of CAS in cells, we compared the binding affinities of CAS to FAK and Vinculin in mouse embryonic fibroblasts (MEFs) and MEFs overexpressing constitutively active Src (MEFs SrcF). Co-immunoprecipitation of Vinculin and partly FAK with endogenous CAS was significantly decreased in MEFs SrcF compared to MEFs (Fig. 7C,D). In addition, inactivation of Src in MEFs SrcF using the Src inhibitor Saracatinib restored the binding of CAS to Vinculin to a similar level as in MEFs. Surprisingly, this inhibition simultaneously stimulated the interaction between CAS and FAK, with over 9-fold higher levels than in MEFs (Fig. 7C,D). Overall, these results confirm that the Src-dependent phosphorylation of Tyr12 serves as a negative regulatory mechanism of the CAS SH3 domain binding.

We also tested the effect of Tyr12 substitution on ligand binding using a phage ELISA approach. For this analysis, we chose phage clones 10 (TMTPPPKPPRLI) and 16 (AHTPPPKPTRPP) because they closely match the sequences of CAS SH3 physiological ligands PTP-PEST and FAK, respectively. As expected, the introduction of the phosphomimicking Y12E mutation led to complete disruption of ligand binding (Fig. 7E,F). This is supported by QM calculations that show a 10–11 kcal.mol^−1^ drop in interaction energy upon Y12E mutation. The small delocalized system of Glu12 carboxylate is not able to form the same CH/π interactions as Tyr12. Surprisingly, however, we found that CAS SH3 Y12F mutant binds to clone 10 with a slightly reduced affinity and to clone 16 with a markedly decreased affinity compared to CAS WT (2- and 37-fold, respectively). The QM data support this observation, as the interaction energies drop by 14 and 1 kcal.mol^−1^ for PTP-PEST and Vinculin, respectively (Supplementary Table S5). The reason for the decrease is likely primarily electronic, i.e, the decrease in dipole moment upon Y12F mutation. The dipole was pinpointed as an important stabilizing contribution for the analogous Trp-Pro interaction^27^.

### DOK7 and GLIS2 are CAS interaction partners

**Figure 8:**
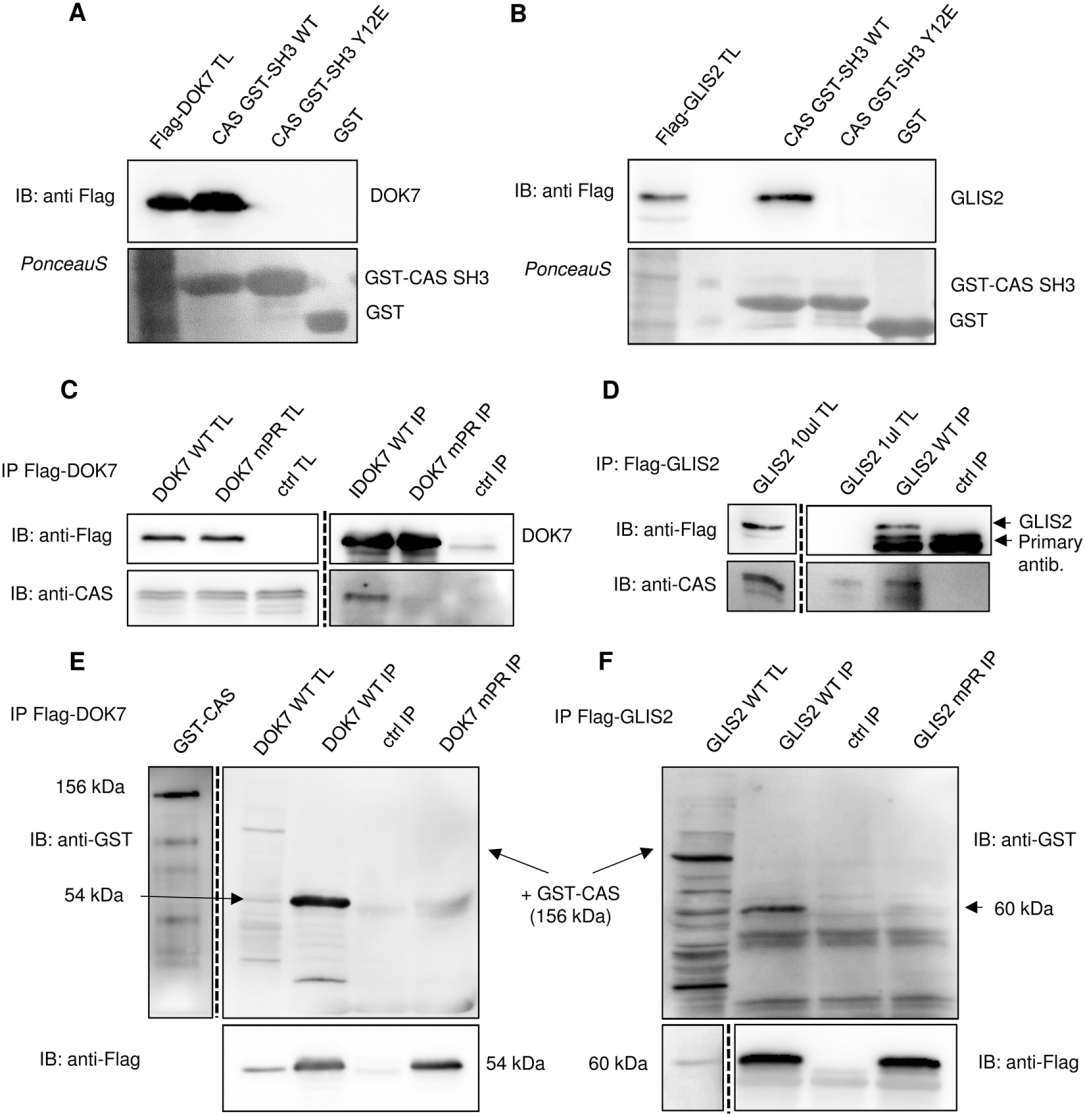
CAS/BCAR1 interacts with DOK7 and GLIS2. (**A**,**B**) Pull-down assays. Binding of (**A**) Flag**-**DOK7 or (**B**) Flag-GLIS2, expressed from transiently transfected MDA-MB-231 cells, to purified CAS SH3 domain variants (WT, Y12E, and Y12F) fused with GST was analyzed by pull-down assays. Pulled-down proteins were immunoblotted with anti-Flag antibody, and GST-SH3 domains were stained with Ponceau-S. (**C**,**D**) MDA-MB-231 cells were transfected with (**C**) Flag**-**DOK7 or (**D**) Flag-GLIS2 followed by immunoprecipitations using anti-Flag sepharose and for DOK7 elution with glycine, pH 3.5. Co-immunoprecipitated CAS/BCAR1 was detected with anti-CAS antibody. In far-Western experiments (**E**,**F**), Flag-DOK7 (**E**) or Flag-GLIS2 (**F**) was immunoprecipitated from transfected MDA-MB-231 cells and eluted with glycine pH 3,5 (DOK7) or by 6x Laemmli sample buffer (GLIS2). Eluted Flag-DOK7 (WT, mPR) or Flag-GLIS2 (WT, mPR) were then transferred to nitrocellulose membrane and incubated with recombinant CAS/BCAR1-GST. The binding CAS/BCAR1-GST was detected with anti-GST antibody. TL: total cell lysate; IP: immunoprecipitation; Ctrl: control samples prepared from untransfected MDA-MB-231 cells. Blots were cropped from original full-size images (see Supplemental Material).

To increase understanding of CAS signaling, we scanned the identified (**A**/**P**)_−1_ **P**_**0**_ × _1_ **K**_**2**_ **P**_**3**_ × _4_(**L**/**R**)_5_Z_6_ CAS SH3 binding motif in the UNIPROT SWISSPROT human/mouse protein database using PATTINPROT and BLASTP programs and identified 10 potential new interacting partners of CAS (Supplementary Figure S6A). After using published data to conduct a thorough evaluation of 10 potential partners for their possible connection to signaling processes involving CAS/BCAR1^28^ (Supplementary Figure S6B,C), we selected **DOK7**, which contains the high affinity P_509_ **P**P**KP**L**R**P motif, and **GLIS2**, with an anchoring leucine at position +5 (P_333_ **P**P**KP**P**L**P), as the most promising candidates. To analyze the interactions of CAS SH3 with DOK7 and GLIS2, we performed a pull-down assay with GST-fused Tyr12 variants of the CAS SH3 domain. We confirmed that both DOK7 and GLIS2 interact with the CAS SH3 domain and that these interactions are blocked by substituting Tyr12 with a phosphomimicking glutamate (Fig. 8A,B). Co-immunoprecipitation experiments suggested that DOK7 and GLIS2 bind full-length CAS (Fig. 8C,D). In addition, we mutated the CAS SH3 binding motifs in DOK7 and GLIS2 to PAPVALR or PAPVAPLP, respectively, and performed co-immunoprecipitation for DOK7 and far-Western experiments for both DOK7 and GLIS2 (Fig. 8C,E,F). These experiments confirmed that the CAS-DOK7 and CAS-GLIS2 interactions are direct and require intact CAS SH3 binding motifs.

## Discussion

We used a combination of experimental and computational methods to reveal the ligand preferences of the CAS SH3 domain. Using phage display, we described the CAS SH3 high affinity binding motif as A/P_−1_P_0_ × _1_K_2_P_3_ × _4 _L/R_5_Z_6_. This motif, which requires an uncommonly localized lysine residue at position +2 and either leucine or arginine at position +5, is present in nearly all known CAS SH3 ligands (Fig. 3B). In addition, several proteins, including PTP1B^15^, CD2AP^14^, Ack1^29^, Dynamin1^30^, and Dock180^31^, are annotated to bind the CAS SH3 domain *via* a centrally located arginine (position +2) instead of lysine. Therefore, it seems that arginine at position +2 is also acceptable, although our phage display did not identify it, perhaps due to its less effective binding contribution than lysine.

To understand the molecular basis of the CAS SH3 domain ligand specificity and regulation of binding, we determined solution structures of the chimeric CAS SH3 domain fused *via* a flexible linker to PTP-PEST or Vinculin-derived binding peptides. Both interactions are functionally well-described and their core 8-amino-acid binding sequences differ in the anchoring residue at position +5, with arginine in PTP-PEST and leucine in Vinculin^10, 13^. Binding of PTP-PEST/Vinculin peptides to the CAS SH3 domain leads to repositioning of the RT loop (i.e., Asn14, Glu17, Glu21, and Asp20) towards the ligand (Fig. 4B,C). The NMR chemical shift perturbation data revealed differences in induced changes for Glu17 and Asp20 of PTP-PEST compared to Vinculin (Supplementary Figure S3) that can be explained by a less restricted movement of Glu17 and increased stability of Asp20 in the case of PTP-PEST. The contributions of Glu17 and to some extent Glu21 for PTP-PEST binding are more prominent than for Vinculin because they bind not only the central lysine but also an anchoring arginine (Figs 5 and 6). In summary, our data suggest that the presence of the centrally located lysine in CAS SH3 ligands and its binding to Glu17 and 21 undermines the anchoring role of arginine and consequently allows for its effective substitution by leucine.

### High-affinity binding ligands featuring a central lysine are unique for CAS SH3

A centrally located lysine has also been observed in a Vinculin-derived peptide bound to the SH3 domain of CAP protein^32^; however, it displayed a markedly lower (17-fold) binding affinity compared with Vinculin bound to the CAS SH3 domain.

Although lysine from Vinculin is in contact with Asp881 of CAP SH3, the distance and orientation of interacting residues is less favorable than in the case of lysine binding to Glu17 in CAS SH3. However, a detailed analysis of the ligand binding to CAS and CAP suggests that the presence of glutamate or aspartate at the position equivalent to Glu17 in CAS permits binding of a centrally located lysine in ligands. Our analysis of human SH3 domains showed that there are only 13 unique SH3 domains with glutamate or aspartate at the position corresponding to Glu17 out of the 506 sequences of human SH3_1 domains in the PFAM database (Supplementary Figure S5). None of them possesses the other CAS SH3 specific amino acids (Asn14, Arg59, Leu40) that are potentially critical for the CAS SH3 domain binding specificity. Asn14 locks Glu21 and Arg59 *via* intramolecular bidentate H-bonds into an optimal position for ligand binding and is likely responsible for the tolerance of Arg59 close to the centrally located lysine residue at +2, which forms salt bridges with Glu17 and Glu21 (Fig. 5, Supplementary Tables S4 and S7).

### Identification of an extended binding surface on CAS SH3

In addition to amino acids that interact with the typical SH3 binding pockets, other residues contribute significantly to CAS SH3-Vinculin and CAS SH3-PTP-PEST binding. Negatively charged amino acids preceding the polyproline ligand core form salt bridges or H-bonds with Lys26 (Supplementary Tables S4 and S7). Interestingly, the N-terminal SDEs from the CAS SH3 domain interaction partners are often enriched with Glu or Asp residues (Supplementary Figure S4). In our structures, Lys26 forms salt bridges in more than 90% of converged structures with Glu857 at position −3 from Vinculin, while the incidence of H-bonds between Asp330 from PTP-PEST at the same position is lower (23% of structures) (Supplementary Tables S2–7). This is in an agreement with the MM-GBSA energy calculations that attributed a higher attractive energy contribution to Lys26 for CAS SH3-Vinculin binding than CAS SH3-PTP-PEST (Fig. 6B). In the PTP-PEST chimera, the C-terminal SDE also contributes to the binding (Fig. 6A, Supplementary Tables S2–4). In particular, Arg340 forms salt bridges and H-bonds with Gly39, Gln38, or Asp41 (Supplementary Table S4) that are analogous to interactions observed in the complex of Src-SH3 with the high affinity peptide APP12 (PDB code 4RTY, ref. 33). A C-terminal extension of the core APPLPPR motif by NRPRL (class II ligand) leads to a 20-fold increase in affinity^33^.

### New model for Src-mediated phosphorylation of CAS

**Figure 9:**
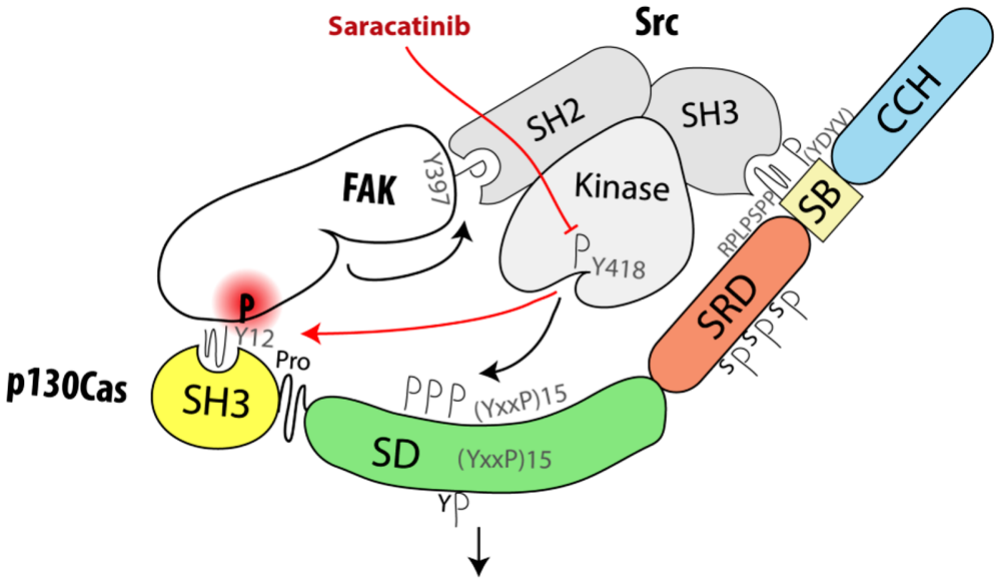
Model of Src-mediated regulation of CAS-FAK association. Upon activation, Src associates with both FAK and CAS through its SH2 and SH3 domains, respectively. This brings Src kinase domain into close contact with CAS SD, leading to its phosphorylation and activation of downstream signaling. With lower dynamics^13^, Src also phosphorylates Tyr12 (Y12) within CAS SH3 domain, which prevents its further binding to FAK and potentially leads to dissociation of the ternary CAS-FAK-Src complex. In contrast, when Src kinase is inhibited, the formation/stabilization of the ternary complex is enhanced.

Previous work suggested that CAS SH3 domain signaling is regulated by phosphorylation of Tyr12^20^. By manipulating the levels of Src kinase expression and its activity, we confirmed that phosphorylation of Tyr12 in cells is mediated by Src and that this phosphorylation correlates with a reduction of CAS-FAK and CAS-Vinculin binding (Fig. 7C,D). We found that FAK binding to CAS is significantly increased after Src inhibition, which can be explained by a modified combination of two mechanisms of Src-mediated phosphorylation of CAS^34^: (i) an indirect Src/FAK cooperative mechanism in which CAS bound *via* its SH3 domain to FAK becomes phosphorylated by Src bound to FAK *via* its SH2 domain^35^, and (ii) direct binding of Src mediated mainly by its SH3 domain to the CAS Src-binding domain^34^. We propose that CAS, FAK, and Src can form a ternary complex mediated by: (i) CAS SH3 domain binding to FAK, (ii) Src SH2 domain binding to FAK, and (iii) Src SH3 domain binding to CAS (see model in Fig. 9). Formation of the ternary complex explains our observation that in Src-transformed MEFs, the CAS-FAK association, unlike the CAS-Vinculin interaction, is enhanced by Src kinase inhibition to levels over nine-fold higher than in untreated MEFs (see Fig. 7C). The inhibition of Src kinase prevents CAS-FAK dissociation induced by CAS Tyr12 phosphorylation, and at the same time the presence of Src stabilizes the CAS-FAK association by formation of the ternary complex. Therefore, Tyr12 phosphorylation in the CAS SH3 domain appears to be a key regulator of CAS signaling, determining the dynamics and sustainability of CAS-mediated signals. However, Src is likely not the only kinase responsible for Tyr12 CAS SH3 phosphorylation. Bmx kinase also has been shown to phosphorylate Tyr12 *in vitro* ^20^.

### The role of CAS in DOK7 and GLIS2 signaling

We performed a proteome-wide search for CAS SH3 binding motifs and identified DOK7 and GLIS2 as novel putative CAS SH3 binding partners (Supplementary Figure S6A). The CAS-binding sequences of DOK7 and GLIS2 differ only at the anchoring amino acid at position +5 (leucine and arginine for DOK7 and GLIS2, respectively). Both DOK7 and GLIS2 were predicted to play a role together with CAS/BCAR1 within the same protein-protein interaction and functional network (Supplementary Figure S6B,C). While CAS is ubiquitous in all tissues, DOK7 is preferentially expressed in skeletal muscle and heart, and its binding to Crk is important for neuromuscular synapsis formation^36^. Crk binding to either DOK7 or CAS is phosphorylation dependent^36, 37^. DOK7 also has been implicated in the exercise-stimulated expansion of muscle fibers, which could be related to the mechanosensing function of CAS^38^.

Analysis of the potential functional interactions between CAS/BCAR1 and GLIS2 showed that both are implicated in kidney function regulation and may regulate kidney cell planar polarity^39, 40^. GLIS2, also referred to as Nephrocystin 7, is a zinc finger transcription factor. Loss of GLIS2 function in humans and mice leads to development of nephronnophthisis, a recessive cystic kidney disease^40^ caused by a mutation in one of the nine nephrocystin genes. Interestingly, Nephrocystin 1 and 4 interact with CAS^39, 41^. In contrast to CAS, GLIS2 is mainly localized in the nucleus, but in cytosol it interacts with p120catenin and promotes p120catenin nuclear localization^42^. Similarly, CAS has previously been shown to bind nucleocytoplasmic zing finger protein CIZ^12^.

## Conclusions

We determined the consensus CAS SH3 binding motif and analyzed in detail structural aspects of the CAS SH3 domain binding. We revealed the requirement for an uncommon centrally localized lysine residue at position +2 of CAS SH3 binding peptides and two optional anchoring residues with different properties (leucine or arginine) at position +5. We further expanded the knowledge of CAS SH3 ligand binding regulation by Src-mediated phosphorylation of Tyr12 and confirmed its negative role in ligand binding. Finally, we identified two novel CAS SH3 binding partners, DOK7 and GLIS2.

## Materials and Methods

### Protein expression and purification

The CAS SH3 domain variants were expressed as fusion proteins with glutathione S-transferase from a pGEX-2TK expression system in *Escherichia coli* BL21 (DE3). Bacteria were grown in LB medium with 100 mg/ml ampicillin, and protein expression was induced with 0.4–1 mM isopropyl-b-thiogalactopyranoside (IPTG) at 25 °C overnight or 37 °C for 1.5 h. Uniformly^15^N and/or^13^C-labeled GST-fused CAS SH3 domain was grown in minimal medium supplemented with 1 g/l (^15^NH_4_)_2_SO_4_ and/or 4 g/l ^13^C glucose (Cambridge Isotope Laboratories). Bacterial pellets were suspended in PBS, pH 7.4, containing 0.05% 2-mercaptoethanol and supplemented with bacterial inhibitors MixB (SERVA) and lysed with a French pressure cell press. Proteins were then solubilized by addition of 20% TritonX–100 to a final concentration of 1% and incubated at −20 °C overnight. GST-SH3 fusion protein was purified using Pierce Glutathione Agarose (Thermo Scientific). Bound GST-SH3 was eluted with 10 mM glutathione in 50 mM Tris, pH 8.0, for ELISA experiments. The GST-fused SH3 domain concentration was determined by absorbance at 280 nm using an extinction coefficient of 58,500 M^−1^ cm^−1^. Extinction coefficients were calculated using the Protparam tool (ExPasy). For NMR measurements, the GST tag was removed by thrombin (Sigma) cleavage and subsequent size exclusion chromatography (Sephadex–75, Amersham Biosciences). Two additional amino acid residues remained present at the N-terminus of CAS SH3 after thrombin cleavage. PBS was subsequently exchanged for NMR buffer (25 mM sodium phosphate, pH 7.5, 100 mM NaCl, 1 mM TCEP, 0.01% NaN_3_) with an Amicon Ultra–15 Centrifugal Filter Unit (3 K, EMD Millipore). The purity of the proteins was confirmed by SDS-PAGE.

### Library screening (Phage display)

Panning of a phage display library comprising 10^12^ M13 phages (Ph.D.™ −12 Phage Display Peptide Library Kit, New England Biolabs) carrying 12-amino-acid degenerate oligopeptides was performed according to the manufacturer’s protocol. Briefly, approximately 0.5 mg purified GST-fused CAS SH3 domain (isolated from 40 ml bacterial culture) bound to Pierce Glutathione Agarose (Thermo Scientific) was incubated with blocking buffer (100 mM NaHCO_3_, pH 8.6, 5 mg/ml BSA) and then with 10^11^ infectious particles from the phage display library. After washing 10 times with TBS containing 0.1–0.5% Tween 20, the bound phages were eluted with 200 mM glycine HCl, pH 2.2. After four selection cycles, the 20 bound clones were isolated and sequenced.

### Enzyme-linked immunosorbent assay (ELISA)

The relative binding affinities of individual phage clones to GST-CAS SH3 were assessed by ELISA according to the manufacturer’s protocol (Ph.D.™ −12 Phage Display Peptide Library Kit, New England Biolabs). Briefly, a dilution series of phage stocks was prepared in TBS with 0.5% Tween 20 (TBST, 10^15^−2 × 10^6^ pfu/ml), and phages were scored for their ability to bind immobilized GST-CAS SH3 domain (100 µl of 50 µg/ml per well) in 96-well plate. Bound phages were detected with anti-M13 antibody conjugated to HRP and quantified in a colorimetric assay (ABTS at 415 nm, Invitrogen) using Varioskan Flash (Thermo Scientific). Relative binding affinities (the affinity of the phage clone with the highest affinity was arbitrarily set to 1) were evaluated based on the number of phages required to bind the immobilized GST-fused CAS SH3 domain at 50% saturation. Curves were fitted with the standard sigmoidal function in Sigmaplot. The clones with highest affinities were selected for further analyses (e.g., binding to immobilized 5 µg of CAS SH3 variants per well).

The EC_50_ for phage clone 1 (FHAPPSKPPLPK, highest affinity) was determined using competitive displacement of the phage bound (5.5·10^10^ pfu) to immobilized GST-fused CAS SH3 domain (100 µl of 20 µg/ml per well to yield the best specificity/sensitivity ratio) by serial additions of free GST-fused CAS SH3 domain in TBST (1 µg/ml–800 µg/ml) as previously described^43^. The amount of bound phages was assessed with anti-M13 antibody conjugated to HRP and quantified in a colorimetric assay. Displacement curves were fitted to standard sigmoidal function and EC_50_ was calculated in Sigmaplot version 11 software using a four-parameter logistic equation. Statistical significance (*p < 0.05, **p < 0.01, ***p < 0.001) was determined on log-transformed data by one-way ANOVA followed by Tukey’s post-hoc test.

### Isothermal titration calorimetry (ITC)

ITC experiments were performed using a MicroCal Auto-iTC200 System (GE Healthcare) at 25 °C. The samples were prepared in PBS, pH 7.5, supplemented with 0.05% 2-mercaptoethanol. Protein and peptide concentrations were determined by amino acid analysis. Four synthetic peptides (PPPKPPRL, APPKPPLP, PPSKPPLP, and PPSKPPRP) were used for titration experiments, and their concentrations in PBS were adjusted to 660 μM. For peptide–CAS SH3 complex formation, 2 μl aliquots of 660 μM peptide solution were injected stepwise into a sample cell containing 200 μl of 60 μM CAS SH3. Each assay was accompanied by a control experiment in which the binding buffer was titrated with the injected peptide alone. The dilution heat values obtained for the control titration were then subtracted from those obtained for the complex formation. All experiments were performed at least in triplicate. The association constants and stoichiometry (N) were estimated using MicroCal Origin software (GE Healthcare). Known variances of each measurement were utilized to calculate the weighted average of the dissociation constant (K_*d*_) and stoichiometry as a maximum likelihood estimator. The standard deviation was determined from at least three independent experiments.

### NMR spectroscopy and structure determination

NMR spectra were acquired at 25 °C on 600 MHz and 850 MHz Bruker Avance spectrometers, both of which were equipped with a triple-resonance (^15^N/^13^C/^1^H) cryoprobe. The sample volume was 0.35 ml, with a 450 μM concentration of CAS SH3 and CAS SH3 chimeras (sequences in Supplementary Figure S7) in NMR buffer (25 mM sodium phosphate, pH 7.5, 100 mM NaCl, 1 mM TCEP, 0.01% NaN_3_), 5% D_2_O/95% H_2_O. A series of double- and triple-resonance spectra^44, 45^ were recorded to determine essentially complete sequence-specific resonance backbone and side-chain assignments. Constraints for^1^H–^1^H distance required to calculate the structure of the CAS SH3 domain and CAS SH3 chimeras were derived from 3D^15^N/^1^H NOESY-HSQC and^13^C/^1^H NOESY-HMQC, which were acquired using a NOE mixing time of 100 ms.

The families of converged structures for the CAS SH3 domain and CAS SH3 chimeras were initially calculated using Cyana 2.1^46^. The combined automated NOE assignment and structure determination protocol was used to automatically assign the NOE cross-peaks identified in NOESY spectra and to produce preliminary structures. In addition, backbone torsion angle constraints, generated from assigned chemical shifts using the TALOS + program^47^ were included in the calculations. Subsequently, five cycles of simulated annealing combined with redundant dihedral angle constraints were used to produce sets of converged structures with no significant restraint violations (distance and van der Waals violations <0.2 Å and dihedral angle constraint violation <5 °), which were further refined in explicit solvent using the YASARA software with the YASARA forcefield^48^. The structures with the lowest total energy were selected. Analysis of the family of structures obtained was carried out using the Protein Structure Validation Software suite (www.nesg.org) and Molmol^49^. The statistics for the resulting structures are summarized in Supplementary Table S1. Chemical shift perturbations divided by standard deviation (relative CSP) were plotted against amino acid sequence to highlight the differences between PTP-PEST and Vinculin complexes (Supplementary Figure S3).

### Analyses of NMR structures

#### Interface analysis

Hydrogen bonds and nonpolar contacts between Cas SH3 and its ligands were assessed with PISA^26^ with default settings. Prior to analysis, the linker sequence (residues 75–83) were removed from the chimeric proteins. All structure figures were generated using PyMOL (Schrödinger, LLC).

#### Interaction energies – Molecular mechanics

Energy contributions of CAS SH3 and PTP-PEST/Vinculin residues toward binding were calculated using the MM-GBSA method^50^ in AMBER14^51^ on 40 fragmented NMR structures for each ligand. AMBER ff14SB force field^52^ was used for the protein and ligands. The generalized Born model of Tsui and Case (igb = 1) was used for the polar solvation energies^53^, and a solvent-accessible surface-area-dependent term with SURFTEN = 0.0072 and SURFOFF = 0.0 parameters was employed for nonelectrostatic solvation free energies^53, 54^. Debye-Hückel screening with 150 mM ionic strength was used. The interaction energies were decomposed on a per-residue basis so that 1–4 interactions were added to electrostatic and van der Waals contributions.

#### Interaction energies – Quantum mechanics

Tyr12-mutated variants of CAS SH3 in complex with PTP-PEST and Vinculin were derived from the first NMR models with the linker removed. The side chains were modeled with the LEaP module of AMBER14^51^, and the entire complexes were optimized using PM6-D3H4, a semiempirical quantum mechanical (SQM) method corrected for dispersion and hydrogen bonding^55^, and the COSMO implicit solvent model with ε_r_ = 78.4 to mimic the solvent^56^. The MOPAC2016 program with the MOZYME linear scaling algorithm was employed^57^. The interaction energy was calculated on the optimized structures as the difference between the energy of the complex and the sum of the energies of the constituents. The calculations were performed within the Cuby4 framework^58^.

#### Molecular dynamics

The first fragmented NMR model for each PTP-PEST and Vinculin was turned into a Tyr12-phosphorylated variant by automatic building in the LEaP module of AMBER14^51^ using published phosphotyrosine parameters^59^. The system was prepared and run according to a published protocol^60^ with the following changes: the numbers of Cl^−^/Na^+^ counterions added to neutralize the system and match the physiological concentration of 0.15 M were 15/15 and 13/18, respectively, and the production run was 50 ns.

### Motif definition and sequence alignment

The precomputed sequence alignment of all human SH3_1 domains from the PFAM database^61^ was filtered for obsolete entries and manually edited to identify conserved positions relevant to ligand binding. Sequences from phage display or from known high-affinity CAS SH3 domain binding ligands were aligned using CLUSTAL 2.1 multiple sequence alignment^62^. The alignments were further analyzed to describe sequence motifs using WebLogo version 3.0^63^.

### Plasmid construction

pEGFP-C1 CAS and pGEX-CAS-SH3 domain constructs were prepared previously^20^. The chimeric CAS SH3 domains with the binding peptide were prepared by fusion of mouse CAS SH3 domain with a flexible spacer sequence (SGGSGSG) and binding peptides derived from PTP-PEST (327–343 of mouse PTP-PEST, UniProtKB accession number P35831) or Vinculin (854–870 of mouse Vinculin, UniProtKB accession number Q64727). cDNA coding for the flexible peptides with the binding peptides was commercially synthesized (geneArt, Life technologies) and inserted in frame at the 3′ end of the CAS SH3 domain using *Bam*HI/*Eco*RI sites. The constructs were verified by sequencing. DOK7 cDNA was PCR-amplified from cDNA isolated from MCF7 human breast carcinoma cells using a forward primer (5′-TACTCGAGATGGACTACAAAGACGATGACGACAAGAAGATGACCGAGGCGGCG-3′) that includes a sequence coding for Flag epitope and a reverse primer (5′-TAGAATTCGCTCTCAAGGAGGGGGGTTTACC-3′). In parallel, GLIS2 cDNA was prepared from the BLM melanoma cell line and amplified by PCR using the following primers: forward 5′-TACTCGAGATGGACTACAAAGACGATGACGACAAGCACTCCCTGGACGAGCCG-3′, reverse 5′-TAGAATTCTCAGTTCACCACAGCCGGT-3′. The PCR-amplified DOK7 and GLIS2 cDNA were cleaved with *Xho*I/*Eco*RI and inserted into *Xho*I/*Eco*RI-cleaved pIRES2-EGFP (Addgene), resulting in plasmids pIRES2-FLAG-DOK7 and pIRES2-FLAG-GLIS2. Constructs were verified by sequencing.

Site-directed mutagenesis of DOK7 and GLIS2 was performed by whole plasmid synthesis using Q5 polymerase (New England Biolabs) with respective primers (DOK7 mPR: forward 5′-TGGTGGGTGCCTCAAGGCCAGCACCGGTAGCGCTGCGTCCGCGG-3′, reverse 5′-CCGCGGACGCAGCGCTACCGGTGCTGGCCTTGAGGCACCCACCA-3′; GLIS2 mPR: forward 5′-CTCCTGCAGCTGCGCCCAGCACCGGTAGCGCCACTGCCCGCC-3′, reverse 5′-GGCGGGCAGTGGCGCTACCGGTGCTGGGCGCAGCTGCAGGAG-3′). pIRES2-FLAG-DOK7 and pIRES2-FLAG-GLIS2 were used as templates. After PCR, 5U of *Dpn*I were added to each reaction and incubated for 1.5 h at 37 °C. Individual mutated clones were selected for the presence of a newly introduced *Age*I site, and final constructs were verified by sequencing.

### Cell transfection and culture

MDA-MB-231 cells (provided by RNDr. Zadinova from Charles University) were cultivated in full DMEM (Sigma) with 4.5 g/l L-glucose, L-glutamine, and pyruvate, supplemented with 10% fetal bovine serum (Sigma). The cells were transfected with pIRES2-FLAG-DOK7 WT/mPR, pIRES2-FLAG-GLIS2 WT/mPR, or pEGFP-C1 CAS constructs using a PEI transfection reagent (Polysciences) according to the manufacturer’s protocol.

### Far-Western-blot analysis, immunoprecipitation, and GST pull-downs

MDA-MB-231 cells transiently transfected with pIRES2-FLAG-DOK7 or pIRES2-FLAG-GLIS2 were lysed in a Triton lysis buffer (50 mM Tris HCl, pH 7.4, with 150 mM NaCl and 1% TRITON X–100). Protein concentrations in lysates were determined using the DC Protein Assay (Bio Rad). Lysates containing 750 µg proteins were incubated overnight (4 °C) with 35 µl of an anti-FLAG M2 affinity gel (Sigma) and washed 3 times with 1 ml ice-cold TBS buffer. Complexes were eluted using 0.1 M glycine, pH 3.5. The eluted proteins were immediately re-equilibrated to neutral pH with 1 M Tris, pH 9.2, and mixed with 6x Laemmli sample buffer and processed for SDS-PAGE. After SDS-PAGE, proteins were transferred onto a nitrocellulose membrane. The membranes were usually cut after the transfer to enable probing for up to 3 proteins of different molecular mass (e.g. 1–2 proteins of interest and a loading control). Nonspecific activity was blocked by incubating the membranes for 90 min at room temperature in TBS containing 4% bovine serum albumin. Membranes were then incubated overnight at 4 °C with a primary antibody, washed extensively with TBS with 0,05% Tween 20 (TBST), incubated for 1 h at room temperature with HRP-conjugated secondary antibody, washed extensively in TBST, and developed using an AI600 System (GE Healthcare). For far-Western-blot analysis, the protein blots were incubated with 2 µg/ml recombinant protein probe (recombinant human BCAR1, Abcam) in 1% BSA in TBST overnight followed by washing with TBST and incubation (2 h, 4 °C) with anti-GST antibody (Sigma). After extensive washing with TBST, blots were treated with HRP-conjugated secondary antibodies and developed using the AI600 System.

The GST-CAS SH3 domain and mutational variants were expressed in bacteria and affinity purified as described above (see “Protein expression and purification” section). Cell lysates from MDA-MB-231 cells transiently transfected with pIRES2-FLAG-DOK7 or pIRES2-FLAG-GLIS2 were incubated with 20 µg of GST or GST-CAS SH3 variants immobilized on Glutathione Agarose (Thermo Scientific) for 2 h at 4 °C. The agarose beads were washed extensively with LB1 buffer (50 mM HEPES, pH 7.4, 150 mM NaCl), boiled in 6x SDS-PAGE sample buffer, and processed for immunoblotting. The nitrocellulose membrane was stained with Ponceau S (for SH3 domain loading); destained and pulled-down DOK7 was detected with anti-Flag antibody.

Immunoprecipitations from lysates of MEFs or MEFs transformed with SrcF were performed similarly as described above. To immunoprecipitate CAS proteins, protein-A sepharose (GE-Healthcare) and anti-CAS antibody (BD Transduction Laboratories) were used. To inhibit Src kinase, cells were treated with 5 µM Saracatinib for 3 h. After transferring proteins onto nitrocellulose membrane and blocking, the membranes were incubated with anti CAS, Src, FAK, Vinculin, CAS Tyr12, CAS pTyr165, Src pTyr418 or actin antibody.

### Antibodies

Anti-M13 fused to HRP (phage display library kit, New England Biolabs), anti-CAS (BD Transduction Laboratories, mouse monoclonal clone 21), anti-GST (G7781, Sigma, rabbit polyclonal), anti-FLAG (Sigma, mouse monoclonal clone M2), anti-FAK (C–20, Santa Cruz Biotechnology, rabbit), anti v-Src (Ab–1, Calibiochem, mouse monoclonal clone 327), anti-Vinculin (Sigma, mouse monoclonal clone V284), anti-CAS pY165 (#4015, Cell Signaling Technology, rabbit polyclonal), anti-Src pY418 (#2101, Cell Signaling Technology, rabbit polyclonal), anti-Actin (C–11, Santa Cruz, goat polyclonal), and secondary antibody/ies fused to HRP (Abcam) were used as purchased. Anti-CAS Y12 antibody was prepared as previously described^20^.

## Electronic supplementary material

Supplementary figures, tables and blots
