# Supplementary material for: Structural characterization of CAS SH3 domain selectivity and regulation reveals new CAS interaction partners
Source: Sci Rep. 2017 Aug 14;7:8057. doi: 10.1038/s41598-017-08303-4 (PMC5556061; doi:10.1038/s41598-017-08303-4)

Supplemental Material for the Manuscript:

Structural characterization of CAS SH3 domain selectivity and regulation reveals new CAS interaction partners

Authors: Jakub Gemperle[1], Rozálie Hexnerová[2], Martin Lepšík[2], Petr Tesina[2], Michal Dibus[1], Marian Novotný[1], Jan Brábek[1], Václav Veverka*[2], Daniel Rosel*[1]

* Correspondence regarding NMR analysis should be directed to Vaclav Veverka (veverka@uochb.cas.cz), correspondence regarding other matters should be directed to Daniel Rosel (rosel@natur.cuni.cz)

[1]Department of Cell Biology, Faculty of Science, Charles University in Prague, Czech Republic
[2]Institute of Organic Chemistry and Biochemistry of the CAS, Czech Republic

Supplemental figures and tables

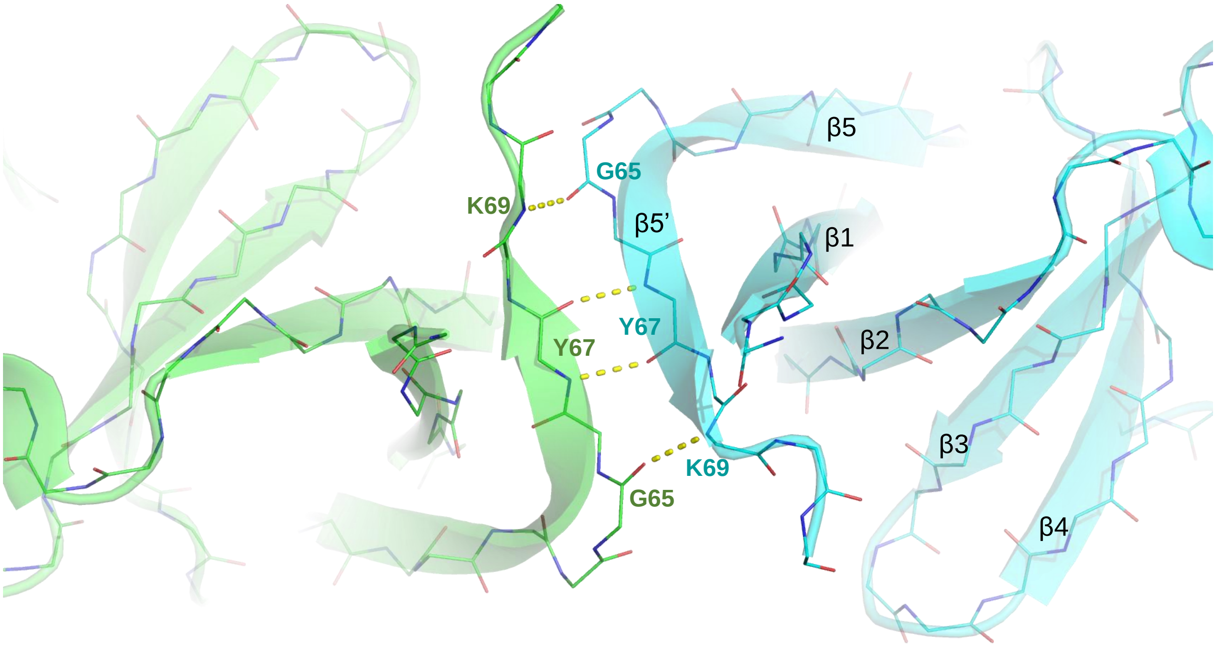

Figure S1. The crystallographic dimer (PDB code 1WYX) is stabilized by the crystal contacts that stimulate the formation of an additional two-stranded (labeled b5’) beta-sheet at the dimer interface. The beta-sheet is stabilized by a network of four intra-main chain hydrogen bonds (highlighted in yellow).

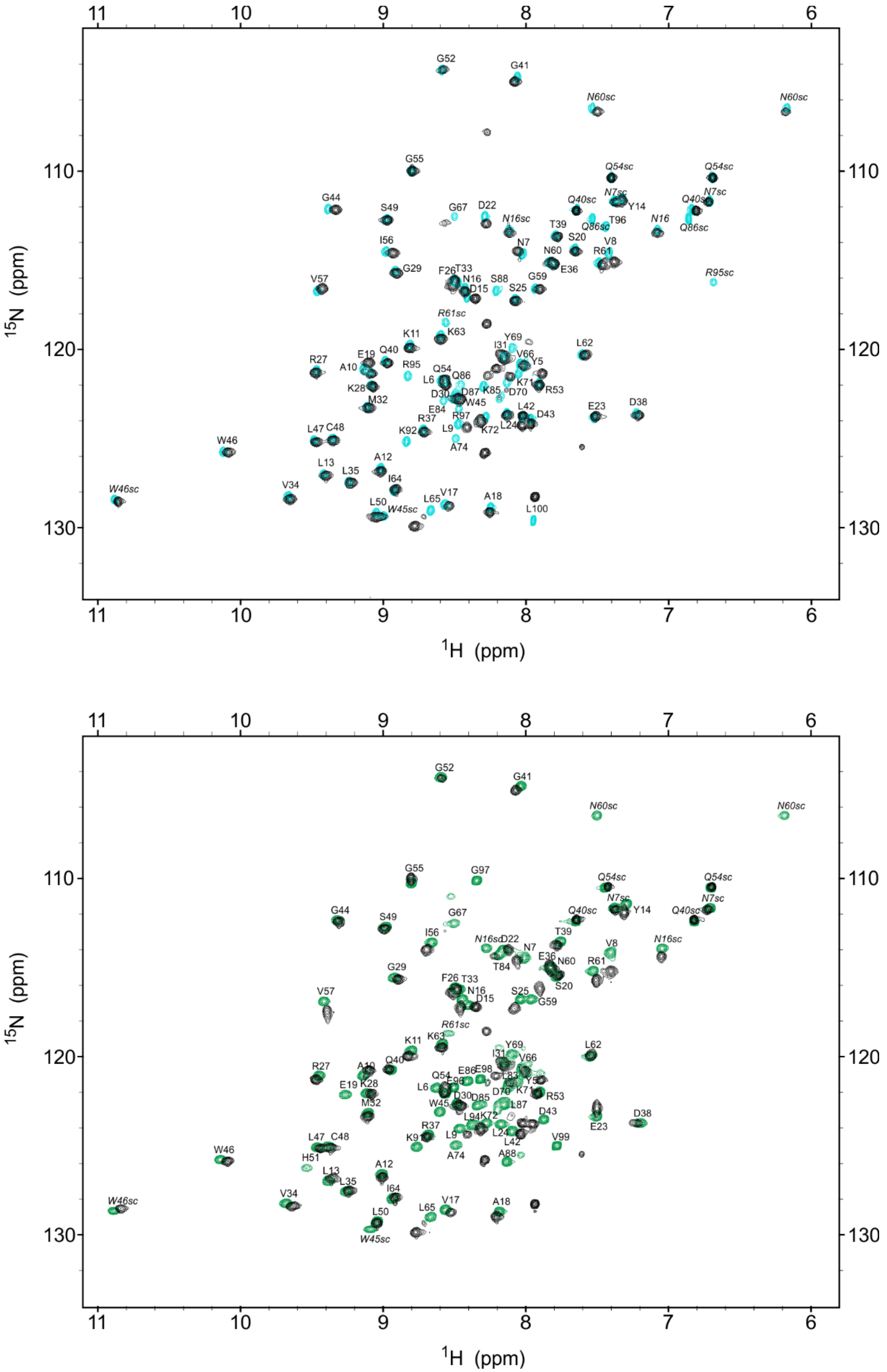

Figure S2. Overlays of ^{15}N/^{1}N HSQC spectra of the free the CAS SH3 domain with bound peptide from PTP-PEST or Vinculin (black) and PTP-PEST (cyan) and Vinculin (green) chimeras.

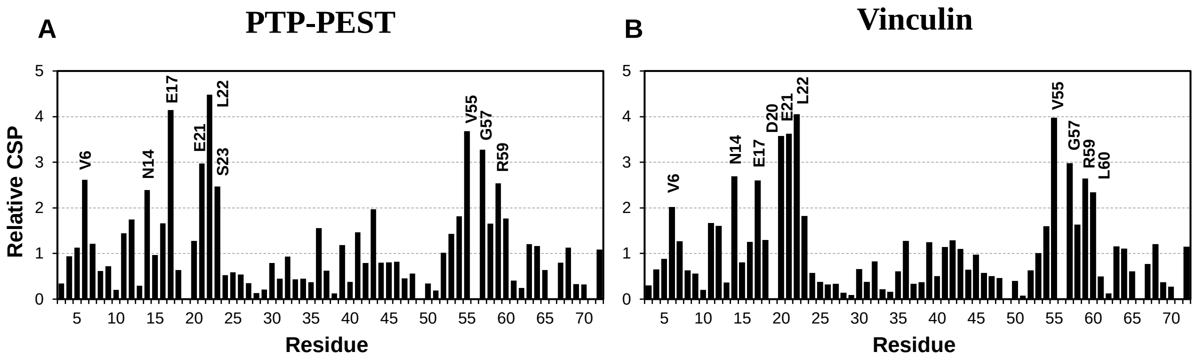

Figure S3. Relative chemical shift perturbations (CSP) of CAS SH3 induced by binding of **A** PTP-PEST peptide and **B** Vinculin peptide. The values were calculated from changes in positions of backbone amide resonances and divided by standard deviation. The distribution of induced changes is similar for both peptides, corresponding to highly overlapping binding regions derived from the structural data obtained for both complexes.

A

	Position
	…-10123456789…
FAK/FRNKb	PDPAAPPKKPPRPGAPGH
PYK2b	LEFTGPPQKPPRLGAQSI
C3G	TDEEVAPPKPPLPGIRVV
Vinculin	LTDELAPPKPPLPEGEVP
FAK/FRNKa	GGSDEAPPKPSRPGYPSP
PYK2a	TAFQEPPPKPSRPKYRPP
PTP-PEST	EKQDSPPPKPPRTRSCLV
MICA1	DPEMEPPPKPPRSCSALA
	* **

B

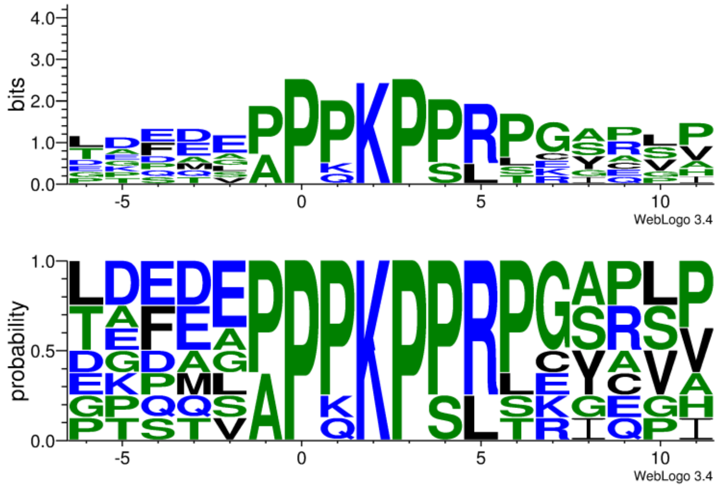

Figure S4. Enrichment of negatively charged amino acids within CAS SH3 binding ligands. **A** Multiple sequence alignment of eight high-affinity CAS SH3 binding sequences derived from known six human CAS SH3 binding proteins using CLUSTAL W. **B** Prolonged high-affinity CAS SH3 binding motif derived from sequences belonging to known human CAS SH3 interacting proteins**.** The x -axis shows the positions in relation to proline (position 0). The y-axis in the upper panel shows the bits. The graphs show the amino acid preference near the proline. The height of the letters is proportional to the frequency in the top eight protein sequences.

```
                              10 12 14 17 20 22 24 26 28 30 32 34 36 38 40 42 44 46 48 50 52 54 56 59
CAS/P56945/9-60              -KALYDNVAES-PDELSFRKGDIMTVLEQDTQgldGWWLCSLH---GRQGIVPGNRL
HCLS1/P14317/434-482         -VAVYDYQGEG-SDELSFDPDDVITDIEMVDE---GWWRGRCH---GHFGLFPANYV
NCF2/P19878/246-294          -RVLFGFVPET-KEELQVMPGNIVFVLKKGND---NWATVMFN---GQKGLVPCNYL
SH3D19/Q5HYK7/501-552        -VVLHDFPAEQ-VDDLNLTSGEIVYLLEKIDT---DWYRGNCR---NQIGIFPANYV
SH3D19/Q5HYK7/577-625        -VARFEYIGEQ-KDELSFSEGEIIILKEYVNE---EWARGEVR---GRTGIFPLNFV
SH3D19/Q5HYK7/667-715        e-ALHSFTAET-SDDLSFKRGDRIQILERLDS---DWCRGRLQD---REGIFPAVFV
SNX9/Q9Y5X1/6-57             -RVMYDFAAEPgNNELTVNEGEIITITNPDVG--gGWLEGRNIK--GERGLVPTDYV
SH3GL1/Q99961/312-360        -KALYDFEPEN-DGELGFHEGDVITLTNQIDE---NWYEGMLD---GQSGFFPLSYV

CAP/Q9BX66/873-923           -IAKFNFNGDT-QVEMSFRKGERITLLRQVDE---NWYEGRIPGT-SRQGIFPITYV
VINEXIN/O60504/460-510       -VAQYTFKGDL-EVELSFRKGEHICLIRKVNE---NWYEGRITGT-GRQGIFPASYV
SORBS2/O94875/944-994        -IAKYNFNADT-NVELSLRKGDRVILLKRVDQ---NWYEGKIPGT-NRQGIFPVSYV
ASAP1/Q9ULH1/1073-1124       k-TIYDCQADN-DDELTFIEGEVIIVTGEEDQ---EWWIGHIEGQpERKGVFPVSFV
ABI1/Q8IZP0/452-500          -VAIYDYTKDK-DDELSFMEGAIIYVIKKNDD---GWYEGVCNR---VTGLFPGNYV
```

Figure S5. Alignment of human SH3 domains that are likely to bind a centrally located lysine in the ligand. Numbers indicate the primary amino acid sequence of the CAS SH3 domain. The alignment includes 13 unique SH3 domains from 11 proteins that include either Glu (yellow column) or Asp (green column) at the position equivalent to Glu17 in CAS protein and a conserved Glu that corresponds to CAS Glu21 (orange column). Unique sequence features of CAS, namely Asn14, Leu40 and Arg59, are also highlighted. Amino acids in small letters indicate positions in the alignment that are occupied in a small proportion of sequences.

A

Prot. name	Sequence	Interaction with SH3 p130Cas
C15oRf62	A_{78}**P**P**KP**P**R**L	?
C1oRf168	P_{278}**P**P**KP**S**R**P	?
CEP2	A_{126}**P**P**KP**P**R**L	?
**DOK7**	P_{317}**P**P**KP**L**R**P	?
FBN2	P_{33}**P**P**KP**P**R**P	?
**GLIS2**	P_{333}**P**P**KP**P**L**P	?
MICAL1	P_{829}**P**P**KP**P**R**S	Known
PKN3	P_{509}**P**P**KP**P**R**L	?
PRAM1	P_{501}**P**A**KP**P**L**P	?
FAK	A_{711}**P**P**KP**S**R**P	Known
PYK2	P_{713}**P**P**KP**S**R**P	Known
PTP-PEST	P_{333}**P**P**KP**P**R**T	Known
C3G	A_{266}**P**P**KP**P**L**P	Known
SETD5	P_{606}**P**A**KP**S**R**P	?
Vinculin	A_{859}**P**P**KP**P**L**P	Known
WNK1	A_{1750}**P**S**KP**P**L**T	?

B

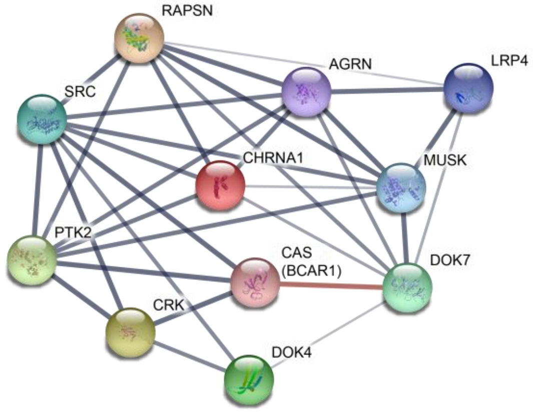

C

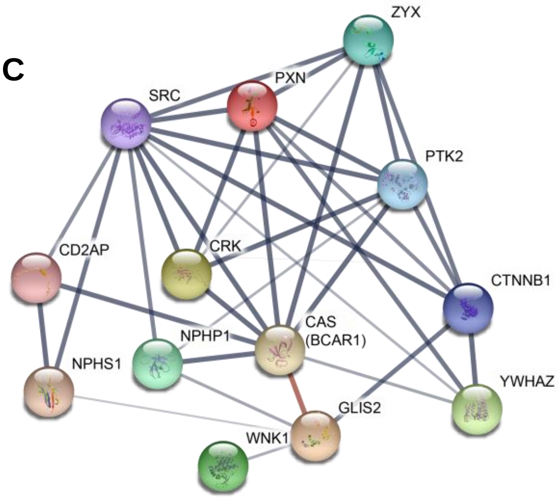

Figure S6. Potential CAS/BCAR1 interaction partners. **A** Table of known and predicted CAS SH3 interacting proteins based on CAS SH3 binding motif. Six have already been shown to bind CAS. In proteins highlighted by gray boxes, we were able to find connections to the signaling processes involving CAS. **B** Signaling network of proteins that are important for acetylcholine receptor clustering in skeletal muscle with added CAS. The red line represents the new **CAS-DOK7** interaction. **C** Signaling network of proteins implicated in regulation of kidney function. The red line represents the new **CAS-GLIS2** interaction. Crosstalk is illustrated with STRING 10 (http://version_10.string-db.org/). Line thickness indicates the strength of data support, a red line represents a newly verified interaction.

CAS_SH3

Amino acid sequence after thrombin cleavage:
GSMKYLNVLAKALYDNVAESPDELSFRKGDIMTVLERDTQGLDGWWLCSLHGRQGIVPGNRLKILVGMYDKKPAG*EFIVTD*

CAS_SH3_Vinculin

Amino acid sequence after thrombin cleavage:
GSMKYLNVLAKALYDNVAESPDELSFRKGDIMTVLERDTQGLDGWWLCSLHGRQGIVPGNRLKILVGMYDKKPAGSGGSGS
GLTDELAPPKPPLPEGEV

CAS_SH3_PTP-PEST

Amino acid sequence after thrombin cleavage:
GSMKYLNVLAKALYDNVAESPDELSFRKGDIMTVLERDTQGLDGWWLCSLHGRQGIVPGNRLKILVGMYDKKPAGSGGSGS
GSEKQDSPPPKPPRTRSCL

Figure S7. Primary sequences of the CAS SH3 domain chimeras. The first two amino acids (GS) are residuals from the thrombin cleavage site. Blue indicates the extended sequence from cloning vector ended by introduction of a stop codon from *Eco*RI restriction site. Red represents the inserted sequence of the linker. Sequences from the ligands are underlined.

Supplementary table 1. NMR restraints and structural statistics

	SH3	*PTP-PEST*	*Vinculin*
Non-redundant distance and angle constrains			
Total number of NOE constraints	1165	1998	2483
Short-range NOEs			
Intra-residue (i = j)	208	426	494
Sequential (| i - j | = 1)	305	468	585
Medium-range NOEs (1 < | i - j | < 5)	122	183	267
Long-range NOEs (| i - j | ≥ 5)	530	921	1137
Torsion angles	100	80	88
Hydrogen bond restrains	-	-	-
Total number of restricting constraints	1265	2078	2571
Total restricting constraints per restrained residue	17.6	23.9	28.9
Residual constraint violations			
Distance violations per structure			
0.1 – 0.2 Å	3.45	9.25	8.40
0.2 – 0.5 Å	2.22	3.65	3.80
> 0.5 Å	0	0	0
r.m.s. of distance violation per constraint	0.02 Å	0.02 Å	0.02 Å
Maximum distance violation	0.50 Å	0.50 Å	0.50 Å
Dihedral angle violations per structure			
1 – 10 °	2.33	1.23	0.9
> 10 °	0	0	0
r.m.s. of dihedral violations per constraint	0.51 °	0.46 °	0.31 °
Maximum dihedral angle violation	5.00 °	5.00 °	4.90 °
Ramachandran plot summary from Procheck			
Most favoured regions	96.4%	92.3%	93.1%
Additionally allowed regions	3.6%	7.5%	6.7%
Generously allowed regions	0.0%	0.1%	0.1%
Disallowed regions	0.0%	0.0%	0.0%

r.m.s.d. to the mean structure	*ordered*[1]	*all residues*	*ordered*[1]	*all residues*	*ordered*[1]	*all residues*
All backbone atoms	0.3 Å	6.7 Å	0.3 Å	2.7 Å	0.2 Å	2.4 Å
All heavy atoms	0.9 Å	6.9 Å	0.7 Å	3.0 Å	0.5 Å	2.6 Å

[1] Residues with sum of phi and psi order parameters > 1.8

| per 10% of BSA	BSA = burried of accessible surface area
-	
|- ||	
||| - ||||	
||||| - ||||||	
|||||||-||||||||||	

SDE	Pocket S1	Pocket S2	Pocket S3	SDE (ext. S3)
Leu11				
Tyr12	Tyr12			
Asp13				
		Asn14		
		Val15		
		Glu17	Glu17	
			Ser18	
				Pro19
			Asp20	Asp20
		Glu21	Glu21	
Lys26				
				Gln38
				Gly39
			Leu40	Leu40
				Asp41
				Gly42
		Trp43	Trp43	
		Trp44	Trp44	
			Ile54	
		Pro56		
		Gly57		
	Asn58	Asn58		
	Arg59	Arg59		

PTP-PEST

SDE	-6	327	E	
	-5	328	K	
	-4	329	Q	
	-3	330	D	HS
	-2	331	S	
core	-1	332	**P**	
	0	333	**P**	H
	1	334	P	H
	2	335	**K**	HS
	3	336	**P**	
	4	337	P	
	5	338	**R**	
	6	339	T	
SDE	7	340	R	HS
	8	341	S	
	9	342	C	
	10	343	L	

Numbering from UNIPROT: X-2 — **PEST**

CAS SH3-(PTP-PEST) — BSA/ ΔG/ HSDC

UNIPROT	Interface residue	01_PEST	02_PEST	03_PEST	04_PEST	05_PEST	06_PEST	07_PEST	08_PEST	09_PEST	10_PEST
1	Met 3	0.00 (0.00)	0.00 (0.00)	0.00 (0.00)	0.00 (0.00)	0.00 (0.00)	0.00 (0.00)	0.00 (0.00)	0.00 (0.00)	0.00 (0.00)	0.00 (0.00)
2	LYS 4	0.00 (0.00)	0.00 (0.00)	0.00 (0.00)	0.00 (0.00)	0.00 (0.00)	0.00 (0.00)	0.00 (0.00)	0.00 (0.00)	0.00 (0.00)	0.00 (0.00)
3	Tyr5	0.00 (0.00)	0.00 (0.00)	0.00 (0.00)	0.00 (0.00)	0.00 (0.00)	0.00 (0.00)	0.00 (0.00)	0.00 (0.00)	0.00 (0.00)	0.00 (0.00)
4	Leu 6	0.00 (0.00)	0.00 (0.00)	0.00 (0.00)	0.00 (0.00)	0.00 (0.00)	0.00 (0.00)	0.00 (0.00)	0.00 (0.00)	0.00 (0.00)	0.00 (0.00)
5	Asn 7	0.00 (0.00)	0.00 (0.00)	0.00 (0.00)	0.00 (0.00)	0.00 (0.00)	0.00 (0.00)	0.00 (0.00)	0.00 (0.00)	0.00 (0.00)	0.00 (0.00)
6	Val 8	0.00 (0.00)	0.00 (0.00)	0.00 (0.00)	0.00 (0.00)	0.00 (0.00)	0.00 (0.00)	0.00 (0.00)	0.00 (0.00)	0.00 (0.00)	0.00 (0.00)
7	Leu 9	0.00 (0.00)	0.00 (0.00)	0.00 (0.00)	0.00 (0.00)	0.00 (0.00)	0.00 (0.00)	0.00 (0.00)	0.00 (0.00)	0.00 (0.00)	0.00 (0.00)
8	Ala 10	0.00 (0.00)	0.00 (0.00)	0.00 (0.00)	0.00 (0.00)	0.00 (0.00)	0.00 (0.00)	0.00 (0.00)	0.00 (0.00)	0.00 (0.00)	0.00 (0.00)
9	Lys 11	0.00 (0.00)	0.00 (0.00)	0.00 (0.00)	0.00 (0.00)	0.00 (0.00)	0.00 (0.00)	0.00 (0.00)	0.00 (0.00)	0.00 (0.00)	0.00 (0.00)
10	Ala 12	0.00 (0.00)	0.00 (0.00)	0.00 (0.00)	0.00 (0.00)	0.00 (0.00)	0.00 (0.00)	0.00 (0.00)	0.00 (0.00)	0.00 (0.00)	0.00 (0.00)
11	Leu 13	0.00 (0.00)	0.00 (0.00)	0.00 (0.00)	0.00 (0.00)	0.00 (0.00)	0.00 (0.00)	0.00 (0.00)	0.00 (0.00)	0.00 (0.00)	0.00 (0.00)
12	Tyr 14	55.87 (0.87)	49.22 (0.72)	52.81 (0.81)	56.08 (0.87)	73.23 (1.10)	46.80 (0.71)	42.32 (0.65)	86.89 (0.88)	48.87 (0.73)	48.22 (0.76)
13	Asp 15	31.19 (-0.32) HS	23.11 (-0.31)	9.69 (-0.16)	10.84 (-0.18)	12.92 (-0.18)	3.43 (-0.03)	1.93 (-0.02)	1.99 (-0.02)	7.21 (-0.04)	12.31 (-0.07)
14	Asn 16	8.95 (-0.15)	7.61 (-0.13)	11.17 (-0.19)	8.47 (-0.14)	9.57 (-0.16)	6.88 (-0.12)	3.19 (-0.01)	7.61 (-0.13)	8.96 (-0.15)	12.41 (-0.21)
15	Val 17	3.80 (-0.04) H	1.74 (-0.02) H	2.33 (-0.03) H	4.44 (-0.05) H	9.16 (-0.10) H	4.71 (-0.05) H	8.54 (-0.10) H	3.55 (-0.04) H	4.32 (-0.05) H	2.60 (-0.03) H
16	Ala 18	0.00 (0.00)	0.00 (0.00)	0.00 (0.00)	0.00 (0.00)	0.00 (0.00)	0.00 (0.00)	0.00 (0.00)	0.00 (0.00)	0.00 (0.00)	0.00 (0.00)
17	Glu 19	38.66 (-0.29) HS	37.55 (-0.15) HS	38.06 (-0.16) HS	11.25 (0.16)	0.62 (0.01)	41.87 (-0.20) HS	16.23 (0.24)	18.09 (-0.20) HS	30.72 (-0.09) HS	41.14 (-0.18) HS
18	Ser 20	19.49 (-0.15) H	26.26 (-0.05)	24.28 (0.21)	17.86 (0.09) H	17.24 (0.18) H	20.65 (-0.19) H	30.23 (-0.09) H	23.58 (0.21)	28.79 (-0.02) H	23.42 (-0.19)
19	Pro 21	0.00 (0.00)	0.00 (0.00)	0.33 (0.01)	0.00 (0.00)	0.00 (0.00)	0.00 (0.00)	0.00 (0.00)	0.00 (0.00)	0.00 (0.00)	0.00 (0.00)
20	Asp 22	0.00 (0.00)	0.00 (0.00)	18.30 (-0.14) HS	24.80 (-0.28) HS	8.56 (0.09)	0.00 (0.00)	1.17 (0.02)	16.29 (0.01) HS	2.95 (0.02)	0.00 (0.00)
21	Glu 23	12.79 (0.13) HS	7.84 (0.14) HS	11.31 (0.16) HS	9.53 (-0.11) HS	25.82 (-0.18) HS	6.66 (0.11) HS	19.68 (0.01) HS	15.38 (-0.11) HS	18.84 (0.14) HS	9.08 (0.10) HS
22	Leu 24	0.00 (0.00)	0.00 (0.00)	0.00 (0.00)	0.00 (0.00)	0.00 (0.00)	0.00 (0.00)	0.00 (0.00)	0.00 (0.00)	0.00 (0.00)	0.00 (0.00)
23	Ser 25	0.00 (0.00)	0.00 (0.00)	0.00 (0.00)	0.00 (0.00)	0.00 (0.00)	0.00 (0.00)	0.00 (0.00)	0.00 (0.00)	0.00 (0.00)	0.00 (0.00)
24	Phe 26	0.00 (0.00)	0.00 (0.00)	0.00 (0.00)	0.00 (0.00)	0.00 (0.00)	0.00 (0.00)	0.00 (0.00)	0.00 (0.00)	0.00 (0.00)	0.00 (0.00)
25	Arg 27	0.00 (0.00)	0.00 (0.00)	0.00 (0.00)	0.00 (0.00)	0.00 (0.00)	0.00 (0.00)	0.00 (0.00)	0.00 (0.00)	0.00 (0.00)	0.00 (0.00)
26	Lys 28	2.34 (0.04)	13.64 (-0.16)	0.00 (0.00)	28.62 (-0.94)	26.08 (-0.50) HS	0.00 (0.00)	0.00 (0.00)	0.00 (0.00)	0.00 (0.00)	0.00 (0.00)
27	Gly 29	0.00 (0.00)	0.00 (0.00)	0.00 (0.00)	0.00 (0.00)	0.00 (0.00)	0.00 (0.00)	0.00 (0.00)	0.00 (0.00)	0.00 (0.00)	0.00 (0.00)
28	Asp 30	0.00 (0.00)	0.00 (0.00)	0.00 (0.00)	0.00 (0.00)	0.00 (0.00)	0.00 (0.00)	0.00 (0.00)	0.00 (0.00)	0.00 (0.00)	0.00 (0.00)
29	Ile 31	0.00 (0.00)	0.00 (0.00)	0.00 (0.00)	0.00 (0.00)	0.00 (0.00)	0.00 (0.00)	0.00 (0.00)	0.00 (0.00)	0.00 (0.00)	0.00 (0.00)
30	Met 32	0.00 (0.00)	0.00 (0.00)	0.00 (0.00)	0.00 (0.00)	0.00 (0.00)	0.00 (0.00)	0.00 (0.00)	0.00 (0.00)	0.00 (0.00)	0.00 (0.00)
31	Thr 33	0.00 (0.00)	0.00 (0.00)	0.00 (0.00)	0.00 (0.00)	0.00 (0.00)	0.00 (0.00)	0.00 (0.00)	0.00 (0.00)	0.00 (0.00)	0.00 (0.00)
32	Val 34	0.00 (0.00)	0.00 (0.00)	0.00 (0.00)	0.00 (0.00)	0.00 (0.00)	0.00 (0.00)	0.00 (0.00)	0.00 (0.00)	0.00 (0.00)	0.00 (0.00)
33	Leu 35	0.00 (0.00)	0.00 (0.00)	0.00 (0.00)	0.00 (0.00)	0.00 (0.00)	0.00 (0.00)	0.00 (0.00)	0.00 (0.00)	0.00 (0.00)	0.00 (0.00)
34	Glu 36	0.00 (0.00)	0.00 (0.00)	0.00 (0.00)	0.00 (0.00)	0.00 (0.00)	0.00 (0.00)	0.00 (0.00)	0.00 (0.00)	0.00 (0.00)	0.00 (0.00)
35	Arg 37	0.00 (0.00)	0.00 (0.00)	0.00 (0.00)	0.00 (0.00)	0.00 (0.00)	0.00 (0.00)	0.00 (0.00)	0.00 (0.00)	0.00 (0.00)	0.00 (0.00)
36	Asp 38	0.00 (0.00)	0.00 (0.00)	0.00 (0.00)	0.00 (0.00)	0.00 (0.00)	0.00 (0.00)	0.00 (0.00)	0.00 (0.00)	0.00 (0.00)	0.00 (0.00)
37	Thr 39	0.00 (0.00)	0.00 (0.00)	0.00 (0.00)	0.00 (0.00)	0.00 (0.00)	0.00 (0.00)	0.00 (0.00)	0.00 (0.00)	0.00 (0.00)	0.00 (0.00)
38	Gln 40	19.49 (-0.27)	0.00 (0.00)	0.00 (0.00)	22.41 (-0.21) H	0.00 (0.00)	0.00 (0.00)	10.12 (-0.12)	2.94 (-0.05)	0.00 (0.00)	0.00 (0.00)
39	Gly 41	4.91 (-0.03)	29.55 (-0.26)	6.90 (-0.07)	12.56 (-0.14)	13.11 (-0.15)	12.63 (-0.14)	24.89 (-0.12)	18.78 (-0.21) H	19.49 (-0.22) H	10.57 (-0.10)
40	Leu 42	59.98 (0.96)	42.35 (0.68)	56.65 (0.91)	67.19 (1.08)	41.35 (0.66)	47.04 (0.75)	52.97 (0.85)	50.06 (0.80)	54.05 (0.86)	48.11 (0.77)
41	Asp 43	19.78 (-0.10)	35.00 (0.01)	23.62 (-0.12)	34.85 (-0.17)	27.55 (-0.05)	36.87 (-0.02)	26.40 (-0.27)	45.77 (0.16)	46.69 (0.15)	40.54 (-0.14)
42	Gly 44	9.82 (0.11)	9.48 (0.10)	10.58 (0.13)	11.07 (0.14)	11.22 (0.11)	10.99 (0.13)	10.33 (0.13)	9.45 (0.10)	10.63 (0.13)	9.53 (0.11)
43	Trp 45	68.38 (0.42)	69.11 (0.42)	68.82 (0.42)	69.00 (0.44)	71.28 (0.44)	67.43 (0.41)	70.84 (0.45)	67.73 (0.40)	65.26 (0.37)	71.32 (0.46)
44	Trp 46	25.26 (0.00)	25.26 (0.00)	25.26 (0.00)	25.26 (0.00)	25.26 (0.00)	25.26 (0.00)	25.26 (0.00)	25.26 (0.00)	25.26 (0.00)	25.26 (0.00)
45	Leu 47	0.00 (0.00)	0.00 (0.00)	0.00 (0.00)	0.00 (0.00)	0.00 (0.00)	0.00 (0.00)	0.00 (0.00)	0.00 (0.00)	0.00 (0.00)	0.00 (0.00)
46	Cys 48	0.00 (0.00)	0.00 (0.00)	0.00 (0.00)	0.00 (0.00)	0.00 (0.00)	0.00 (0.00)	0.00 (0.00)	0.00 (0.00)	0.00 (0.00)	0.00 (0.00)
47	Ser 49	0.00 (0.00)	0.00 (0.00)	0.00 (0.00)	0.00 (0.00)	0.00 (0.00)	0.00 (0.00)	0.00 (0.00)	0.00 (0.00)	0.00 (0.00)	0.00 (0.00)
48	Leu 50	0.00 (0.00)	0.00 (0.00)	0.00 (0.00)	0.00 (0.00)	0.00 (0.00)	0.00 (0.00)	0.00 (0.00)	0.00 (0.00)	0.00 (0.00)	0.00 (0.00)
49	His 51	0.00 (0.00)	0.00 (0.00)	0.00 (0.00)	0.00 (0.00)	0.00 (0.00)	0.00 (0.00)	0.00 (0.00)	0.00 (0.00)	0.00 (0.00)	0.00 (0.00)
50	Gly 52	0.00 (0.00)	0.00 (0.00)	0.00 (0.00)	0.00 (0.00)	0.00 (0.00)	0.00 (0.00)	0.00 (0.00)	0.00 (0.00)	0.00 (0.00)	0.00 (0.00)
51	Arg 53	0.00 (0.00)	0.00 (0.00)	0.00 (0.00)	0.00 (0.00)	0.00 (0.00)	0.00 (0.00)	0.00 (0.00)	0.00 (0.00)	0.00 (0.00)	0.00 (0.00)
52	Gln 54	0.00 (0.00)	0.00 (0.00)	0.00 (0.00)	0.00 (0.00)	0.00 (0.00)	0.00 (0.00)	0.00 (0.00)	0.00 (0.00)	0.00 (0.00)	0.00 (0.00)
53	Gly 55	0.00 (0.00)	0.00 (0.00)	0.00 (0.00)	0.00 (0.00)	0.00 (0.00)	0.00 (0.00)	0.00 (0.00)	0.00 (0.00)	0.00 (0.00)	0.00 (0.00)
54	Ile 56	7.53 (0.12)	9.37 (0.15)	8.87 (0.14)	8.21 (0.13)	16.74 (0.27)	5.19 (0.08)	12.39 (0.20)	12.56 (0.20)	8.87 (0.14)	5.02 (0.08)
55	Val 57	0.00 (0.00)	0.00 (0.00)	0.00 (0.00)	0.00 (0.00)	0.00 (0.00)	0.00 (0.00)	0.00 (0.00)	0.00 (0.00)	0.00 (0.00)	0.00 (0.00)
56	Pro 58	20.93 (0.33)	15.24 (0.24)	16.07 (0.26)	17.92 (0.29)	18.09 (0.29)	17.58 (0.28)	23.10 (0.37)	20.43 (0.33)	21.60 (0.35)	22.27 (0.36)
57	Gly 59	0.14 (-0.00; -0.00)	0.00 (-0.00; 0.00)	0.00 (-0.00; 0.00)	0.00 (-0.00; 0.00)	0.00 (-0.00; 0.00)	0.14 (-0.00; -0.00)	0.00 (-0.00; 0.00)	0.00 (-0.00; 0.00)	0.15 (-0.00; -0.00)	0.14 (-0.00; -0.00)
58	Asn 60	48.79 (0.03)	59.79 (-0.15) H	60.98 (-0.28) H	64.12 (-0.41) H	63.42 (-0.54) H	63.91 (-0.32) H	67.65 (-0.15)	68.74 (-0.28) H	68.92 (-0.31) H	58.80 (-0.42) H
59	Arg 61	71.26 (-1.56) H	56.15 (-1.30) H	55.99 (-1.38) H	57.80 (-1.34) H	71.03 (-1.86) H	58.35 (-1.62) H	74.65 (-1.71) H	70.41 (-1.81) H	65.75 (-1.70) H	20.59 (0.33)
60	Leu 62	0.00 (0.00)	0.00 (0.00)	0.00 (0.00)	0.00 (0.00)	0.00 (0.00)	0.00 (0.00)	0.00 (0.00)	0.00 (0.00)	0.00 (0.00)	0.00 (0.00)
61	Lys 63	0.00 (0.00)	0.00 (0.00)	0.00 (0.00)	0.00 (0.00)	0.00 (0.00)	0.00 (0.00)	0.00 (0.00)	0.00 (0.00)	0.00 (0.00)	0.00 (0.00)
62	Ile 64	0.00 (0.00)	0.00 (0.00)	0.00 (0.00)	0.00 (0.00)	0.00 (0.00)	0.00 (0.00)	0.00 (0.00)	0.00 (0.00)	0.00 (0.00)	0.00 (0.00)
63	Leu 65	0.00 (0.00)	0.00 (0.00)	0.00 (0.00)	0.00 (0.00)	0.00 (0.00)	0.00 (0.00)	0.00 (0.00)	0.00 (0.00)	0.00 (0.00)	0.00 (0.00)
64	Val 66	0.00 (0.00)	0.00 (0.00)	0.00 (0.00)	0.00 (0.00)	0.00 (0.00)	0.00 (0.00)	0.00 (0.00)	0.00 (0.00)	0.00 (0.00)	0.00 (0.00)
65	Gly 67	0.00 (0.00)	0.00 (0.00)	0.00 (0.00)	0.00 (0.00)	0.00 (0.00)	0.00 (0.00)	0.00 (0.00)	0.00 (0.00)	0.00 (0.00)	0.00 (0.00)
66	Met 68	0.00 (0.00)	0.00 (0.00)	0.00 (0.00)	0.00 (0.00)	0.00 (0.00)	0.00 (0.00)	0.00 (0.00)	0.00 (0.00)	0.00 (0.00)	0.00 (0.00)
67	Tyr 69	0.00 (0.00)	0.00 (0.00)	0.00 (0.00)	0.00 (0.00)	0.00 (0.00)	0.00 (0.00)	0.00 (0.00)	0.00 (0.00)	0.00 (0.00)	0.00 (0.00)
68	Asp 70	0.00 (0.00)	0.00 (0.00)	0.00 (0.00)	0.00 (0.00)	0.00 (0.00)	0.00 (0.00)	0.00 (0.00)	0.00 (0.00)	0.00 (0.00)	0.00 (0.00)
69	Lys 71	0.00 (0.00)	0.00 (0.00)	0.00 (0.00)	0.00 (0.00)	0.00 (0.00)	0.00 (0.00)	0.00 (0.00)	0.00 (0.00)	0.00 (0.00)	0.00 (0.00)
70	Lys 72	0.00 (0.00)	0.00 (0.00)	0.00 (0.00)	0.00 (0.00)	0.00 (0.00)	0.00 (0.00)	0.00 (0.00)	0.00 (0.00)	0.00 (0.00)	0.00 (0.00)
71	Pro 73	0.00 (0.00)	0.00 (0.00)	0.00 (0.00)	0.00 (0.00)	0.00 (0.00)	0.00 (0.00)	0.00 (0.00)	0.00 (0.00)	0.00 (0.00)	0.00 (0.00)
72	Ala 74	0.00 (0.00)	0.00 (0.00)	0.00 (0.00)	0.00 (0.00)	0.00 (0.00)	0.00 (0.00)	0.00 (0.00)	0.00 (0.00)	0.00 (0.00)	0.00 (0.00)

UNIPROT	Interface residue	11_PEST	12_PEST	13_PEST	14_PEST	15_PEST	16_PEST	17_PEST	18_PEST	19_PEST	20_PEST
1	Met 3	0.00 (0.00)	0.00 (0.00)	0.00 (0.00)	0.00 (0.00)	0.00 (0.00)	0.00 (0.00)	0.00 (0.00)	0.00 (0.00)	0.00 (0.00)	0.00 (0.00)
2	LYS 4	0.00 (0.00)	0.00 (0.00)	0.00 (0.00)	0.00 (0.00)	0.00 (0.00)	0.00 (0.00)	0.00 (0.00)	0.00 (0.00)	0.00 (0.00)	0.00 (0.00)
3	Tyr5	0.00 (0.00)	0.00 (0.00)	0.00 (0.00)	0.00 (0.00)	0.00 (0.00)	0.00 (0.00)	0.00 (0.00)	0.00 (0.00)	0.00 (0.00)	0.00 (0.00)
4	Leu 6	0.00 (0.00)	0.00 (0.00)	0.00 (0.00)	0.00 (0.00)	0.00 (0.00)	0.00 (0.00)	0.00 (0.00)	0.00 (0.00)	0.00 (0.00)	0.00 (0.00)
5	Asn 7	0.00 (0.00)	0.00 (0.00)	0.00 (0.00)	0.00 (0.00)	0.00 (0.00)	0.00 (0.00)	0.00 (0.00)	0.00 (0.00)	0.00 (0.00)	0.00 (0.00)
6	Val 8	0.00 (0.00)	0.00 (0.00)	0.00 (0.00)	0.00 (0.00)	0.00 (0.00)	0.00 (0.00)	0.00 (0.00)	0.00 (0.00)	0.00 (0.00)	0.00 (0.00)
7	Leu 9	0.00 (0.00)	0.00 (0.00)	0.00 (0.00)	0.00 (0.00)	0.00 (0.00)	0.00 (0.00)	0.00 (0.00)	0.00 (0.00)	0.00 (0.00)	0.00 (0.00)
8	Ala 10	0.00 (0.00)	0.00 (0.00)	0.00 (0.00)	0.00 (0.00)	0.00 (0.00)	0.00 (0.00)	0.00 (0.00)	0.00 (0.00)	0.00 (0.00)	0.00 (0.00)
9	Lys 11	0.00 (0.00)	0.00 (0.00)	0.00 (0.00)	0.00 (0.00)	0.00 (0.00)	0.00 (0.00)	0.00 (0.00)	0.00 (0.00)	0.00 (0.00)	0.00 (0.00)
10	Ala 12	0.00 (0.00)	0.00 (0.00)	0.00 (0.00)	0.00 (0.00)	0.00 (0.00)	0.00 (0.00)	0.00 (0.00)	0.00 (0.00)	0.00 (0.00)	0.00 (0.00)
11	Leu 13	0.00 (0.00)	0.00 (0.00)	0.00 (0.00)	0.00 (0.00)	0.00 (0.00)	0.00 (0.00)	0.00 (0.00)	0.00 (0.00)	0.00 (0.00)	0.00 (0.00)
12	Tyr 14	90.27 (0.76)	53.08 (0.81)	44.96 (0.69)	56.97 (0.76)	67.78 (0.99)	44.68 (0.66)	65.60 (0.73)	69.39 (1.00)	63.74 (0.91)	64.20 (0.97)
13	Asp 15	3.11 (-0.03)	8.96 (-0.19)	4.80 (-0.09)	5.38 (-0.00)	2.38 (-0.03)	10.93 (-0.16)	1.84 (-0.02)	4.05 (-0.07)	18.56 (-0.18)	23.72 (0.17)
14	Asn 16	7.49 (-0.13)	9.21 (-0.16)	9.81 (-0.17)	2.92 (-0.00)	7.00 (-0.12)	7.49 (-0.13)	12.51 (-0.21)	8.72 (-0.15)	3.23 (-0.03)	1.90 (-0.02)
15	Val 17	2.08 (-0.02) H	3.96 (-0.05) H	2.93 (-0.03) H	10.82 (-0.12) H	6.25 (-0.07) H	3.31 (-0.04) H	6.77 (-0.08) H	5.93 (-0.07) H	10.81 (-0.12) H	10.46 (-0.11) H
16	Ala 18	0.00 (0.00)	0.00 (0.00)	0.00 (0.00)	0.00 (0.00)	0.00 (0.00)	0.00 (0.00)	0.00 (0.00)	0.00 (0.00)	0.00 (0.00)	0.00 (0.00)
17	Glu 19	26.37 (-0.26) HS	25.96 (-0.15) HS	20.30 (-0.16) HS	13.34 (0.21)	18.93 (0.27)	41.05 (-0.32) HS	4.55 (0.07)	10.94 (0.17)	11.14 (-0.07) S	27.73 (-0.10)
18	Ser 20	28.64 (0.04)	14.60 (-0.03) H	22.05 (0.24)	28.27 (-0.11) H	25.64 (-0.05) H	17.27 (-0.17)	27.53 (-0.08) H	24.07 (0.24)	36.95 (-0.10)	13.88 (0.06)
19	Pro 21	0.33 (0.01)	0.00 (0.00)	0.00 (0.00)	0.00 (0.00)	0.00 (0.00)	0.00 (0.00)	0.00 (0.00)	0.00 (0.00)	1.34 (0.02)	0.00 (0.00)
20	Asp 22	18.14 (-0.09) HS	0.00 (0.00)	13.27 (-0.10) HS	0.00 (0.00)	0.00 (0.00)	5.03 (-0.06)	2.68 (0.04)	17.22 (-0.05) HS	5.90 (0.08)	0.00 (0.00)
21	Glu 23	8.78 (-0.12) HS	14.56 (0.10) HS	12.24 (-0.12) HS	22.27 (-0.02) HS	17.01 (0.05) HS	4.90 (-0.04) HS	24.69 (-0.10) HS	12.10 (-0.13) HS	15.84 (-0.16) HS	24.31 (-0.09) HS
22	Leu 24	0.00 (0.00)	0.00 (0.00)	0.00 (0.00)	0.00 (0.00)	0.00 (0.00)	0.00 (0.00)	0.00 (0.00)	0.00 (0.00)	0.00 (0.00)	0.00 (0.00)
23	Ser 25	0.00 (0.00)	0.00 (0.00)	0.00 (0.00)	0.00 (0.00)	0.00 (0.00)	0.00 (0.00)	0.00 (0.00)	0.00 (0.00)	0.00 (0.00)	0.00 (0.00)
24	Phe 26	0.00 (0.00)	0.00 (0.00)	0.00 (0.00)	0.00 (0.00)	0.00 (0.00)	0.00 (0.00)	0.00 (0.00)	0.00 (0.00)	0.00 (0.00)	0.00 (0.00)
25	Arg 27	0.00 (0.00)	0.00 (0.00)	0.00 (0.00)	0.00 (0.00)	0.00 (0.00)	0.00 (0.00)	0.00 (0.00)	0.00 (0.00)	0.00 (0.00)	0.00 (0.00)
26	Lys 28	9.34 (-0.35)	4.21 (-0.16)	0.00 (0.00)	0.00 (0.00)	8.16 (-0.30)	0.00 (0.00)	0.00 (0.00)	31.69 (-0.07) HS	0.13 (-0.00)	13.07 (-0.24)
27	Gly 29	0.00 (0.00)	0.00 (0.00)	0.00 (0.00)	0.00 (0.00)	0.00 (0.00)	0.00 (0.00)	0.00 (0.00)	0.00 (0.00)	0.00 (0.00)	0.00 (0.00)
28	Asp 30	0.00 (0.00)	0.00 (0.00)	0.00 (0.00)	0.00 (0.00)	0.00 (0.00)	0.00 (0.00)	0.00 (0.00)	0.00 (0.00)	0.00 (0.00)	0.00 (0.00)
29	Ile 31	0.00 (0.00)	0.00 (0.00)	0.00 (0.00)	0.00 (0.00)	0.00 (0.00)	0.00 (0.00)	0.00 (0.00)	0.00 (0.00)	0.00 (0.00)	0.00 (0.00)
30	Met 32	0.00 (0.00)	0.00 (0.00)	0.00 (0.00)	0.00 (0.00)	0.00 (0.00)	0.00 (0.00)	0.00 (0.00)	0.00 (0.00)	0.00 (0.00)	0.00 (0.00)
31	Thr 33	0.00 (0.00)	0.00 (0.00)	0.00 (0.00)	0.00 (0.00)	0.00 (0.00)	0.00 (0.00)	0.00 (0.00)	0.00 (0.00)	0.00 (0.00)	0.00 (0.00)
32	Val 34	0.00 (0.00)	0.00 (0.00)	0.00 (0.00)	0.00 (0.00)	0.00 (0.00)	0.00 (0.00)	0.00 (0.00)	0.00 (0.00)	0.00 (0.00)	0.00 (0.00)
33	Leu 35	0.00 (0.00)	0.00 (0.00)	0.00 (0.00)	0.00 (0.00)	0.00 (0.00)	0.00 (0.00)	0.00 (0.00)	0.00 (0.00)	0.00 (0.00)	0.00 (0.00)
34	Glu 36	0.00 (0.00)	0.00 (0.00)	0.00 (0.00)	0.00 (0.00)	0.00 (0.00)	0.00 (0.00)	0.00 (0.00)	0.00 (0.00)	0.00 (0.00)	0.00 (0.00)
35	Arg 37	0.00 (0.00)	0.00 (0.00)	0.00 (0.00)	0.00 (0.00)	0.00 (0.00)	0.00 (0.00)	0.00 (0.00)	0.00 (0.00)	0.00 (0.00)	0.00 (0.00)
36	Asp 38	0.00 (0.00)	0.00 (0.00)	0.00 (0.00)	0.00 (0.00)	0.00 (0.00)	0.00 (0.00)	0.00 (0.00)	0.00 (0.00)	0.00 (0.00)	0.00 (0.00)
37	Thr 39	0.00 (0.00)	0.00 (0.00)	0.00 (0.00)	0.00 (0.00)	0.00 (0.00)	0.00 (0.00)	0.00 (0.00)	0.00 (0.00)	0.00 (0.00)	0.00 (0.00)
38	Gln 40	3.07 (-0.05)	0.12 (-0.00)	3.68 (-0.06)	0.00 (0.00)	0.00 (0.00)	1.72 (-0.02)	2.56 (-0.03)	0.00 (0.00)	0.00 (0.00)	0.49 (-0.01)
39	Gly 41	16.31 (-0.19) H	13.75 (-0.16)	32.57 (-0.10)	19.24 (-0.22)	30.66 (-0.30) H	5.55 (-0.06)	28.22 (-0.15)	11.98 (-0.13)	12.88 (-0.15)	14.43 (-0.14)
40	Leu 42	48.71 (0.78)	51.78 (0.83)	49.21 (0.79)	38.00 (0.61)	55.55 (0.89)	50.61 (0.81)	55.83 (0.89)	55.63 (0.89)	37.99 (0.61)	48.60 (0.78)
41	Asp 43	37.40 (0.05)	30.47 (-0.12)	51.84 (-0.39) S	40.55 (0.16)	36.85 (0.05)	21.00 (-0.13)	66.99 (-0.02) HS	32.55 (-0.18)	55.15 (-0.01) HS	32.04 (-0.21)
42	Gly 44	10.90 (0.10)	10.05 (0.12)	10.66 (0.12)	10.53 (0.12)	10.55 (0.11)	10.61 (0.11)	10.40 (0.11)	10.66 (0.12)	11.00 (0.12)	10.42 (0.13)
43	Trp 45	70.42 (0.46)	70.17 (0.42)	68.71 (0.44)	71.92 (0.46)	66.52 (0.39)	69.61 (0.43)	71.01 (0.45)	71.75 (0.51)	72.69 (0.52)	72.45 (0.49)
44	Trp 46	25.26 (0.00)	25.26 (0.00)	25.26 (0.00)	25.26 (0.00)	25.26 (0.00)	25.26 (0.00)	25.26 (0.00)	25.26 (0.00)	25.26 (0.00)	25.26 (0.00)
45	Leu 47	0.00 (0.00)	0.00 (0.00)	0.00 (0.00)	0.00 (0.00)	0.00 (0.00)	0.00 (0.00)	0.00 (0.00)	0.00 (0.00)	0.00 (0.00)	0.00 (0.00)
46	Cys 48	0.00 (0.00)	0.00 (0.00)	0.00 (0.00)	0.00 (0.00)	0.00 (0.00)	0.00 (0.00)	0.00 (0.00)	0.00 (0.00)	0.00 (0.00)	0.00 (0.00)
47	Ser 49	0.00 (0.00)	0.00 (0.00)	0.00 (0.00)	0.00 (0.00)	0.00 (0.00)	0.00 (0.00)	0.00 (0.00)	0.00 (0.00)	0.00 (0.00)	0.00 (0.00)
48	Leu 50	0.00 (0.00)	0.00 (0.00)	0.00 (0.00)	0.00 (0.00)	0.00 (0.00)	0.00 (0.00)	0.00 (0.00)	0.00 (0.00)	0.00 (0.00)	0.00 (0.00)
49	His 51	0.00 (0.00)	0.00 (0.00)	0.00 (0.00)	0.00 (0.00)	0.00 (0.00)	0.00 (0.00)	0.00 (0.00)	0.00 (0.00)	0.00 (0.00)	0.00 (0.00)
50	Gly 52	0.00 (0.00)	0.00 (0.00)	0.00 (0.00)	0.00 (0.00)	0.00 (0.00)	0.00 (0.00)	0.00 (0.00)	0.00 (0.00)	0.00 (0.00)	0.00 (0.00)
51	Arg 53	0.00 (0.00)	0.00 (0.00)	0.00 (0.00)	0.00 (0.00)	0.00 (0.00)	0.00 (0.00)	0.00 (0.00)	0.00 (0.00)	0.00 (0.00)	0.00 (0.00)
52	Gln 54	0.00 (0.00)	0.00 (0.00)	0.00 (0.00)	0.00 (0.00)	0.00 (0.00)	0.00 (0.00)	0.00 (0.00)	0.00 (0.00)	0.00 (0.00)	0.00 (0.00)
53	Gly 55	0.00 (0.00)	0.00 (0.00)	0.00 (0.00)	0.00 (0.00)	0.00 (0.00)	0.00 (0.00)	0.00 (0.00)	0.00 (0.00)	0.00 (0.00)	0.00 (0.00)
54	Ile 56	11.55 (0.18)	10.04 (0.16)	11.22 (0.18)	8.03 (0.13)	9.88 (0.16)	6.36 (0.10)	14.56 (0.23)	13.56 (0.22)	12.06 (0.19)	7.87 (0.13)
55	Val 57	0.00 (0.00)	0.00 (0.00)	0.00 (0.00)	0.00 (0.00)	0.00 (0.00)	0.00 (0.00)	0.00 (0.00)	0.00 (0.00)	0.00 (0.00)	0.00 (0.00)
56	Pro 58	23.10 (0.37)	16.24 (0.26)	22.43 (0.36)	22.94 (0.37)	21.11 (0.34)	22.27 (0.36)	21.76 (0.35)	21.27 (0.34)	26.44 (0.42)	21.27 (0.34)
57	Gly 59	0.00 (-0.00; 0.00)	0.29 (-0.00; -0.00)	0.29 (-0.00; -0.00)	0.14 (-0.00; -0.00)	0.14 (-0.00; -0.00)	0.29 (-0.00; -0.00)	0.00 (-0.00; 0.00)	0.00 (-0.00; 0.00)	0.00 (-0.00; 0.00)	0.00 (-0.00; 0.00)
58	Asn 60	65.06 (-0.30) H	66.70 (-0.24) H	60.36 (0.01)	59.40 (-0.03)	58.80 (0.03)	65.28 (-0.27) H	61.88 (-0.32) H	71.57 (-0.34) H	60.48 (-0.19) H	62.60 (-0.27) H
59	Arg 61	66.41 (-1.75) H	55.81 (-1.35) H	69.23 (-1.68) H	73.96 (-1.84) H	67.68 (-1.80) H	69.84 (-1.78) H	48.15 (-0.36)	66.19 (-1.73) H	72.19 (-1.72) H	76.43 (-1.90) H
60	Leu 62	0.00 (0.00)	0.00 (0.00)	0.00 (0.00)	0.00 (0.00)	0.00 (0.00)	0.00 (0.00)	0.00 (0.00)	0.00 (0.00)	0.00 (0.00)	0.00 (0.00)
61	Lys 63	0.00 (0.00)	0.00 (0.00)	0.00 (0.00)	0.00 (0.00)	0.00 (0.00)	0.00 (0.00)	0.00 (0.00)	0.00 (0.00)	0.00 (0.00)	0.00 (0.00)
62	Ile 64	0.00 (0.00)	0.00 (0.00)	0.00 (0.00)	0.00 (0.00)	0.00 (0.00)	0.00 (0.00)	0.00 (0.00)	0.00 (0.00)	0.00 (0.00)	0.00 (0.00)
63	Leu 65	0.00 (0.00)	0.00 (0.00)	0.00 (0.00)	0.00 (0.00)	0.00 (0.00)	0.00 (0.00)	0.00 (0.00)	0.00 (0.00)	0.00 (0.00)	0.00 (0.00)
64	Val 66	0.00 (0.00)	0.00 (0.00)	0.00 (0.00)	0.00 (0.00)	0.00 (0.00)	0.00 (0.00)	0.00 (0.00)	0.00 (0.00)	0.00 (0.00)	0.00 (0.00)
65	Gly 67	0.00 (0.00)	0.00 (0.00)	0.00 (0.00)	0.00 (0.00)	0.00 (0.00)	0.00 (0.00)	0.00 (0.00)	0.00 (0.00)	0.00 (0.00)	0.00 (0.00)
66	Met 68	0.00 (0.00)	0.00 (0.00)	0.00 (0.00)	0.00 (0.00)	0.00 (0.00)	0.00 (0.00)	0.00 (0.00)	0.00 (0.00)	0.00 (0.00)	0.00 (0.00)
67	Tyr 69	0.00 (0.00)	0.00 (0.00)	0.00 (0.00)	0.00 (0.00)	0.00 (0.00)	0.00 (0.00)	0.00 (0.00)	0.00 (0.00)	0.00 (0.00)	0.00 (0.00)
68	Asp 70	0.00 (0.00)	0.00 (0.00)	0.00 (0.00)	0.00 (0.00)	0.00 (0.00)	0.00 (0.00)	0.00 (0.00)	0.00 (0.00)	0.00 (0.00)	0.00 (0.00)
69	Lys 71	0.00 (0.00)	0.00 (0.00)	0.00 (0.00)	0.00 (0.00)	0.00 (0.00)	0.00 (0.00)	0.00 (0.00)	0.00 (0.00)	0.00 (0.00)	0.00 (0.00)
70	Lys 72	0.00 (0.00)	0.00 (0.00)	0.00 (0.00)	0.00 (0.00)	0.00 (0.00)	0.00 (0.00)	0.00 (0.00)	0.00 (0.00)	0.00 (0.00)	0.00 (0.00)
71	Pro 73	0.00 (0.00)	0.00 (0.00)	0.00 (0.00)	0.00 (0.00)	0.00 (0.00)	0.00 (0.00)	0.00 (0.00)	0.00 (0.00)	0.00 (0.00)	0.00 (0.00)
72	Ala 74	0.00 (0.00)	0.00 (0.00)	0.00 (0.00)	0.00 (0.00)	0.00 (0.00)	0.00 (0.00)	0.00 (0.00)	0.00 (0.00)	0.00 (0.00)	0.00 (0.00)

UNIPROT	Interface residue	21_PEST	22_PEST	23_PEST	24_PEST	25_PEST	26_PEST	27_PEST	28_PEST	29_PEST	30_PEST
1	Met 3	0.00 (0.00)	0.00 (0.00)	0.00 (0.00)	0.00 (0.00)	0.00 (0.00)	0.00 (0.00)	0.00 (0.00)	0.00 (0.00)	0.00 (0.00)	0.00 (0.00)
2	LYS 4	0.00 (0.00)	0.00 (0.00)	0.00 (0.00)	0.00 (0.00)	0.00 (0.00)	0.00 (0.00)	0.00 (0.00)	0.00 (0.00)	0.00 (0.00)	0.00 (0.00)
3	Tyr5	0.00 (0.00)	0.00 (0.00)	0.00 (0.00)	0.00 (0.00)	0.00 (0.00)	0.00 (0.00)	0.00 (0.00)	0.00 (0.00)	0.00 (0.00)	0.00 (0.00)
4	Leu 6	0.00 (0.00)	0.00 (0.00)	0.00 (0.00)	0.00 (0.00)	0.00 (0.00)	0.00 (0.00)	0.00 (0.00)	0.00 (0.00)	0.00 (0.00)	0.00 (0.00)
5	Asn 7	0.00 (0.00)	0.00 (0.00)	0.00 (0.00)	0.00 (0.00)	0.00 (0.00)	0.00 (0.00)	0.00 (0.00)	0.00 (0.00)	0.00 (0.00)	0.00 (0.00)
6	Val 8	0.00 (0.00)	0.00 (0.00)	0.00 (0.00)	0.00 (0.00)	0.00 (0.00)	0.00 (0.00)	0.00 (0.00)	0.00 (0.00)	0.00 (0.00)	0.00 (0.00)
7	Leu 9	0.00 (0.00)	0.00 (0.00)	0.00 (0.00)	0.00 (0.00)	0.00 (0.00)	0.00 (0.00)	0.00 (0.00)	0.00 (0.00)	0.00 (0.00)	0.00 (0.00)
8	Ala 10	0.00 (0.00)	0.00 (0.00)	0.00 (0.00)	0.00 (0.00)	0.00 (0.00)	0.00 (0.00)	0.00 (0.00)	0.00 (0.00)	0.00 (0.00)	0.00 (0.00)
9	Lys 11	0.00 (0.00)	0.00 (0.00)	0.00 (0.00)	0.00 (0.00)	0.00 (0.00)	0.00 (0.00)	0.00 (0.00)	0.00 (0.00)	0.00 (0.00)	0.00 (0.00)
10	Ala 12	0.00 (0.00)	0.00 (0.00)	0.00 (0.00)	0.00 (0.00)	0.00 (0.00)	0.00 (0.00)	0.00 (0.00)	0.00 (0.00)	0.00 (0.00)	0.00 (0.00)
11	Leu 13	0.00 (0.00)	0.00 (0.00)	0.00 (0.00)	0.00 (0.00)	0.00 (0.00)	0.00 (0.00)	0.00 (0.00)	0.00 (0.00)	0.00 (0.00)	0.00 (0.00)
12	Tyr 14	44.08 (0.69)	67.29 (1.04)	73.50 (1.04)	51.83 (0.75)	62.48 (0.97)	84.26 (0.95) H	48.79 (0.72)	75.85 (1.18)	74.87 (1.12)	75.72 (1.15)
13	Asp 15	48.93 (0.17)	24.20 (-0.24)	26.97 (-0.27)	0.00 (0.00)	31.43 (-0.30)	15.10 (-0.06)	16.26 (-0.03)	49.97 (-0.39) HS	20.57 (-0.26)	16.45 (-0.27)
14	Asn 16	4.50 (-0.05)	5.90 (-0.10)	1.97 (0.02)	21.84 (-0.37)	8.23 (-0.14)	17.72 (-0.29) H	7.00 (-0.12)	6.01 (-0.10)	7.99 (-0.14)	9.09 (-0.15)
15	Val 17	36.44 (0.28) H	4.66 (-0.05) H	8.00 (-0.09) H	0.98 (-0.01)	3.19 (-0.04) H	7.85 (-0.07) H	8.11 (-0.07) H	5.39 (0.03) H	1.10 (-0.01) H	5.48 (-0.06) H
16	Ala 18	0.00 (0.00)	0.00 (0.00)	0.00 (0.00)	0.00 (0.00)	0.00 (0.00)	0.00 (0.00)	0.00 (0.00)	0.00 (0.00)	0.00 (0.00)	0.00 (0.00)
17	Glu 19	8.39 (-0.09)	22.30 (-0.18) HS	26.98 (0.42)	0.00 (0.00)	33.12 (-0.15) HS	33.06 (-0.12) S	17.89 (0.26)	20.65 (-0.22) HS	22.27 (-0.24) HS	33.14 (-0.12) HS
18	Ser 20	7.96 (-0.07)	23.35 (-0.06) H	17.57 (-0.07) H	33.13 (0.11)	17.03 (-0.02) H	13.55 (0.22)	17.73 (0.28)	20.77 (0.09) H	33.69 (-0.11)	15.56 (0.25)
19	Pro 21	0.00 (0.00)	0.00 (0.00)	0.00 (0.00)	0.00 (0.00)	0.00 (0.00)	0.00 (0.00)	0.00 (0.00)	0.00 (0.00)	1.17 (0.02)	0.00 (0.00)
20	Asp 22	0.00 (0.00)	14.98 (-0.06) HS	0.00 (0.00)	10.43 (-0.12) HS	9.26 (0.14)	0.00 (0.00)	0.00 (0.00)	10.42 (0.02) HS	17.88 (-0.08) HS	0.00 (0.00)
21	Glu 23	23.40 (-0.24) HS	10.23 (-0.09) HS	18.25 (-0.02) HS	8.61 (-0.14)	11.52 (0.14) HS	11.86 (0.07) HS	11.44 (-0.16) HS	7.97 (-0.08) HS	10.03 (-0.08) HS	17.22 (0.06) HS
22	Leu 24	0.00 (0.00)	0.00 (0.00)	0.00 (0.00)	0.00 (0.00)	0.00 (0.00)	0.00 (0.00)	0.00 (0.00)	0.00 (0.00)	0.00 (0.00)	0.00 (0.00)
23	Ser 25	0.00 (0.00)	0.00 (0.00)	0.00 (0.00)	0.00 (0.00)	0.00 (0.00)	0.00 (0.00)	0.00 (0.00)	0.00 (0.00)	0.00 (0.00)	0.00 (0.00)
24	Phe 26	0.00 (0.00)	0.00 (0.00)	0.00 (0.00)	0.00 (0.00)	0.00 (0.00)	0.00 (0.00)	0.00 (0.00)	0.00 (0.00)	0.00 (0.00)	0.00 (0.00)
25	Arg 27	61.87 (-1.34) HS	0.00 (0.00)	19.64 (-0.56)	0.00 (0.00)	50.88 (-1.17) HS	0.00 (0.00)	0.00 (0.00)	15.40 (-0.04)	0.29 (-0.01)	0.00 (0.00)
26	Lys 28	13.43 (0.03)	18.21 (-0.24) HS	20.26 (-0.46) HS	2.77 (-0.10)	38.20 (-0.26) HS	7.61 (-0.28)	1.97 (-0.07)	53.12 (-0.39) H	39.10 (0.33) HS	46.68 (-1.15) HS
27	Gly 29	0.00 (0.00)	0.00 (0.00)	0.00 (0.00)	0.00 (0.00)	0.00 (0.00)	0.00 (0.00)	0.00 (0.00)	0.00 (0.00)	0.00 (0.00)	0.00 (0.00)
28	Asp 30	0.00 (0.00)	0.00 (0.00)	0.00 (0.00)	0.00 (0.00)	0.00 (0.00)	0.00 (0.00)	0.00 (0.00)	0.00 (0.00)	0.00 (0.00)	0.00 (0.00)
29	Ile 31	0.00 (0.00)	0.00 (0.00)	0.00 (0.00)	0.00 (0.00)	0.00 (0.00)	0.00 (0.00)	0.00 (0.00)	0.00 (0.00)	0.00 (0.00)	0.00 (0.00)
30	Met 32	0.00 (0.00)	0.00 (0.00)	0.00 (0.00)	0.00 (0.00)	0.00 (0.00)	0.00 (0.00)	0.00 (0.00)	0.00 (0.00)	0.00 (0.00)	0.00 (0.00)
31	Thr 33	0.00 (0.00)	0.00 (0.00)	0.00 (0.00)	0.00 (0.00)	0.00 (0.00)	0.00 (0.00)	0.00 (0.00)	0.00 (0.00)	0.00 (0.00)	0.00 (0.00)
32	Val 34	0.00 (0.00)	0.00 (0.00)	0.00 (0.00)	0.00 (0.00)	0.00 (0.00)	0.00 (0.00)	0.00 (0.00)	0.00 (0.00)	0.00 (0.00)	0.00 (0.00)
33	Leu 35	0.00 (0.00)	0.00 (0.00)	0.00 (0.00)	0.00 (0.00)	0.00 (0.00)	0.00 (0.00)	0.00 (0.00)	0.00 (0.00)	0.00 (0.00)	0.00 (0.00)
34	Glu 36	0.00 (0.00)	0.00 (0.00)	0.00 (0.00)	0.00 (0.00)	0.00 (0.00)	0.00 (0.00)	0.00 (0.00)	0.00 (0.00)	0.00 (0.00)	0.00 (0.00)
35	Arg 37	0.00 (0.00)	0.00 (0.00)	0.00 (0.00)	0.00 (0.00)	0.00 (0.00)	0.00 (0.00)	0.00 (0.00)	0.00 (0.00)	0.00 (0.00)	0.00 (0.00)
36	Asp 38	0.00 (0.00)	0.00 (0.00)	0.00 (0.00)	0.00 (0.00)	0.00 (0.00)	0.00 (0.00)	0.00 (0.00)	0.00 (0.00)	0.00 (0.00)	0.00 (0.00)
37	Thr 39	0.00 (0.00)	0.00 (0.00)	0.00 (0.00)	0.00 (0.00)	0.00 (0.00)	0.00 (0.00)	0.00 (0.00)	0.00 (0.00)	0.00 (0.00)	0.00 (0.00)
38	Gln 40	4.64 (-0.05)	1.11 (-0.01)	3.68 (-0.04)	0.00 (0.00)	13.27 (-0.19) H	0.00 (0.00)	0.00 (0.00)	5.77 (-0.10)	4.91 (-0.08) H	23.03 (-0.10)
39	Gly 41	32.34 (-0.15) H	35.82 (-0.21) H	7.29 (-0.06)	12.07 (-0.13)	10.95 (-0.12)	5.80 (-0.06)	0.49 (-0.01)	12.74 (-0.13)	9.20 (-0.11)	41.89 (0.09)
40	Leu 42	44.09 (0.71)	45.36 (0.73)	52.79 (0.84)	52.13 (0.83)	66.35 (1.06)	51.65 (0.83)	51.45 (0.82)	53.14 (0.85)	54.66 (0.87)	50.28 (0.80)
41	Asp 43	32.72 (-0.08)	55.82 (0.22)	22.61 (-0.13)	31.45 (-0.15)	21.73 (-0.13)	52.71 (0.12)	22.10 (-0.07)	19.42 (-0.18)	26.57 (-0.17)	45.96 (0.06)
42	Gly 44	10.86 (0.12)	11.38 (0.14)	10.46 (0.12)	10.47 (0.12)	10.72 (0.13)	10.11 (0.11)	10.51 (0.12)	11.04 (0.15)	11.28 (0.13)	10.39 (0.14)
43	Trp 45	71.82 (0.47)	69.42 (0.42)	69.63 (0.44)	69.91 (0.43)	69.79 (0.45)	68.41 (0.43)	71.42 (0.44)	68.51 (0.42)	69.61 (0.44)	73.42 (0.51)
44	Trp 46	25.26 (0.00)	25.26 (0.00)	25.26 (0.00)	25.26 (0.00)	25.26 (0.00)	25.26 (0.00)	25.26 (0.00)	25.26 (0.00)	25.26 (0.00)	25.26 (0.00)
45	Leu 47	0.00 (0.00)	0.00 (0.00)	0.00 (0.00)	0.00 (0.00)	0.00 (0.00)	0.00 (0.00)	0.00 (0.00)	0.00 (0.00)	0.00 (0.00)	0.00 (0.00)
46	Cys 48	0.00 (0.00)	0.00 (0.00)	0.00 (0.00)	0.00 (0.00)	0.00 (0.00)	0.00 (0.00)	0.00 (0.00)	0.00 (0.00)	0.00 (0.00)	0.00 (0.00)
47	Ser 49	0.00 (0.00)	0.00 (0.00)	0.00 (0.00)	0.00 (0.00)	0.00 (0.00)	0.00 (0.00)	0.00 (0.00)	0.00 (0.00)	0.00 (0.00)	0.00 (0.00)
48	Leu 50	0.00 (0.00)	0.00 (0.00)	0.00 (0.00)	0.00 (0.00)	0.00 (0.00)	0.00 (0.00)	0.00 (0.00)	0.00 (0.00)	0.00 (0.00)	0.00 (0.00)
49	His 51	0.00 (0.00)	0.00 (0.00)	0.00 (0.00)	0.00 (0.00)	0.00 (0.00)	0.00 (0.00)	0.00 (0.00)	0.00 (0.00)	0.00 (0.00)	0.00 (0.00)
50	Gly 52	0.00 (0.00)	0.00 (0.00)	0.00 (0.00)	0.00 (0.00)	0.00 (0.00)	0.00 (0.00)	0.00 (0.00)	0.00 (0.00)	0.00 (0.00)	0.00 (0.00)
51	Arg 53	0.00 (0.00)	0.00 (0.00)	0.00 (0.00)	0.00 (0.00)	0.00 (0.00)	0.00 (0.00)	0.00 (0.00)	0.00 (0.00)	0.00 (0.00)	0.00 (0.00)
52	Gln 54	0.00 (0.00)	0.00 (0.00)	0.00 (0.00)	0.00 (0.00)	0.00 (0.00)	0.00 (0.00)	0.00 (0.00)	0.00 (0.00)	0.00 (0.00)	0.00 (0.00)
53	Gly 55	0.00 (0.00)	0.00 (0.00)	0.00 (0.00)	0.00 (0.00)	0.00 (0.00)	0.00 (0.00)	0.00 (0.00)	0.00 (0.00)	0.00 (0.00)	0.00 (0.00)
54	Ile 56	6.53 (0.10)	15.40 (0.25)	8.70 (0.14)	13.23 (0.21)	12.73 (0.20)	8.53 (0.14)	11.05 (0.18)	11.89 (0.19)	9.72 (0.16)	10.54 (0.17)
55	Val 57	0.00 (0.00)	0.00 (0.00)	0.00 (0.00)	0.00 (0.00)	0.00 (0.00)	0.00 (0.00)	0.00 (0.00)	0.00 (0.00)	0.00 (0.00)	0.00 (0.00)
56	Pro 58	25.12 (0.40)	20.44 (0.33)	23.10 (0.37)	27.79 (0.44)	18.25 (0.29)	26.27 (0.42)	15.74 (0.25)	14.23 (0.23)	20.60 (0.33)	21.09 (0.34)
57	Gly 59	0.29 (-0.00; -0.00)	0.00 (-0.00; 0.00)	0.00 (-0.00; 0.00)	0.00 (-0.00; 0.00)	0.00 (-0.00; 0.00)	0.00 (-0.00; 0.00)	0.29 (-0.00; -0.00)	0.58 (-0.00; -0.01)	0.00 (-0.00; 0.00)	0.15 (-0.00; -0.00)
58	Asn 60	44.84 (-0.16)	56.13 (-0.22) H	69.98 (-0.28) H	63.04 (-0.31) H	68.51 (-0.30) H	58.54 (-0.32) H	63.52 (-0.32) H	60.34 (-0.17) H	67.42 (-0.33) H	67.62 (-0.22) H
59	Arg 61	79.13 (-1.89) H	73.29 (-1.77) H	70.29 (-1.71) H	23.10 (0.37)	69.67 (-1.82) H	25.94 (0.42)	57.21 (-1.58) H	55.49 (-1.24) H	72.18 (-1.75) H	69.39 (-1.78) H
60	Leu 62	0.00 (0.00)	0.00 (0.00)	0.00 (0.00)	0.00 (0.00)	0.00 (0.00)	0.00 (0.00)	0.00 (0.00)	0.00 (0.00)	0.00 (0.00)	0.00 (0.00)
61	Lys 63	0.00 (0.00)	0.00 (0.00)	0.00 (0.00)	0.00 (0.00)	0.00 (0.00)	0.00 (0.00)	0.00 (0.00)	0.00 (0.00)	0.00 (0.00)	0.00 (0.00)
62	Ile 64	0.00 (0.00)	0.00 (0.00)	0.00 (0.00)	0.00 (0.00)	0.00 (0.00)	0.00 (0.00)	0.00 (0.00)	0.00 (0.00)	0.00 (0.00)	0.00 (0.00)
63	Leu 65	0.00 (0.00)	0.00 (0.00)	0.00 (0.00)	0.00 (0.00)	0.00 (0.00)	0.00 (0.00)	0.00 (0.00)	0.00 (0.00)	0.00 (0.00)	0.00 (0.00)
64	Val 66	0.00 (0.00)	0.00 (0.00)	0.00 (0.00)	0.00 (0.00)	0.00 (0.00)	0.00 (0.00)	0.00 (0.00)	0.00 (0.00)	0.00 (0.00)	0.00 (0.00)
65	Gly 67	0.00 (0.00)	0.00 (0.00)	0.00 (0.00)	0.00 (0.00)	0.00 (0.00)	0.00 (0.00)	0.00 (0.00)	0.00 (0.00)	0.00 (0.00)	0.00 (0.00)
66	Met 68	0.00 (0.00)	0.00 (0.00)	0.00 (0.00)	0.00 (0.00)	0.00 (0.00)	0.00 (0.00)	0.00 (0.00)	0.00 (0.00)	0.00 (0.00)	0.00 (0.00)
67	Tyr 69	0.00 (0.00)	0.00 (0.00)	0.00 (0.00)	0.00 (0.00)	0.00 (0.00)	0.00 (0.00)	0.00 (0.00)	0.00 (0.00)	0.00 (0.00)	0.00 (0.00)
68	Asp 70	0.00 (0.00)	0.00 (0.00)	0.00 (0.00)	0.00 (0.00)	0.00 (0.00)	0.00 (0.00)	0.00 (0.00)	0.00 (0.00)	0.00 (0.00)	0.00 (0.00)
69	Lys 71	0.00 (0.00)	0.00 (0.00)	0.00 (0.00)	0.00 (0.00)	0.00 (0.00)	0.00 (0.00)	0.00 (0.00)	0.00 (0.00)	0.00 (0.00)	0.00 (0.00)
70	Lys 72	0.00 (0.00)	0.00 (0.00)	0.00 (0.00)	0.00 (0.00)	0.00 (0.00)	0.00 (0.00)	0.00 (0.00)	0.00 (0.00)	0.00 (0.00)	0.00 (0.00)
71	Pro 73	0.00 (0.00)	0.00 (0.00)	0.00 (0.00)	0.00 (0.00)	0.00 (0.00)	0.00 (0.00)	0.00 (0.00)	0.00 (0.00)	0.00 (0.00)	0.00 (0.00)
72	Ala 74	0.00 (0.00)	0.00 (0.00)	0.00 (0.00)	0.00 (0.00)	0.00 (0.00)	0.00 (0.00)	0.00 (0.00)	0.00 (0.00)	0.00 (0.00)	0.00 (0.00)

UNIPROT	Interface residue	31_PEST	32_PEST	33_PEST	34_PEST	35_PEST	36_PEST	37_PEST	38_PEST	39_PEST	40_PEST
1	Met 3	0.00 (0.00)	0.00 (0.00)	0.00 (0.00)	0.00 (0.00)	0.00 (0.00)	0.00 (0.00)	0.00 (0.00)	0.00 (0.00)	0.00 (0.00)	0.00 (0.00)
2	LYS 4	0.00 (0.00)	0.00 (0.00)	0.00 (0.00)	0.00 (0.00)	0.00 (0.00)	0.00 (0.00)	0.00 (0.00)	0.00 (0.00)	0.00 (0.00)	0.00 (0.00)
3	Tyr5	0.00 (0.00)	0.00 (0.00)	0.00 (0.00)	0.00 (0.00)	0.00 (0.00)	0.00 (0.00)	0.00 (0.00)	0.00 (0.00)	0.00 (0.00)	0.00 (0.00)
4	Leu 6	0.00 (0.00)	0.00 (0.00)	0.00 (0.00)	0.00 (0.00)	0.00 (0.00)	0.00 (0.00)	0.00 (0.00)	0.00 (0.00)	0.00 (0.00)	0.00 (0.00)
5	Asn 7	0.00 (0.00)	0.00 (0.00)	0.00 (0.00)	0.00 (0.00)	0.00 (0.00)	0.00 (0.00)	0.00 (0.00)	0.00 (0.00)	0.00 (0.00)	0.00 (0.00)
6	Val 8	0.00 (0.00)	0.00 (0.00)	0.00 (0.00)	0.00 (0.00)	0.00 (0.00)	0.00 (0.00)	0.00 (0.00)	0.00 (0.00)	0.00 (0.00)	0.00 (0.00)
7	Leu 9	0.00 (0.00)	0.00 (0.00)	0.00 (0.00)	0.00 (0.00)	0.00 (0.00)	0.00 (0.00)	0.00 (0.00)	0.00 (0.00)	0.00 (0.00)	0.00 (0.00)
8	Ala 10	0.00 (0.00)	0.00 (0.00)	0.00 (0.00)	0.00 (0.00)	0.00 (0.00)	0.00 (0.00)	0.00 (0.00)	0.00 (0.00)	0.00 (0.00)	0.00 (0.00)
9	Lys 11	0.00 (0.00)	0.00 (0.00)	0.00 (0.00)	0.00 (0.00)	0.00 (0.00)	0.00 (0.00)	0.00 (0.00)	0.00 (0.00)	0.00 (0.00)	0.00 (0.00)
10	Ala 12	0.00 (0.00)	0.00 (0.00)	0.00 (0.00)	0.00 (0.00)	0.00 (0.00)	0.00 (0.00)	0.00 (0.00)	0.00 (0.00)	0.00 (0.00)	0.00 (0.00)
11	Leu 13	0.00 (0.00)	0.00 (0.00)	0.00 (0.00)	0.00 (0.00)	0.00 (0.00)	0.00 (0.00)	0.12 (-0.00)	0.00 (0.00)	0.00 (0.00)	0.00 (0.00)
12	Tyr 14	67.23 (1.04)	77.58 (1.10)	75.64 (1.09)	46.57 (0.73)	43.03 (0.63)	61.48 (0.85)	76.48 (1.16)	65.63 (1.04)	64.84 (0.98)	43.94 (0.67)
13	Asp 15	48.16 (-0.62) H	37.65 (-0.43)	12.19 (-0.20)	20.97 (-0.27)	4.19 (-0.02)	3.11 (-0.03)	41.38 (0.17) H	3.24 (-0.02)	10.78 (-0.20)	5.89 (-0.15)
14	Asn 16	8.96 (-0.15)	5.65 (-0.10)	9.81 (-0.17)	16.46 (-0.20)	3.70 (-0.00)	7.98 (-0.14)	7.49 (-0.13)	8.35 (-0.14)	8.10 (-0.14)	10.35 (-0.17)
15	Val 17	31.78 (0.46) H	4.19 (-0.05) H	10.40 (-0.11) H	2.72 (-0.03) H	9.29 (-0.02) H	6.77 (-0.08) H	32.50 (0.36) H	6.13 (-0.07) H	4.22 (-0.03) H	8.40 (-0.09) H
16	Ala 18	0.00 (0.00)	0.00 (0.00)	0.00 (0.00)	0.00 (0.00)	0.00 (0.00)	0.00 (0.00)	0.00 (0.00)	0.00 (0.00)	0.00 (0.00)	0.00 (0.00)
17	Glu 19	24.33 (-0.14) HS	43.85 (-0.16) HS	18.43 (-0.05)	32.40 (-0.11) HS	20.86 (0.28)	45.11 (-0.16) HS	31.48 (-0.03) S	26.14 (-0.04) HS	46.47 (-0.10) HS	11.35 (-0.08)
18	Ser 20	24.51 (0.18)	23.93 (-0.09) H	26.13 (0.04) H	12.21 (0.20)	19.54 (0.26)	11.71 (0.19)	26.11 (-0.06)	17.06 (0.27)	23.67 (-0.10)	4.18 (0.07)
19	Pro 21	0.00 (0.00)	0.00 (0.00)	0.00 (0.00)	0.00 (0.00)	0.00 (0.00)	0.00 (0.00)	0.00 (0.00)	0.00 (0.00)	0.00 (0.00)	0.00 (0.00)
20	Asp 22	20.23 (-0.05) HS	0.61 (-0.01)	4.35 (0.07)	0.00 (0.00)	0.00 (0.00)	0.00 (0.00)	0.00 (0.00)	0.00 (0.00)	0.00 (0.00)	6.75 (-0.16)
21	Glu 23	12.42 (-0.06) S	9.34 (0.14) HS	21.08 (-0.11) HS	10.75 (0.13) HS	16.38 (0.03) HS	11.21 (0.10) HS	13.97 (0.04) HS	19.65 (0.04) HS	7.64 (0.13) HS	28.80 (-0.17) HS
22	Leu 24	0.00 (0.00)	0.00 (0.00)	0.00 (0.00)	0.00 (0.00)	0.00 (0.00)	0.00 (0.00)	0.00 (0.00)	0.00 (0.00)	0.00 (0.00)	0.00 (0.00)
23	Ser 25	0.00 (0.00)	0.00 (0.00)	0.00 (0.00)	0.00 (0.00)	0.00 (0.00)	0.00 (0.00)	0.00 (0.00)	0.00 (0.00)	0.00 (0.00)	0.00 (0.00)
24	Phe 26	0.00 (0.00)	0.00 (0.00)	0.00 (0.00)	0.00 (0.00)	0.00 (0.00)	0.00 (0.00)	0.00 (0.00)	0.00 (0.00)	0.00 (0.00)	0.00 (0.00)
25	Arg 27	67.78 (-1.43) S	0.00 (0.00)	4.22 (-0.16)	0.00 (0.00)	0.00 (0.00)	0.00 (0.00)	27.61 (-0.46) H	0.00 (0.00)	0.00 (0.00)	0.00 (0.00)
26	Lys 28	34.40 (-0.51) HS	45.22 (-0.55) HS	37.69 (0.58)	0.00 (0.00)	11.18 (-0.41)	0.00 (0.00)	50.98 (-0.27) HS	10.34 (-0.17)	32.41 (-1.11) H	0.00 (0.00)
27	Gly 29	0.00 (0.00)	0.00 (0.00)	0.00 (0.00)	0.00 (0.00)	0.00 (0.00)	0.00 (0.00)	0.00 (0.00)	0.00 (0.00)	0.00 (0.00)	0.00 (0.00)
28	Asp 30	0.00 (0.00)	0.00 (0.00)	0.00 (0.00)	0.00 (0.00)	0.00 (0.00)	0.00 (0.00)	0.00 (0.00)	0.00 (0.00)	0.00 (0.00)	0.00 (0.00)
29	Ile 31	0.00 (0.00)	0.00 (0.00)	0.00 (0.00)	0.00 (0.00)	0.00 (0.00)	0.00 (0.00)	0.00 (0.00)	0.00 (0.00)	0.00 (0.00)	0.00 (0.00)
30	Met 32	0.00 (0.00)	0.00 (0.00)	0.00 (0.00)	0.00 (0.00)	0.00 (0.00)	0.00 (0.00)	0.00 (0.00)	0.00 (0.00)	0.00 (0.00)	0.00 (0.00)
31	Thr 33	0.00 (0.00)	0.00 (0.00)	0.00 (0.00)	0.00 (0.00)	0.00 (0.00)	0.00 (0.00)	0.00 (0.00)	0.00 (0.00)	0.00 (0.00)	0.00 (0.00)
32	Val 34	0.00 (0.00)	0.00 (0.00)	0.00 (0.00)	0.00 (0.00)	0.00 (0.00)	0.00 (0.00)	0.00 (0.00)	0.00 (0.00)	0.00 (0.00)	0.00 (0.00)
33	Leu 35	0.00 (0.00)	0.00 (0.00)	0.00 (0.00)	0.00 (0.00)	0.00 (0.00)	0.00 (0.00)	0.00 (0.00)	0.00 (0.00)	0.00 (0.00)	0.00 (0.00)
34	Glu 36	0.00 (0.00)	0.00 (0.00)	0.00 (0.00)	0.00 (0.00)	0.00 (0.00)	0.00 (0.00)	0.00 (0.00)	0.00 (0.00)	0.00 (0.00)	0.00 (0.00)
35	Arg 37	0.00 (0.00)	0.00 (0.00)	0.00 (0.00)	0.00 (0.00)	0.00 (0.00)	0.00 (0.00)	0.00 (0.00)	0.00 (0.00)	0.00 (0.00)	0.00 (0.00)
36	Asp 38	0.00 (0.00)	0.00 (0.00)	0.00 (0.00)	0.00 (0.00)	0.00 (0.00)	0.00 (0.00)	0.00 (0.00)	0.00 (0.00)	0.00 (0.00)	0.00 (0.00)
37	Thr 39	0.00 (0.00)	0.00 (0.00)	0.00 (0.00)	0.00 (0.00)	0.00 (0.00)	0.00 (0.00)	0.00 (0.00)	0.00 (0.00)	0.00 (0.00)	0.00 (0.00)
38	Gln 40	1.10 (-0.02)	0.74 (-0.01)	0.00 (0.00)	0.00 (0.00)	22.69 (-0.24)	2.21 (-0.03)	0.00 (0.00)	2.14 (0.00)	0.00 (0.00)	0.00 (0.00)
39	Gly 41	11.05 (-0.13)	35.00 (-0.13) H	3.68 (-0.04)	0.86 (-0.01)	31.31 (0.00)	19.75 (-0.16)	27.94 (-0.28) H	5.28 (-0.06)	3.06 (-0.03)	1.59 (-0.02)
40	Leu 42	56.99 (0.91)	50.86 (0.81)	41.01 (0.66)	48.86 (0.78)	53.65 (0.86)	50.94 (0.82)	50.66 (0.81)	53.81 (0.86)	33.47 (0.54)	32.46 (0.52)
41	Asp 43	17.53 (-0.18)	20.22 (-0.14)	57.87 (-0.12) HS	26.03 (-0.07)	23.35 (-0.15)	25.33 (-0.19)	46.76 (0.13)	30.73 (-0.16)	41.88 (0.11)	59.87 (-0.07) HS
42	Gly 44	11.32 (0.12)	10.13 (0.12)	11.74 (0.12)	11.04 (0.12)	10.85 (0.13)	10.95 (0.12)	10.97 (0.11)	9.90 (0.08)	10.32 (0.08)	11.32 (0.12)
43	Trp 45	70.86 (0.45)	67.54 (0.39)	69.58 (0.42)	74.01 (0.45)	71.34 (0.47)	73.38 (0.51)	68.04 (0.41)	73.78 (0.43)	70.30 (0.41)	76.31 (0.47)
44	Trp 46	25.26 (0.00)	25.26 (0.00)	25.26 (0.00)	25.26 (0.00)	25.26 (0.00)	25.26 (0.00)	25.26 (0.00)	25.26 (0.00)	25.26 (0.00)	25.26 (0.00)
45	Leu 47	0.00 (0.00)	0.00 (0.00)	0.00 (0.00)	0.00 (0.00)	0.00 (0.00)	0.00 (0.00)	0.00 (0.00)	0.00 (0.00)	0.00 (0.00)	0.00 (0.00)
46	Cys 48	0.00 (0.00)	0.00 (0.00)	0.00 (0.00)	0.00 (0.00)	0.00 (0.00)	0.00 (0.00)	0.00 (0.00)	0.00 (0.00)	0.00 (0.00)	0.00 (0.00)
47	Ser 49	0.00 (0.00)	0.00 (0.00)	0.00 (0.00)	0.00 (0.00)	0.00 (0.00)	0.00 (0.00)	0.00 (0.00)	0.00 (0.00)	0.00 (0.00)	0.00 (0.00)
48	Leu 50	0.00 (0.00)	0.00 (0.00)	0.00 (0.00)	0.00 (0.00)	0.00 (0.00)	0.00 (0.00)	0.00 (0.00)	0.00 (0.00)	0.00 (0.00)	0.00 (0.00)
49	His 51	0.00 (0.00)	0.00 (0.00)	0.00 (0.00)	0.00 (0.00)	0.00 (0.00)	0.00 (0.00)	0.00 (0.00)	0.00 (0.00)	0.00 (0.00)	0.00 (0.00)
50	Gly 52	0.00 (0.00)	0.00 (0.00)	0.00 (0.00)	0.00 (0.00)	0.00 (0.00)	0.00 (0.00)	0.00 (0.00)	0.00 (0.00)	0.00 (0.00)	0.00 (0.00)
51	Arg 53	0.00 (0.00)	0.00 (0.00)	0.00 (0.00)	0.00 (0.00)	0.00 (0.00)	0.00 (0.00)	0.00 (0.00)	0.00 (0.00)	0.00 (0.00)	0.00 (0.00)
52	Gln 54	0.00 (0.00)	0.00 (0.00)	0.00 (0.00)	0.00 (0.00)	0.00 (0.00)	0.00 (0.00)	0.00 (0.00)	0.00 (0.00)	0.00 (0.00)	0.00 (0.00)
53	Gly 55	0.00 (0.00)	0.00 (0.00)	0.00 (0.00)	0.00 (0.00)	0.00 (0.00)	0.00 (0.00)	0.00 (0.00)	0.00 (0.00)	0.00 (0.00)	0.00 (0.00)
54	Ile 56	12.89 (0.21)	9.88 (0.16)	14.73 (0.24)	10.21 (0.16)	8.04 (0.13)	7.20 (0.12)	6.36 (0.10)	8.37 (0.13)	6.70 (0.11)	7.87 (0.13)
55	Val 57	0.00 (0.00)	0.00 (0.00)	0.00 (0.00)	0.00 (0.00)	0.00 (0.00)	0.00 (0.00)	0.00 (0.00)	0.00 (0.00)	0.00 (0.00)	0.00 (0.00)
56	Pro 58	21.60 (0.35)	15.75 (0.25)	18.92 (0.30)	26.62 (0.43)	19.75 (0.32)	20.93 (0.33)	20.09 (0.32)	20.59 (0.33)	20.59 (0.33)	23.78 (0.38)
57	Gly 59	0.43 (-0.00; -0.00)	0.00 (-0.00; 0.00)	0.14 (-0.00; -0.00)	0.58 (-0.00; -0.01)	0.00 (-0.00; 0.00)	0.15 (-0.00; -0.00)	0.58 (-0.00; -0.01)	0.14 (-0.00; -0.00)	0.14 (-0.00; -0.00)	1.02 (-0.00; -0.01)
58	Asn 60	55.75 (-0.07)	62.24 (-0.20)	69.17 (-0.28) H	53.69 (-0.20) H	65.37 (-0.33) H	71.26 (-0.28) H	60.53 (0.01)	52.52 (-0.10)	60.93 (-0.03)	58.27 (0.04)
59	Arg 61	70.90 (-1.55) H	61.45 (-1.36) H	69.28 (-1.88) H	26.27 (0.42)	64.34 (-1.69) H	70.85 (-1.81) H	72.56 (-1.78) H	71.53 (-1.63) H	70.33 (-1.81) H	67.34 (-1.75) H
60	Leu 62	0.00 (0.00)	0.00 (0.00)	0.00 (0.00)	0.00 (0.00)	0.00 (0.00)	0.00 (0.00)	0.00 (0.00)	0.00 (0.00)	0.00 (0.00)	0.00 (0.00)
61	Lys 63	0.00 (0.00)	0.00 (0.00)	0.00 (0.00)	0.00 (0.00)	0.00 (0.00)	0.00 (0.00)	0.00 (0.00)	0.00 (0.00)	0.00 (0.00)	0.00 (0.00)
62	Ile 64	0.00 (0.00)	0.00 (0.00)	0.00 (0.00)	0.00 (0.00)	0.00 (0.00)	0.00 (0.00)	0.00 (0.00)	0.00 (0.00)	0.00 (0.00)	0.00 (0.00)
63	Leu 65	0.00 (0.00)	0.00 (0.00)	0.00 (0.00)	0.00 (0.00)	0.00 (0.00)	0.00 (0.00)	0.00 (0.00)	0.00 (0.00)	0.00 (0.00)	0.00 (0.00)
64	Val 66	0.00 (0.00)	0.00 (0.00)	0.00 (0.00)	0.00 (0.00)	0.00 (0.00)	0.00 (0.00)	0.00 (0.00)	0.00 (0.00)	0.00 (0.00)	0.00 (0.00)
65	Gly 67	0.00 (0.00)	0.00 (0.00)	0.00 (0.00)	0.00 (0.00)	0.00 (0.00)	0.00 (0.00)	0.00 (0.00)	0.00 (0.00)	0.00 (0.00)	0.00 (0.00)
66	Met 68	0.00 (0.00)	0.00 (0.00)	0.00 (0.00)	0.00 (0.00)	0.00 (0.00)	0.00 (0.00)	0.00 (0.00)	0.00 (0.00)	0.00 (0.00)	0.00 (0.00)
67	Tyr 69	0.00 (0.00)	0.00 (0.00)	0.00 (0.00)	0.00 (0.00)	0.00 (0.00)	0.00 (0.00)	0.00 (0.00)	0.00 (0.00)	0.00 (0.00)	0.00 (0.00)
68	Asp 70	0.00 (0.00)	0.00 (0.00)	0.00 (0.00)	0.00 (0.00)	0.00 (0.00)	0.00 (0.00)	0.00 (0.00)	0.00 (0.00)	0.00 (0.00)	0.00 (0.00)
69	Lys 71	0.00 (0.00)	0.00 (0.00)	0.00 (0.00)	0.00 (0.00)	0.00 (0.00)	0.00 (0.00)	0.00 (0.00)	0.00 (0.00)	0.00 (0.00)	0.00 (0.00)
70	Lys 72	0.00 (0.00)	0.00 (0.00)	0.00 (0.00)	0.00 (0.00)	0.00 (0.00)	0.00 (0.00)	0.00 (0.00)	0.00 (0.00)	0.00 (0.00)	0.00 (0.00)
71	Pro 73	0.00 (0.00)	0.00 (0.00)	0.00 (0.00)	0.00 (0.00)	0.00 (0.00)	0.00 (0.00)	0.00 (0.00)	0.00 (0.00)	0.00 (0.00)	0.00 (0.00)
72	Ala 74	0.00 (0.00)	0.00 (0.00)	0.00 (0.00)	0.00 (0.00)	0.00 (0.00)	0.00 (0.00)	0.00 (0.00)	0.00 (0.00)	0.00 (0.00)	0.00 (0.00)

Supplementary table S2. Interface analysis of CAS SH3-PTP-PEST (SH3 part).
Hydrogen bonds and nonpolar contacts between Cas SH3 and its ligands were analyzed with PISA with default settings. Prior to analysis, the linker sequence (residues 75-82) was removed from the chimeric proteins. Solvent accessible residues are colored yellow and inaccessible residues are blue. ΔiG represents free energy of dissociation (solvation energy effect, kcal/mol). BSA means Buried Surface Area (Å²) and is displayed with bars, one bar per 10%. HSDC indicates residues that contain the across-interface hydrogen bond, salt bridge, or disulfide bond atoms. The corresponding table cells have a brown background and contain letter H for a hydrogen bond, S for a salt bridge, and HS for a combination of hydrogen bond and salt bridge.
http://www.ebi.ac.uk/msd-srv/prot_int/pistart.html

(CAS SH3)-PTP-PEST structure number

BSA// ΔiG /HSDC

Position	Interface residue	01 PEST	02 PEST	03 PEST	04 PEST	05 PEST	06 PEST	07 PEST	08 PEST	09 PEST	10 PEST
-6	GLU 84	0.00 / 0.00	0.00 / 0.00	0.00 / 0.00	22.89 / 0.21	0.00 / 0.00	0.00 / 0.00	0.00 / 0.00	0.00 / 0.00	0.00 / 0.00	0.00 / 0.00
-5	LYS 85	26.87 / -0.47 HS	0.00 / 0.00	0.00 / 0.00	0.00 / 0.00	0.00 / 0.00	0.00 / 0.00	0.00 / 0.00	24.86 / -0.78	0.00 / 0.00	0.00 / 0.00
-4	GLN 86	0.00 / 0.00	30.99 / -0.41	0.00 / 0.00	8.10 / -0.05	0.00 / 0.00	0.00 / 0.00	0.00 / 0.00	0.00 / 0.00	0.00 / 0.00	0.00 / 0.00
-3	ASP 87	10.58 / -0.12	0.99 / -0.01	6.15 / -0.01	10.55 / -0.12	64.23 / -0.23 HS	0.00 / 0.00	0.00 / 0.00	8.17 / -0.07	0.00 / 0.00	0.00 / 0.00
-2	SER 88	8.02 / -0.00	8.02 / -0.01	8.02 / -0.00	8.02 / 0.00	8.02 / -0.11	8.02 / 0.01	8.02 / 0.00	8.02 / -0.10	8.02 / 0.01	8.02 / 0.01
-1	**PRO 89**	73.27 / 0.71	59.90 / 0.71	65.03 / 0.71	84.52 / 0.71	49.77 / 0.71	61.58 / 0.71	61.71 / 0.71	52.24 / 0.71	61.24 / 0.71	52.15 / 0.71
0	**PRO 90**	78.78 / 0.91 H	65.75 / 0.69 H	68.39 / 0.70 H	51.39 / 0.46 H	76.60 / 0.93 H	63.52 / 0.63 H	77.30 / 0.84 H	79.70 / 0.94 H	79.48 / 0.87 H	66.62 / 0.90
1	PRO 91	19.96 / -0.05	14.36 / -0.10 H	14.42 / -0.07 H	10.51 / -0.07 H	15.94 / -0.09 H	12.77 / -0.08 H	25.88 / -0.05	23.71 / -0.06 H	26.33 / -0.04 H	14.17 / -0.13 H
2	**LYS 92**	105.60 / -0.57 HS	102.15 / -0.33 HS	95.91 / -0.51 HS	119.63 / -0.65 HS	102.59 / -0.15 HS	106.99 / -0.36 HS	107.21 / -0.58 HS	125.42 / -0.53 HS	121.16 / -0.60 HS	102.34 / -0.52 HS
3	**PRO 93**	74.22 / 1.14	79.26 / 1.18	77.69 / 1.20	84.17 / 1.28	81.07 / 1.21	84.88 / 1.30	89.97 / 1.34	82.57 / 1.24	81.51 / 1.24	81.19 / 1.26
4	PRO 94	20.12 / -0.14	15.60 / -0.16	20.72 / -0.11	25.26 / -0.06	18.94 / -0.16	30.50 / -0.06	19.55 / -0.13	23.29 / -0.14	14.21 / -0.14	27.13 / -0.01
5	**ARG 95**	110.41 / -0.02 HS	130.29 / -0.18 HS	112.08 / -0.33 HS	119.81 / -1.14 HS	148.53 / -1.43 HS	120.41 / -0.08 HS	118.56 / -0.10 HS	147.07 / -0.77 HS	140.16 / -0.43 H	101.80 / -0.02 HS
6	THR 96	17.07 / -0.19	1.51 / 0.02	28.83 / 0.23	44.52 / 0.51	1.67 / 0.03	0.84 / 0.01	7.05 / 0.02	2.13 / 0.03	0.50 / 0.01	56.57 / 0.68
7	ARG 97	32.19 / 0.12	57.52 / -0.79	35.37 / -0.39 HS	47.54 / -1.11 H	25.01 / 0.19	29.76 / 0.20	46.72 / 0.63	63.64 / -0.99 H	64.68 / -0.94 H	1.00 / 0.02
8	SER 98	0.00 / 0.00	0.00 / 0.00	0.00 / 0.00	0.00 / 0.00	0.00 / 0.00	0.00 / 0.00	0.00 / 0.00	0.00 / 0.00	0.00 / 0.00	0.00 / 0.00
9	CYS 99	0.00 / 0.00	0.00 / 0.00	0.00 / 0.00	0.00 / 0.00	0.00 / 0.00	0.00 / 0.00	0.00 / 0.00	0.00 / 0.00	0.00 / 0.00	0.00 / 0.00
10	LEU 100	0.00 / 0.00	0.00 / 0.00	0.00 / 0.00	0.00 / 0.00	0.99 / 0.02	0.00 / 0.00	0.00 / 0.00	0.00 / 0.00	0.00 / 0.00	0.00 / 0.00

Position	Interface residue	11 PEST	12 PEST	13 PEST	14 PEST	15 PEST	16 PEST	17 PEST	18 PEST	19 PEST	20 PEST
-6	GLU 84	26.38 / 0.23	0.00 / 0.00	0.00 / 0.00	0.00 / 0.00	0.00 / 0.00	0.00 / 0.00	0.00 / 0.00	62.73 / -0.59 HS	0.00 / 0.00	0.00 / 0.00
-5	LYS 85	13.78 / 0.14	0.00 / 0.00	0.00 / 0.00	0.00 / 0.00	8.20 / -0.07	0.00 / 0.00	0.00 / 0.00	0.00 / 0.00	6.17 / 0.10	0.00 / 0.00
-4	GLN 86	0.00 / 0.00	4.08 / -0.05	0.00 / 0.00	0.00 / 0.00	0.00 / 0.00	0.00 / 0.00	0.00 / 0.00	10.54 / -0.12	0.00 / 0.00	12.89 / -0.20
-3	ASP 87	2.09 / -0.02	4.41 / -0.05	0.00 / 0.00	8.62 / -0.10	16.13 / -0.06	0.00 / 0.00	21.16 / -0.31	7.61 / -0.09	20.74 / -0.35	38.10 / -0.04
-2	SER 88	8.02 / 0.01	8.02 / 0.00	8.02 / 0.00	8.02 / 0.01	8.02 / -0.05	8.02 / -0.00	8.02 / 0.00	8.02 / 0.00	8.02 / -0.03	8.02 / -0.03
-1	**PRO 89**	55.42 / 0.71	67.08 / 0.71	62.55 / 0.71	63.20 / 0.71	51.63 / 0.71	65.43 / 0.71	41.94 / 0.71	72.08 / 0.71	72.40 / 0.71	59.50 / 0.71
0	**PRO 90**	70.51 / 0.73 H	63.66 / 0.61 H	85.00 / 0.96 H	82.11 / 0.96 H	78.71 / 0.93 H	79.19 / 0.87 H	76.53 / 1.02	68.22 / 0.68 H	81.68 / 0.94 H	72.59 / 0.84 H
1	PRO 91	27.13 / -0.05 H	14.67 / -0.09	26.21 / -0.03	18.85 / -0.12	22.80 / -0.09	26.62 / -0.08 H	20.90 / -0.08 H	20.24 / -0.08 H	25.12 / -0.05 H	17.31 / -0.08 H
2	**LYS 92**	125.17 / -0.64 HS	105.71 / -0.40 HS	129.08 / -0.50 HS	109.15 / -0.37 HS	96.58 / -0.64 HS	115.57 / -0.72 HS	101.64 / -0.35 HS	121.63 / -0.63 HS	120.44 / -0.57 HS	114.60 / -0.51 HS
3	**PRO 93**	79.14 / 1.14	84.65 / 1.28	82.90 / 1.22	86.20 / 1.29	76.06 / 1.14	74.39 / 1.12	82.01 / 1.21	83.74 / 1.26	78.02 / 1.17	85.15 / 1.32
4	PRO 94	15.84 / -0.17	24.25 / -0.09	19.07 / -0.15	26.83 / -0.11	13.86 / -0.16	14.85 / -0.12	17.55 / -0.15	20.32 / -0.10	19.60 / -0.16	28.43 / -0.03
5	**ARG 95**	154.14 / -0.77 HS	115.38 / -0.40 HS	150.74 / -1.04 HS	117.68 / 0.17 H	133.05 / -0.44 HS	83.34 / 0.45 HS	128.65 / -0.12 H	123.29 / -0.97 HS	119.40 / -0.35	94.62 / -0.10
6	THR 96	0.67 / 0.01	45.48 / 0.45	0.67 / 0.01	0.00 / 0.00	2.01 / 0.03	25.34 / 0.23	5.08 / 0.05	54.93 / 0.53	0.00 / 0.00	46.80 / 0.49
7	ARG 97	60.84 / -1.06 H	0.00 / 0.00	57.18 / -0.26 S	35.79 / -0.27	65.00 / 0.21 H	4.57 / -0.17	78.55 / -0.21 HS	2.17 / 0.03	52.76 / -1.17 HS	0.00 / 0.00
8	SER 98	0.00 / 0.00	0.00 / 0.00	0.00 / 0.00	0.00 / 0.00	0.00 / 0.00	0.00 / 0.00	2.18 / -0.02	0.00 / 0.00	0.00 / 0.00	0.00 / 0.00
9	CYS 99	0.00 / 0.00	0.00 / 0.00	0.00 / 0.00	0.00 / 0.00	0.00 / 0.00	4.45 / 0.18	5.53 / 0.05	0.00 / 0.00	0.00 / 0.00	0.00 / 0.00
10	LEU 100	0.00 / 0.00	0.00 / 0.00	19.52 / 0.31	0.00 / 0.00	0.00 / 0.00	0.00 / 0.00	0.00 / 0.00	0.00 / 0.00	2.09 / 0.03	0.00 / 0.00

Position	Interface residue	21 PEST	22 PEST	23 PEST	24 PEST	25 PEST	26 PEST	27 PEST	28 PEST	29 PEST	30 PEST
-6	GLU 84	49.52 / -0.27 HS	0.00 / 0.00	8.82 / 0.04	2.70 / -0.03	42.66 / -0.18 HS	7.41 / 0.11	6.69 / -0.05	0.00 / 0.00	18.87 / 0.30	28.26 / -0.30 HS
-5	LYS 85	20.14 / -0.10 H	0.00 / 0.00	4.29 / -0.05	0.00 / 0.00	4.53 / 0.07	0.00 / 0.00	0.49 / -0.01	80.20 / 0.15 HS	0.17 / 0.00	0.00 / 0.00
-4	GLN 86	54.45 / 0.37	0.31 / 0.01	18.31 / -0.25	0.00 / 0.00	8.67 / 0.13	0.00 / 0.00	1.96 / -0.03	61.40 / -0.38 H	0.00 / 0.00	0.00 / 0.00
-3	ASP 87	6.35 / 0.09	68.37 / -0.32 HS	63.60 / -0.25 HS	0.37 / -0.00	75.60 / -0.16 HS	33.55 / -0.33 H	3.55 / -0.04	0.00 / 0.00	65.86 / -0.36 HS	59.36 / -0.20 HS
-2	SER 88	8.02 / -0.01	8.02 / -0.08	8.02 / -0.09	8.02 / 0.00	8.02 / -0.08	8.02 / -0.02	8.02 / -0.01	8.02 / 0.00	8.02 / -0.10	8.02 / -0.07
-1	**PRO 89**	61.05 / 0.71	56.25 / 0.71	57.57 / 0.71	32.88 / 0.71	60.43 / 0.71	54.33 / 0.71	68.41 / 0.71	53.72 / 0.71	59.97 / 0.71	58.25 / 0.71
0	**PRO 90**	81.82 / 0.99 H	76.12 / 0.93 H	76.47 / 0.87 H	69.40 / 0.94	69.39 / 0.85 H	66.48 / 0.92	70.43 / 0.79 H	65.00 / 0.68 H	76.46 / 0.91 H	72.21 / 0.86 H
1	PRO 91	17.69 / -0.07	14.17 / -0.09 H	24.81 / -0.01 H	19.24 / -0.10 H	19.87 / -0.09 H	21.60 / -0.15 H	11.75 / -0.07 H	14.90 / -0.09 H	23.82 / -0.06 H	15.51 / -0.07 H
2	**LYS 92**	112.63 / -0.56 HS	130.91 / -0.49 HS	97.43 / -0.69 HS	68.23 / 1.08	107.70 / -0.52 HS	64.95 / -0.43 H	118.00 / -0.55 HS	135.39 / -0.40 HS	126.45 / -0.63 HS	119.13 / -0.47 HS
3	**PRO 93**	78.11 / 1.20	85.39 / 1.27	81.04 / 1.25	83.22 / 1.17	79.28 / 1.22	76.23 / 1.13	81.87 / 1.19	83.11 / 1.21	85.72 / 1.24	91.74 / 1.39
4	PRO 94	28.24 / -0.06	20.05 / -0.05	21.75 / -0.12	22.18 / -0.07	19.55 / -0.13	14.11 / -0.16	15.04 / -0.14	11.66 / -0.13	12.06 / -0.13	19.49 / 0.00
5	**ARG 95**	72.61 / 0.41	141.45 / -1.06 HS	105.20 / -0.29 HS	118.35 / -0.56 HS	123.07 / -1.71 HS	140.45 / -0.32 HS	107.51 / 0.10	145.27 / -1.63 HS	142.11 / -1.20 HS	109.86 / -0.13
6	THR 96	0.84 / 0.01	0.83 / 0.01	36.39 / 0.27	48.71 / 0.52	30.91 / 0.39	18.17 / -0.10	16.06 / 0.26	29.59 / 0.22	44.36 / 0.46	53.73 / 0.24
7	ARG 97	54.11 / 0.00 H	63.81 / 0.40 H	0.00 / 0.00	0.00 / 0.00	40.47 / -1.06 H	5.35 / 0.09	0.00 / 0.00	0.00 / 0.00	9.99 / -0.11	12.04 / 0.19
8	SER 98	0.00 / 0.00	0.00 / 0.00	0.00 / 0.00	0.00 / 0.00	0.00 / 0.00	32.08 / 0.32	0.00 / 0.00	0.00 / 0.00	0.00 / 0.00	6.36 / -0.05
9	CYS 99	0.00 / 0.00	0.00 / 0.00	0.00 / 0.00	0.00 / 0.00	0.00 / 0.00	0.00 / 0.00	0.00 / 0.00	0.00 / 0.00	0.00 / 0.00	39.23 / 1.16
10	LEU 100	0.00 / 0.00	0.00 / 0.00	0.00 / 0.00	0.00 / 0.00	0.00 / 0.00	2.68 / 0.04	0.00 / 0.00	0.00 / 0.00	0.00 / 0.00	0.00 / 0.00

Position	Interface residue	31 PEST	32 PEST	33 PEST	34 PEST	35 PEST	36 PEST	37 PEST	38 PEST	39 PEST	40 PEST
-6	GLU 84	46.14 / -0.21 S	6.38 / -0.07	6.18 / -0.02	0.00 / 0.00	11.05 / -0.08	0.00 / 0.00	30.47 / -0.31 H	0.00 / 0.00	0.00 / 0.00	0.00 / 0.00
-5	LYS 85	40.62 / 0.65	9.45 / -0.11	31.69 / 0.46	0.00 / 0.00	0.84 / 0.01	0.00 / 0.00	44.98 / 0.48	0.00 / 0.00	0.00 / 0.00	0.00 / 0.00
-4	GLN 86	31.38 / 0.18 H	25.59 / 0.20	0.00 / 0.00	0.00 / 0.00	0.00 / 0.00	0.00 / 0.00	75.21 / -0.45 H	0.00 / 0.00	35.48 / -0.47 H	0.00 / 0.00
-3	ASP 87	58.92 / -0.21 HS	51.49 / -0.10 HS	24.88 / 0.25	3.92 / -0.04	0.00 / 0.00	16.92 / -0.16	53.62 / -0.31 HS	34.14 / -0.04	10.80 / -0.12	0.00 / 0.00
-2	SER 88	8.02 / -0.08	8.02 / -0.12	8.02 / -0.08	8.02 / -0.02	8.02 / 0.01	8.02 / -0.01	8.02 / -0.06	8.02 / -0.01	8.02 / -0.03	8.02 / 0.00
-1	**PRO 89**	48.95 / 0.71	31.82 / 0.71	58.97 / 0.71	58.40 / 0.71	61.92 / 0.71	59.82 / 0.71	55.10 / 0.71	56.46 / 0.71	67.29 / 0.71	65.03 / 0.71
0	**PRO 90**	81.84 / 0.97 H	80.36 / 0.96 H	77.12 / 0.91 H	74.85 / 1.01	62.35 / 0.64 H	78.66 / 0.90 H	78.19 / 0.95 H	81.00 / 0.90 H	78.82 / 0.94 H	84.45 / 0.97 H
1	PRO 91	25.88 / -0.02	24.42 / -0.03	19.90 / -0.11 H	17.50 / -0.15 H	12.32 / -0.07 H	23.74 / -0.04 H	23.52 / -0.06	16.23 / -0.03	24.22 / -0.08	20.48 / -0.02
2	**LYS 92**	117.72 / -0.73 HS	103.25 / -0.55 HS	110.98 / -0.63 HS	98.64 / -0.52 HS	104.90 / -0.22 HS	112.32 / -0.61 HS	100.59 / -0.52 HS	107.24 / -0.45 HS	105.08 / -0.54 HS	95.44 / -0.35 HS
3	**PRO 93**	76.70 / 1.14	83.01 / 1.23	86.80 / 1.34	81.65 / 1.22	85.93 / 1.28	82.02 / 1.27	78.74 / 1.17	78.90 / 1.22	79.42 / 1.18	80.68 / 1.19
4	PRO 94	15.81 / -0.16	16.54 / -0.17	19.48 / -0.16	16.05 / -0.12	21.29 / -0.12	26.74 / -0.07	16.54 / -0.17	30.33 / -0.03	14.11 / -0.16	23.42 / -0.09
5	**ARG 95**	127.18 / -0.88 HS	134.81 / -0.38 HS	140.84 / -1.20 HS	139.73 / -0.52 HS	112.92 / 0.06 HS	116.54 / -0.53 HS	130.73 / -0.28 HS	98.62 / 0.13 HS	135.80 / -0.15 HS	102.23 / 0.01 HS
6	THR 96	34.55 / 0.36	1.15 / 0.01	0.00 / 0.00	16.56 / 0.26	5.83 / 0.06	26.78 / -0.12	2.46 / 0.04	27.69 / 0.27	0.00 / 0.00	0.00 / 0.00
7	ARG 97	7.28 / -0.27	48.65 / 0.11 H	48.66 / -0.25 HS	0.00 / 0.00	65.86 / -0.18	0.00 / 0.00	74.65 / -0.10 H	2.03 / -0.08	30.48 / -0.65	37.69 / -0.11 HS
8	SER 98	0.00 / 0.00	0.00 / 0.00	0.00 / 0.00	0.00 / 0.00	0.00 / 0.00	0.00 / 0.00	0.00 / 0.00	0.00 / 0.00	0.00 / 0.00	0.00 / 0.00
9	CYS 99	0.00 / 0.00	0.00 / 0.00	0.00 / 0.00	0.00 / 0.00	0.00 / 0.00	0.00 / 0.00	0.00 / 0.00	0.00 / 0.00	0.00 / 0.00	0.00 / 0.00
10	LEU 100	0.00 / 0.00	0.00 / 0.00	0.00 / 0.00	0.00 / 0.00	0.00 / 0.00	14.48 / 0.23	0.00 / 0.00	0.00 / 0.00	0.00 / 0.00	0.00 / 0.00

	PEST			
SDE	-6	327	E	
	-5	328	K	
	-4	329	Q	
	-3	330	D	HS
	-2	331	S	
core	-1	332	**P**	
	0	333	**P**	H
	1	334	P	H
	2	335	**K**	HS
	3	336	**P**	
	4	337	P	
	5	338	**R**	
	6	339	T	
SDE	7	340	R	HS
	8	341	S	
	9	342	C	
	10	343	L	

| per 10% of BSA — BSA = burried of accessible surface area

-

|-||

|||-||||

|||||-||||||

|||||||-|||||||||

Supplementary table S3. Interface analysis of CAS SH3-PTP-PEST (ligand part).

Hydrogen bonds and nonpolar contacts between Cas SH3 and its ligands were analyzed with PISA with default settings. Prior to analysis, the linker sequence (residues 75-82) was removed from the chimeric proteins. Solvent accessible residues are colored yellow and inaccessible residues are blue. ΔiG represents free energy of dissociation (solvation energy effect, kcal/mol). BSA means Buried Surface Area (Å²) and is displayed with bars, one bar per 10%. HSDC indicates residues that contain the across-interface hydrogen bond, salt bridge, or disulfide bond atoms. The corresponding table cells have a brown background and contain letter H for a hydrogen bond, S for a salt bridge, and HS for a combination of hydrogen bond and salt bridge.

http://www.ebi.ac.uk/msd-srv/prot_int/pistart.html

Hydrogen bonds (Interface) XML

Structure (number)

##	UNIPROT	CAS SH3	PTP-PEST		1	2	3	4	5	6	7	8	9	10	11	12	13	14	15	16	17	18	19	20	21	22	23	24	25	26	27	28	29	30	31	32	33	34	35	36	37	38	39	40	SDE	Pocket S1	Pocket S2	Pocket S3	SDE (ext. S3)
1	Lys26	A:LYS 28[NZ]	B:GLU 84[OE1]	-6																		X												X											Lys26				
2		A:LYS 28[NZ]	B:GLU 84[OE2]	-6																																													
3	Arg25	A:ARG 27[NH1]	B:GLU 84[OE1]	-6																									X																				
4		A:ARG 27[NH1]	B:GLU 84[OE2]	-6																					X																				Arg25				
5		A:ARG 27[NH2]	B:GLU 84[OE2]	-6																									X																				
6		A:ARG 27[HE]	B:GLU 84[O]	-6																																					X				Arg25				
7		A:ARG 27[NH2]	B:GLU 84[O]	-6																																					X								
8	Arg25	A:ARG 27[NH2]	B:LYS 85[O]	-5																					X																				Arg25				
9	Asp13	A:ASP 15[OD1]	B:LYS 85[NZ]	-5																																									Asp13				
10	Asp13	A:ASP 15[OD1]	B:LYS 85[NZ]	-5	X																											X													Asp13				
11	Asp13	A:ASP 15[OD2]	B:GLN 86[H]	-4																																					X				Asp13				
12		A:ASP 15[OD1]	B:GLN 86[H]	-4																															X														
13	Asp13	A:ASP 15[OD1]	B:GLN 86[NE2]	-4																																					X				Asp13				
14	Lys26	A:LYS 28[NZ]	B:GLN 86[OE1]	-4																												X											X		Lys26				
15	Lys26	A:LYS 28[NZ]	B:ASP 87[OD1]	-3					X																		X		X				X		X						X				Lys26				
16		A:LYS 28[NZ]	B:ASP 87[OD2]	-3																						X								X	X	X													
17	Tyr12	A:TYR 14[HH]	B:ASP 87[OD2]	-3																										X															Tyr12	Tyr12			
18	Arg59	A:ARG 61[NH2]	B:PRO 90[O]	0	X	X	X	X	X	X	X	X	X		X	X	X	X	X	X		X	X	X	X	X	X		X		X	X	X	X	X	X	X		X	X	X	X	X	X		Arg59	Arg59		
19	Arg59	A:ARG 61[HE]	B:PRO 90[O]	0	X						X						X																		X							X				Arg59	Arg59		
20	Asn58	A:ASN 60[ND2]	B:PRO 90[O]	0		X	X	X		X						X															X	X							X							Asn58	Asn58		
21	Asn58	A:ASN 60[ND2]	B:PRO 91[O]	1		X	X	X	X	X		X	X	X	X					X	X	X	X	X		X	X	X	X	X	X	X	X	X				X	X	X	X						Asn58	Asn58		
22	Glu17	A:GLU 19[OE1]	B:LYS 92[NZ]	2		X	X			X			X		X	X										X							X	X				X					X				Glu17	Glu17	
23		A:GLU 19[OE2]	B:LYS 92[NZ]	2	X							X		X			X												X			X			X	X						X					Glu17	Glu17	
24	Val15	A:VAL 17[O]	B:LYS 92[NZ]	2	X	X	X	X	X	X	X	X	X	X	X	X	X	X	X	X	X	X	X	X	X	X	X		X	X	X	X	X	X	X	X	X	X	X	X	X	X	X	X			Val15		
26	Glu21	A:GLU 23[OE1]	B:LYS 92[NZ]	2			X	X		X	X	X		X	X	X			X				X			X	X				X	X		X				X		X			X				Glu21	Glu21	
27		A:GLU 23[OE2]	B:LYS 92[NZ]	2	X	X		X	X			X	X	X	X		X	X		X	X	X		X	X	X	X		X		X		X	X		X	X		X	X	X	X		X			Glu21	Glu21	
28	Asn14	A:ASN 16[OD1]	B:LYS 92[NZ]	2																										X																	Asn14		
29	Glu21	A:GLU 23[OE1]	B:ARG 95[NH1]	5	X				X		X			X															X							X	X				X								
30		A:GLU 23[OE1]	B:ARG 95[NH2]	5		X																								X									X										
31		A:GLU 23[OE2]	B:ARG 95[NH1]	5			X			X						X			X								X											X				X	X				Glu21	Glu21	
32		A:GLU 23[OE2]	B:ARG 95[NH2]	5												X														X														X					
33	Glu17	A:GLU 19[OE1]	B:ARG 95[NH1]	5										X						X																				X			X						
34		A:GLU 19[OE1]	B:ARG 95[NH2]	5		X				X				X																								X					X				Glu17	Glu17	
35		A:GLU 19[OE2]	B:ARG 95[NH1]	5	X																								X							X													
36		A:GLU 19[OE2]	B:ARG 95[NH2]	5	X		X																													X													
37	Ser18	A:SER 20[OG]	B:ARG 95[HE]	5															X																	X												Ser18	
38	Ser18	A:SER 20[OG]	B:ARG 95[NH1]	5	X			X		X			X			X		X			X					X	X		X			X					X											Ser18	
39		A:SER 20[OG]	B:ARG 95[NH2]	5					X		X		X								X																												
40	Asp20	A:ASP 22[OD1]	B:ARG 95[NH1]	5			X	X																																									
41		A:ASP 22[OD1]	B:ARG 95[NH2]	5				X							X		X											X				X			X													Asp20	Asp20
42		A:ASP 22[OD2]	B:ARG 95[NH1]	5																																													
43		A:ASP 22[OD2]	B:ARG 95[NH2]	5								X										X				X							X																
44	Asp20	A:ASP 22[OD1]	B:ARG 97[NH1]	7			X																																										
45	Gly39	A:GLY 41[O]	B:ARG 97[NH2]	7								X			X										X																								Gly39
46		A:GLY 41[O]	B:ARG 97[NH1]	7									X						X							X										X					X								
47	Gln38	A:GLN 40[OE1]	B:ARG 97[NH2]	7				X																					X				X																Gln38
48	Asp41	A:ASP 43[OD1]	B:ARG 97[NH1]	7																																	X												
49		A:ASP 43[OD1]	B:ARG 97[NH2]	7																	X																												Asp41
50		A:ASP 43[OD2]	B:ARG 97[NH1]	7																			X																					X					
51		A:ASP 43[OD2]	B:ARG 97[NH2]	7																																													

Salt bridges (Interface) XML

Structure (number)

##	UNIPROT	CAS SH3	PTP-PEST		1	2	3	4	5	6	7	8	9	10	11	12	13	14	15	16	17	18	19	20	21	22	23	24	25	26	27	28	29	30	31	32	33	34	35	36	37	38	39	40	SDE	Pocket S1	Pocket S2	Pocket S3	SDE (ext. S3)
1	Lys26	A:LYS 28[NZ]	B:GLU 84[OE1]	-6																		X												X											Lys26				
2		A:LYS 28[NZ]	B:GLU 84[OE2]	-6																		X																											
3	Arg25	A:ARG 27[NH1]	B:GLU 84[OE1]	-6																									X																				
4		A:ARG 27[NH2]	B:GLU 84[OE1]	-6																									X																Arg25				
5		A:ARG 27[NH1]	B:GLU 84[OE2]	-6																					X				X																				
6		A:ARG 27[NH2]	B:GLU 84[OE2]	-6																					X				X						X														
7	Asp13	A:ASP 15[OD1]	B:LYS 85[NZ]	-5	X																											X													Asp13				
8	Lys26	A:LYS 28[NZ]	B:ASP 87[OD1]	-3					X																		X		X				X	X	X	X					X				Lys26				
9		A:LYS 28[NZ]	B:ASP 87[OD2]	-3					X																	X			X					X	X														
10	Glu21	A:GLU 23[OE1]	B:LYS 92[NZ]	2	X	X	X	X	X	X	X	X	X	X	X	X	X	X	X	X	X	X	X	X	X	X	X		X		X	X	X	X	X	X	X	X	X	X	X	X	X	X			Glu21	Glu21	
11		A:GLU 23[OE2]	B:LYS 92[NZ]	2	X	X	X	X	X	X	X	X	X	X	X	X	X	X	X	X		X	X	X	X	X	X		X		X	X	X	X	X	X	X	X	X	X	X	X	X	X					
12	Glu17	A:GLU 19[OE1]	B:LYS 92[NZ]	2			X			X			X		X	X				X						X							X	X	X	X		X					X				Glu17	Glu17	
13		A:GLU 19[OE2]	B:LYS 92[NZ]	2	X	X						X		X			X			X			X						X			X			X	X					X	X							
14	Glu21	A:GLU 23[OE1]	B:ARG 95[NE]	5		X																								X									X								Glu21	Glu21	
15		A:GLU 23[OE2]	B:ARG 95[NE]	5																										X														X					
16	Glu21	A:GLU 23[OE1]	B:ARG 95[NH1]	5	X		X			X	X																		X							X	X				X		X						
17		A:GLU 23[OE1]	B:ARG 95[NH2]	5		X								X		X													X	X								X	X					X			Glu21	Glu21	
18		A:GLU 23[OE2]	B:ARG 95[NH1]	5			X		X							X			X								X											X				X	X						
19		A:GLU 23[OE2]	B:ARG 95[NH2]	5		X										X														X								X	X					X					
20	Glu17	A:GLU 19[OE2]	B:ARG 95[NE]	5																										X																	Glu17	Glu17	
21	Glu17	A:GLU 19[OE1]	B:ARG 95[NH1]	5		X	X			X										X										X										X			X						
22		A:GLU 19[OE1]	B:ARG 95[NH2]	5		X	X			X																				X								X		X			X				Glu17	Glu17	
23		A:GLU 19[OE2]	B:ARG 95[NH1]	5	X									X															X	X						X				X									
24		A:GLU 19[OE2]	B:ARG 95[NH2]	5	X									X																						X				X									
25	Asp20	A:ASP 22[OD1]	B:ARG 95[NH1]	5				X							X		X					X																											
26		A:ASP 22[OD1]	B:ARG 95[NH2]	5				X							X		X					X				X		X				X	X		X													Asp20	Asp20
27		A:ASP 22[OD2]	B:ARG 95[NH1]	5																													X																
28		A:ASP 22[OD2]	B:ARG 95[NH2]	5																													X																
29	Asp20	A:ASP 22[OD1]	B:ARG 97[NH1]	7			X																																									Asp20	Asp20
30	Asp41	A:ASP 43[OD1]	B:ARG 97[NH1]	7													X																				X												
31		A:ASP 43[OD1]	B:ARG 97[NH2]	7													X				X																											Asp41	Asp41
32		A:ASP 43[OD2]	B:ARG 97[NH1]	7													X						X																					X					

SH3 numbering from UNIPROT: X-2

number of structures
0-5 %
6-20 %
21-50 %
51-80%
81-100%

	PTP-PEST		structure	UNIPROT
SDE	-6	E	84	327
	-5	K	85	328
	-4	Q	86	329
	-3	D	87	330
	-2	S	88	331
core	-1	**P**	89	332
	0	**P**	90	333
	1	P	91	334
	2	**K**	92	335
	3	**P**	93	336
	4	P	94	337
	5	**R**	95	338
	6	T	96	339
SDE	7	R	97	340
	8	S	98	341
	9	C	99	342
	10	L	100	343

Supplementary table S4. Interface analysis of CAS SH3-PTP-PEST (hydrogen bonds).
Hydrogen bonds and salt bridges between Cas SH3 and its ligands were analyzed with PISA with default settings. Prior to analysis, the linker sequence (residues 75-82) was removed from the chimeric proteins. The atoms that form hydrogen bonds and salt bridges are indicated by crosses.

| per 10% of BSA

BSA = burried of accessible surface area

SDE	Pocket S1	Pocket S2	Pocket S3	SDE (ext. S3)
Leu11				
Tyr12	Tyr12			
Asp13				
		Asn14		
		Val15		
		Glu17	Glu17	
			Ser18	
				Pro19
			Asp20	Asp20
		Glu21	Glu21	
Lys26				
				Gln38
				Gly39
			Leu40	Leu40
				Asp41
				Gly42
		Trp43	Trp43	
		Trp44	Trp44	
			Ile54	
		Pro56		
		Gly57		
	Asn58	Asn58		
	Arg59	Arg59		

Vinculin

	-6	854	L	S
	-5	855	T	
SDE	-4	856	D	
	-3	857	E	HS
	-2	858	L	
	-1	859	**A**	
	0	860	**P**	H
	1	861	P	H
core	2	862	**K**	HS
	3	863	**P**	
	4	864	P	
	5	865	**L**	
	6	866	P	
	7	867	E	
SDE	8	868	G	
	9	869	E	
	10	870	V	

CAS SH3 (Vinculin) structure — each cell: BSA / ΔG / HSDC

Numbering from UNIPROT: Vinculin (UNIPROT)	Interface residue	01_Vcl	02_Vcl	03_Vcl	04_Vcl	05_Vcl	06_Vcl	07_Vcl	08_Vcl	09_Vcl	10_Vcl	11_Vcl	12_Vcl	13_Vcl	14_Vcl	15_Vcl	16_Vcl	17_Vcl	18_Vcl	19_Vcl	20_Vcl	21_Vcl	22_Vcl	23_Vcl	24_Vcl	25_Vcl	26_Vcl	27_Vcl	28_Vcl	29_Vcl	30_Vcl	31_Vcl	32_Vcl	33_Vcl	34_Vcl	35_Vcl	36_Vcl	37_Vcl	38_Vcl	39_Vcl	40_Vcl
1	Met 3	0.00 / 0.00	0.00 / 0.00	0.00 / 0.00	0.00 / 0.00	0.00 / 0.00	0.00 / 0.00	0.00 / 0.00	0.00 / 0.00	0.00 / 0.00	0.00 / 0.00	0.00 / 0.00	0.00 / 0.00	0.00 / 0.00	0.00 / 0.00	0.00 / 0.00	0.00 / 0.00	0.00 / 0.00	0.00 / 0.00	0.00 / 0.00	0.00 / 0.00	0.00 / 0.00	0.00 / 0.00	0.00 / 0.00	0.00 / 0.00	0.00 / 0.00	0.00 / 0.00	0.00 / 0.00	0.00 / 0.00	0.00 / 0.00	0.00 / 0.00	0.00 / 0.00	0.00 / 0.00	0.00 / 0.00	0.00 / 0.00	0.00 / 0.00	0.00 / 0.00	0.00 / 0.00	0.00 / 0.00	0.00 / 0.00	0.00 / 0.00
2	LYS 4	0.00 / 0.00	0.00 / 0.00	0.00 / 0.00	0.00 / 0.00	0.00 / 0.00	0.00 / 0.00	0.00 / 0.00	0.00 / 0.00	0.00 / 0.00	0.00 / 0.00	0.00 / 0.00	0.00 / 0.00	0.00 / 0.00	0.00 / 0.00	0.00 / 0.00	0.00 / 0.00	0.00 / 0.00	0.00 / 0.00	0.00 / 0.00	0.00 / 0.00	0.00 / 0.00	0.00 / 0.00	0.00 / 0.00	0.00 / 0.00	0.00 / 0.00	0.00 / 0.00	0.00 / 0.00	0.00 / 0.00	0.00 / 0.00	0.00 / 0.00	0.00 / 0.00	0.00 / 0.00	0.00 / 0.00	0.00 / 0.00	0.00 / 0.00	0.00 / 0.00	0.00 / 0.00	0.00 / 0.00	0.00 / 0.00	0.00 / 0.00
3	Tyr5	0.00 / 0.00	0.00 / 0.00	0.00 / 0.00	0.00 / 0.00	0.00 / 0.00	0.00 / 0.00	0.00 / 0.00	0.00 / 0.00	0.00 / 0.00	0.00 / 0.00	0.00 / 0.00	0.00 / 0.00	0.00 / 0.00	0.00 / 0.00	0.00 / 0.00	0.00 / 0.00	0.00 / 0.00	0.00 / 0.00	0.00 / 0.00	0.00 / 0.00	0.00 / 0.00	0.00 / 0.00	0.00 / 0.00	0.00 / 0.00	0.00 / 0.00	0.00 / 0.00	0.00 / 0.00	0.00 / 0.00	0.00 / 0.00	0.00 / 0.00	0.00 / 0.00	0.00 / 0.00	0.00 / 0.00	0.00 / 0.00	0.00 / 0.00	0.00 / 0.00	0.00 / 0.00	0.00 / 0.00	0.00 / 0.00	0.00 / 0.00
4	Leu 6	0.00 / 0.00	0.00 / 0.00	0.00 / 0.00	0.00 / 0.00	0.00 / 0.00	0.00 / 0.00	0.00 / 0.00	0.00 / 0.00	0.00 / 0.00	0.00 / 0.00	0.00 / 0.00	0.00 / 0.00	0.00 / 0.00	0.00 / 0.00	0.00 / 0.00	0.00 / 0.00	0.00 / 0.00	0.00 / 0.00	0.00 / 0.00	0.00 / 0.00	0.00 / 0.00	0.00 / 0.00	0.00 / 0.00	0.00 / 0.00	0.00 / 0.00	0.00 / 0.00	0.00 / 0.00	0.00 / 0.00	0.00 / 0.00	0.00 / 0.00	0.00 / 0.00	0.00 / 0.00	0.00 / 0.00	0.00 / 0.00	0.00 / 0.00	0.00 / 0.00	0.00 / 0.00	0.00 / 0.00	0.00 / 0.00	0.00 / 0.00
5	Asn 7	0.00 / 0.00	0.00 / 0.00	0.00 / 0.00	0.00 / 0.00	0.00 / 0.00	0.00 / 0.00	0.00 / 0.00	0.00 / 0.00	0.00 / 0.00	0.00 / 0.00	0.00 / 0.00	0.00 / 0.00	0.00 / 0.00	0.00 / 0.00	0.00 / 0.00	0.00 / 0.00	0.00 / 0.00	0.00 / 0.00	0.00 / 0.00	0.00 / 0.00	0.00 / 0.00	0.00 / 0.00	0.00 / 0.00	0.00 / 0.00	0.00 / 0.00	0.00 / 0.00	0.00 / 0.00	0.00 / 0.00	0.00 / 0.00	0.00 / 0.00	0.00 / 0.00	0.00 / 0.00	0.00 / 0.00	0.00 / 0.00	0.00 / 0.00	0.00 / 0.00	0.00 / 0.00	0.00 / 0.00	0.00 / 0.00	0.00 / 0.00
6	Val 8	0.00 / 0.00	0.00 / 0.00	0.00 / 0.00	0.00 / 0.00	0.00 / 0.00	0.00 / 0.00	0.00 / 0.00	0.00 / 0.00	0.00 / 0.00	0.00 / 0.00	0.00 / 0.00	0.00 / 0.00	0.00 / 0.00	0.00 / 0.00	0.00 / 0.00	0.00 / 0.00	0.00 / 0.00	0.00 / 0.00	0.00 / 0.00	0.00 / 0.00	0.00 / 0.00	0.00 / 0.00	0.00 / 0.00	0.00 / 0.00	0.00 / 0.00	0.00 / 0.00	0.00 / 0.00	0.00 / 0.00	0.00 / 0.00	0.00 / 0.00	0.00 / 0.00	0.00 / 0.00	0.00 / 0.00	0.00 / 0.00	0.00 / 0.00	0.00 / 0.00	0.00 / 0.00	0.00 / 0.00	0.00 / 0.00	0.00 / 0.00
7	Leu 9	0.00 / 0.00	0.00 / 0.00	0.00 / 0.00	0.00 / 0.00	0.00 / 0.00	0.00 / 0.00	0.00 / 0.00	0.00 / 0.00	0.00 / 0.00	0.00 / 0.00	0.00 / 0.00	0.00 / 0.00	0.00 / 0.00	0.00 / 0.00	0.00 / 0.00	0.00 / 0.00	0.00 / 0.00	0.00 / 0.00	0.00 / 0.00	0.00 / 0.00	0.00 / 0.00	0.00 / 0.00	0.00 / 0.00	0.00 / 0.00	0.00 / 0.00	0.00 / 0.00	0.00 / 0.00	0.00 / 0.00	0.00 / 0.00	0.00 / 0.00	0.00 / 0.00	0.00 / 0.00	0.00 / 0.00	0.00 / 0.00	0.00 / 0.00	0.00 / 0.00	0.00 / 0.00	0.00 / 0.00	0.00 / 0.00	0.00 / 0.00
8	Ala 10	0.00 / 0.00	0.00 / 0.00	0.00 / 0.00	0.00 / 0.00	0.00 / 0.00	0.00 / 0.00	0.00 / 0.00	0.00 / 0.00	0.00 / 0.00	0.00 / 0.00	0.00 / 0.00	0.00 / 0.00	0.00 / 0.00	0.00 / 0.00	0.00 / 0.00	0.00 / 0.00	0.00 / 0.00	0.00 / 0.00	0.00 / 0.00	0.00 / 0.00	0.00 / 0.00	0.00 / 0.00	0.00 / 0.00	0.00 / 0.00	0.00 / 0.00	0.00 / 0.00	0.00 / 0.00	0.00 / 0.00	0.00 / 0.00	0.00 / 0.00	0.00 / 0.00	0.00 / 0.00	0.00 / 0.00	0.00 / 0.00	0.00 / 0.00	0.00 / 0.00	0.00 / 0.00	0.00 / 0.00	0.00 / 0.00	0.00 / 0.00
9	Lys 11	0.00 / 0.00	0.00 / 0.00	0.00 / 0.00	0.00 / 0.00	0.00 / 0.00	0.00 / 0.00	0.00 / 0.00	0.00 / 0.00	0.00 / 0.00	0.00 / 0.00	0.00 / 0.00	0.00 / 0.00	0.00 / 0.00	0.00 / 0.00	0.00 / 0.00	0.00 / 0.00	0.00 / 0.00	0.00 / 0.00	0.00 / 0.00	0.00 / 0.00	0.00 / 0.00	0.00 / 0.00	0.00 / 0.00	0.00 / 0.00	0.00 / 0.00	0.00 / 0.00	0.00 / 0.00	0.00 / 0.00	0.00 / 0.00	0.00 / 0.00	0.00 / 0.00	0.00 / 0.00	0.00 / 0.00	0.00 / 0.00	0.00 / 0.00	0.00 / 0.00	0.00 / 0.00	0.00 / 0.00	0.00 / 0.00	0.00 / 0.00
10	Ala 12	0.00 / 0.00	0.00 / 0.00	0.00 / 0.00	0.00 / 0.00	0.00 / 0.00	0.00 / 0.00	0.00 / 0.00	0.00 / 0.00	0.00 / 0.00	0.00 / 0.00	0.00 / 0.00	0.00 / 0.00	0.00 / 0.00	0.00 / 0.00	0.00 / 0.00	0.00 / 0.00	0.00 / 0.00	0.00 / 0.00	0.00 / 0.00	0.00 / 0.00	0.00 / 0.00	0.00 / 0.00	0.00 / 0.00	0.00 / 0.00	0.00 / 0.00	0.00 / 0.00	0.00 / 0.00	0.00 / 0.00	0.00 / 0.00	0.00 / 0.00	0.00 / 0.00	0.00 / 0.00	0.00 / 0.00	0.00 / 0.00	0.00 / 0.00	0.00 / 0.00	0.00 / 0.00	0.00 / 0.00	0.00 / 0.00	0.00 / 0.00
11	Leu 13	0.00 / 0.00	0.00 / 0.00	0.12 / -0.00	0.74 / -0.01	[illegible]	[illegible]	[illegible]	[illegible]	[illegible]	[illegible]	[illegible]	[illegible]	[illegible]	[illegible]	[illegible]	[illegible]	[illegible]	[illegible]	[illegible]	[illegible]	[illegible]	[illegible]	[illegible]	[illegible]	[illegible]	[illegible]	[illegible]	[illegible]	[illegible]	[illegible]	[illegible]	[illegible]	[illegible]	[illegible]	[illegible]	[illegible]	[illegible]	[illegible]	[illegible]	[illegible]
12	Tyr 14	65.82 / 0.98	74.67 / 1.02	67.29 / 0.99	70.09 / 1.05	[illegible]	[illegible]	[illegible]	[illegible]	[illegible]	[illegible]	[illegible]	[illegible]	[illegible]	[illegible]	[illegible]	[illegible]	[illegible]	[illegible]	[illegible]	[illegible]	[illegible]	[illegible]	[illegible]	[illegible]	[illegible]	[illegible]	[illegible]	[illegible]	[illegible]	[illegible]	[illegible]	[illegible]	[illegible]	[illegible]	[illegible]	[illegible]	[illegible]	[illegible]	[illegible]	[illegible]
13	Asp 15	1.96 / -0.03	1.99 / -0.02	2.95 / -0.05	4.30 / -0.07	[illegible]	[illegible]	[illegible]	[illegible]	[illegible]	[illegible]	[illegible]	[illegible]	[illegible]	[illegible]	[illegible]	[illegible]	[illegible]	[illegible]	[illegible]	[illegible]	[illegible]	[illegible]	[illegible]	[illegible]	[illegible]	[illegible]	[illegible]	[illegible]	[illegible]	[illegible]	[illegible]	[illegible]	[illegible]	[illegible]	[illegible]	[illegible]	[illegible]	[illegible]	[illegible]	[illegible]
14	Asn 16	5.04 / -0.09	4.17 / -0.07	4.54 / -0.08	4.91 / -0.08	[illegible]	[illegible]	[illegible]	[illegible]	[illegible]	[illegible]	[illegible]	[illegible]	[illegible]	[illegible]	[illegible]	[illegible]	[illegible]	[illegible]	[illegible]	[illegible]	[illegible]	[illegible]	[illegible]	[illegible]	[illegible]	[illegible]	[illegible]	[illegible]	[illegible]	[illegible]	[illegible]	[illegible]	[illegible]	[illegible]	[illegible]	[illegible]	[illegible]	[illegible]	[illegible]	[illegible]
15	Val 17	3.31 / -0.04 / H	3.21 / -0.04 / H	3.58 / -0.04 / H	3.22 / -0.04 / H	[illegible]	[illegible]	[illegible]	[illegible]	[illegible]	[illegible]	[illegible]	[illegible]	[illegible]	[illegible]	[illegible]	[illegible]	[illegible]	[illegible]	[illegible]	[illegible]	[illegible]	[illegible]	[illegible]	[illegible]	[illegible]	[illegible]	[illegible]	[illegible]	[illegible]	[illegible]	[illegible]	[illegible]	[illegible]	[illegible]	[illegible]	[illegible]	[illegible]	[illegible]	[illegible]	[illegible]
16	Ala 18	0.00 / 0.00	0.00 / 0.00	0.00 / 0.00	0.00 / 0.00	0.00 / 0.00	0.00 / 0.00	0.00 / 0.00	0.00 / 0.00	0.00 / 0.00	0.00 / 0.00	0.00 / 0.00	0.00 / 0.00	0.00 / 0.00	0.00 / 0.00	0.00 / 0.00	0.00 / 0.00	0.00 / 0.00	0.00 / 0.00	0.00 / 0.00	0.00 / 0.00	0.00 / 0.00	0.00 / 0.00	0.00 / 0.00	0.00 / 0.00	0.00 / 0.00	0.00 / 0.00	0.00 / 0.00	0.00 / 0.00	0.00 / 0.00	0.00 / 0.00	0.00 / 0.00	0.00 / 0.00	0.00 / 0.00	0.00 / 0.00	0.00 / 0.00	0.00 / 0.00	0.00 / 0.00	0.00 / 0.00	0.00 / 0.00	0.00 / 0.00
17	Glu 19	24.96 / -0.26 / HS	26.21 / -0.28 / HS	24.86 / -0.24 / HS	25.53 / -0.26 / HS	[illegible]	[illegible]	[illegible]	[illegible]	[illegible]	[illegible]	[illegible]	[illegible]	[illegible]	[illegible]	[illegible]	[illegible]	[illegible]	[illegible]	[illegible]	[illegible]	[illegible]	[illegible]	[illegible]	[illegible]	[illegible]	[illegible]	[illegible]	[illegible]	[illegible]	[illegible]	[illegible]	[illegible]	[illegible]	[illegible]	[illegible]	[illegible]	[illegible]	[illegible]	[illegible]	[illegible]
18	Ser 20	21.87 / -0.15	22.24 / -0.16	19.65 / 0.22	19.15 / -0.09	[illegible]	[illegible]	[illegible]	[illegible]	[illegible]	[illegible]	[illegible]	[illegible]	[illegible]	[illegible]	[illegible]	[illegible]	[illegible]	[illegible]	[illegible]	[illegible]	[illegible]	[illegible]	[illegible]	[illegible]	[illegible]	[illegible]	[illegible]	[illegible]	[illegible]	[illegible]	[illegible]	[illegible]	[illegible]	[illegible]	[illegible]	[illegible]	[illegible]	[illegible]	[illegible]	[illegible]
19	Pro 21	5.86 / 0.09	0.00 / 0.00	0.00 / 0.00	0.00 / 0.00	[illegible]	[illegible]	[illegible]	[illegible]	[illegible]	[illegible]	[illegible]	[illegible]	[illegible]	[illegible]	[illegible]	[illegible]	[illegible]	[illegible]	[illegible]	[illegible]	[illegible]	[illegible]	[illegible]	[illegible]	[illegible]	[illegible]	[illegible]	[illegible]	[illegible]	[illegible]	[illegible]	[illegible]	[illegible]	[illegible]	[illegible]	[illegible]	[illegible]	[illegible]	[illegible]	[illegible]
20	Asp 22	12.64 / -0.14	0.00 / 0.00	0.00 / 0.00	0.00 / 0.00	[illegible]	[illegible]	[illegible]	[illegible]	[illegible]	[illegible]	[illegible]	[illegible]	[illegible]	[illegible]	[illegible]	[illegible]	[illegible]	[illegible]	[illegible]	[illegible]	[illegible]	[illegible]	[illegible]	[illegible]	[illegible]	[illegible]	[illegible]	[illegible]	[illegible]	[illegible]	[illegible]	[illegible]	[illegible]	[illegible]	[illegible]	[illegible]	[illegible]	[illegible]	[illegible]	[illegible]
21	Glu 23	8.13 / -0.09 / HS	6.30 / -0.11 / HS	8.10 / -0.09 / HS	8.22 / -0.15 / HS	[illegible]	[illegible]	[illegible]	[illegible]	[illegible]	[illegible]	[illegible]	[illegible]	[illegible]	[illegible]	[illegible]	[illegible]	[illegible]	[illegible]	[illegible]	[illegible]	[illegible]	[illegible]	[illegible]	[illegible]	[illegible]	[illegible]	[illegible]	[illegible]	[illegible]	[illegible]	[illegible]	[illegible]	[illegible]	[illegible]	[illegible]	[illegible]	[illegible]	[illegible]	[illegible]	[illegible]
22	Leu 24	0.00 / 0.00	0.00 / 0.00	0.00 / 0.00	0.00 / 0.00	0.00 / 0.00	0.00 / 0.00	0.00 / 0.00	0.00 / 0.00	0.00 / 0.00	0.00 / 0.00	0.00 / 0.00	0.00 / 0.00	0.00 / 0.00	0.00 / 0.00	0.00 / 0.00	0.00 / 0.00	0.00 / 0.00	0.00 / 0.00	0.00 / 0.00	0.00 / 0.00	0.00 / 0.00	0.00 / 0.00	0.00 / 0.00	0.00 / 0.00	0.00 / 0.00	0.00 / 0.00	0.00 / 0.00	0.00 / 0.00	0.00 / 0.00	0.00 / 0.00	0.00 / 0.00	0.00 / 0.00	0.00 / 0.00	0.00 / 0.00	0.00 / 0.00	0.00 / 0.00	0.00 / 0.00	0.00 / 0.00	0.00 / 0.00	0.00 / 0.00
23	Ser 25	0.00 / 0.00	0.00 / 0.00	0.00 / 0.00	0.00 / 0.00	0.00 / 0.00	0.00 / 0.00	0.00 / 0.00	0.00 / 0.00	0.00 / 0.00	0.00 / 0.00	0.00 / 0.00	0.00 / 0.00	0.00 / 0.00	0.00 / 0.00	0.00 / 0.00	0.00 / 0.00	0.00 / 0.00	0.00 / 0.00	0.00 / 0.00	0.00 / 0.00	0.00 / 0.00	0.00 / 0.00	0.00 / 0.00	0.00 / 0.00	0.00 / 0.00	0.00 / 0.00	0.00 / 0.00	0.00 / 0.00	0.00 / 0.00	0.00 / 0.00	0.00 / 0.00	0.00 / 0.00	0.00 / 0.00	0.00 / 0.00	0.00 / 0.00	0.00 / 0.00	0.00 / 0.00	0.00 / 0.00	0.00 / 0.00	0.00 / 0.00
24	Phe 26	0.00 / 0.00	0.00 / 0.00	0.00 / 0.00	0.00 / 0.00	0.00 / 0.00	0.00 / 0.00	0.00 / 0.00	0.00 / 0.00	0.00 / 0.00	0.00 / 0.00	0.00 / 0.00	0.00 / 0.00	0.00 / 0.00	0.00 / 0.00	0.00 / 0.00	0.00 / 0.00	0.00 / 0.00	0.00 / 0.00	0.00 / 0.00	0.00 / 0.00	0.00 / 0.00	0.00 / 0.00	0.00 / 0.00	0.00 / 0.00	0.00 / 0.00	0.00 / 0.00	0.00 / 0.00	0.00 / 0.00	0.00 / 0.00	0.00 / 0.00	0.00 / 0.00	0.00 / 0.00	0.00 / 0.00	0.00 / 0.00	0.00 / 0.00	0.00 / 0.00	0.00 / 0.00	0.00 / 0.00	0.00 / 0.00	0.00 / 0.00
25	Arg 27	0.00 / 0.00	0.00 / 0.00	0.00 / 0.00	0.00 / 0.00	[illegible]	[illegible]	[illegible]	[illegible]	[illegible]	[illegible]	[illegible]	[illegible]	[illegible]	[illegible]	[illegible]	[illegible]	[illegible]	[illegible]	[illegible]	[illegible]	[illegible]	[illegible]	[illegible]	[illegible]	[illegible]	[illegible]	[illegible]	[illegible]	[illegible]	[illegible]	[illegible]	[illegible]	[illegible]	[illegible]	[illegible]	[illegible]	[illegible]	[illegible]	[illegible]	[illegible]
26	Lys 28	40.99 / -1.07 / S	51.09 / -0.48 / HS	34.55 / -1.23 / HS	30.26 / -1.02 / HS	[illegible]	[illegible]	[illegible]	[illegible]	[illegible]	[illegible]	[illegible]	[illegible]	[illegible]	[illegible]	[illegible]	[illegible]	[illegible]	[illegible]	[illegible]	[illegible]	[illegible]	[illegible]	[illegible]	[illegible]	[illegible]	[illegible]	[illegible]	[illegible]	[illegible]	[illegible]	[illegible]	[illegible]	[illegible]	[illegible]	[illegible]	[illegible]	[illegible]	[illegible]	[illegible]	[illegible]
27	Gly 29	0.00 / 0.00	0.00 / 0.00	0.00 / 0.00	0.00 / 0.00	0.00 / 0.00	0.00 / 0.00	0.00 / 0.00	0.00 / 0.00	0.00 / 0.00	0.00 / 0.00	0.00 / 0.00	0.00 / 0.00	0.00 / 0.00	0.00 / 0.00	0.00 / 0.00	0.00 / 0.00	0.00 / 0.00	0.00 / 0.00	0.00 / 0.00	0.00 / 0.00	0.00 / 0.00	0.00 / 0.00	0.00 / 0.00	0.00 / 0.00	0.00 / 0.00	0.00 / 0.00	0.00 / 0.00	0.00 / 0.00	0.00 / 0.00	0.00 / 0.00	0.00 / 0.00	0.00 / 0.00	0.00 / 0.00	0.00 / 0.00	0.00 / 0.00	0.00 / 0.00	0.00 / 0.00	0.00 / 0.00	0.00 / 0.00	0.00 / 0.00
28	Asp 30	0.00 / 0.00	0.00 / 0.00	0.00 / 0.00	0.00 / 0.00	0.00 / 0.00	0.00 / 0.00	0.00 / 0.00	0.00 / 0.00	0.00 / 0.00	0.00 / 0.00	0.00 / 0.00	0.00 / 0.00	0.00 / 0.00	0.00 / 0.00	0.00 / 0.00	0.00 / 0.00	0.00 / 0.00	0.00 / 0.00	0.00 / 0.00	0.00 / 0.00	0.00 / 0.00	0.00 / 0.00	0.00 / 0.00	0.00 / 0.00	0.00 / 0.00	0.00 / 0.00	0.00 / 0.00	0.00 / 0.00	0.00 / 0.00	0.00 / 0.00	0.00 / 0.00	0.00 / 0.00	0.00 / 0.00	0.00 / 0.00	0.00 / 0.00	0.00 / 0.00	0.00 / 0.00	0.00 / 0.00	0.00 / 0.00	0.00 / 0.00
29	Ile 31	0.00 / 0.00	0.00 / 0.00	0.00 / 0.00	0.00 / 0.00	0.00 / 0.00	0.00 / 0.00	0.00 / 0.00	0.00 / 0.00	0.00 / 0.00	0.00 / 0.00	0.00 / 0.00	0.00 / 0.00	0.00 / 0.00	0.00 / 0.00	0.00 / 0.00	0.00 / 0.00	0.00 / 0.00	0.00 / 0.00	0.00 / 0.00	0.00 / 0.00	0.00 / 0.00	0.00 / 0.00	0.00 / 0.00	0.00 / 0.00	0.00 / 0.00	0.00 / 0.00	0.00 / 0.00	0.00 / 0.00	0.00 / 0.00	0.00 / 0.00	0.00 / 0.00	0.00 / 0.00	0.00 / 0.00	0.00 / 0.00	0.00 / 0.00	0.00 / 0.00	0.00 / 0.00	0.00 / 0.00	0.00 / 0.00	0.00 / 0.00
30	Met 32	0.00 / 0.00	0.00 / 0.00	0.00 / 0.00	0.00 / 0.00	0.00 / 0.00	0.00 / 0.00	0.00 / 0.00	0.00 / 0.00	0.00 / 0.00	0.00 / 0.00	0.00 / 0.00	0.00 / 0.00	0.00 / 0.00	0.00 / 0.00	0.00 / 0.00	0.00 / 0.00	0.00 / 0.00	0.00 / 0.00	0.00 / 0.00	0.00 / 0.00	0.00 / 0.00	0.00 / 0.00	0.00 / 0.00	0.00 / 0.00	0.00 / 0.00	0.00 / 0.00	0.00 / 0.00	0.00 / 0.00	0.00 / 0.00	0.00 / 0.00	0.00 / 0.00	0.00 / 0.00	0.00 / 0.00	0.00 / 0.00	0.00 / 0.00	0.00 / 0.00	0.00 / 0.00	0.00 / 0.00	0.00 / 0.00	0.00 / 0.00
31	Thr 33	0.00 / 0.00	0.00 / 0.00	0.00 / 0.00	0.00 / 0.00	0.00 / 0.00	0.00 / 0.00	0.00 / 0.00	0.00 / 0.00	0.00 / 0.00	0.00 / 0.00	0.00 / 0.00	0.00 / 0.00	0.00 / 0.00	0.00 / 0.00	0.00 / 0.00	0.00 / 0.00	0.00 / 0.00	0.00 / 0.00	0.00 / 0.00	0.00 / 0.00	0.00 / 0.00	0.00 / 0.00	0.00 / 0.00	0.00 / 0.00	0.00 / 0.00	0.00 / 0.00	0.00 / 0.00	0.00 / 0.00	0.00 / 0.00	0.00 / 0.00	0.00 / 0.00	0.00 / 0.00	0.00 / 0.00	0.00 / 0.00	0.00 / 0.00	0.00 / 0.00	0.00 / 0.00	0.00 / 0.00	0.00 / 0.00	0.00 / 0.00
32	Val 34	0.00 / 0.00	0.00 / 0.00	0.00 / 0.00	0.00 / 0.00	0.00 / 0.00	0.00 / 0.00	0.00 / 0.00	0.00 / 0.00	0.00 / 0.00	0.00 / 0.00	0.00 / 0.00	0.00 / 0.00	0.00 / 0.00	0.00 / 0.00	0.00 / 0.00	0.00 / 0.00	0.00 / 0.00	0.00 / 0.00	0.00 / 0.00	0.00 / 0.00	0.00 / 0.00	0.00 / 0.00	0.00 / 0.00	0.00 / 0.00	0.00 / 0.00	0.00 / 0.00	0.00 / 0.00	0.00 / 0.00	0.00 / 0.00	0.00 / 0.00	0.00 / 0.00	0.00 / 0.00	0.00 / 0.00	0.00 / 0.00	0.00 / 0.00	0.00 / 0.00	0.00 / 0.00	0.00 / 0.00	0.00 / 0.00	0.00 / 0.00
33	Leu 35	0.00 / 0.00	0.00 / 0.00	0.00 / 0.00	0.00 / 0.00	0.00 / 0.00	0.00 / 0.00	0.00 / 0.00	0.00 / 0.00	0.00 / 0.00	0.00 / 0.00	0.00 / 0.00	0.00 / 0.00	0.00 / 0.00	0.00 / 0.00	0.00 / 0.00	0.00 / 0.00	0.00 / 0.00	0.00 / 0.00	0.00 / 0.00	0.00 / 0.00	0.00 / 0.00	0.00 / 0.00	0.00 / 0.00	0.00 / 0.00	0.00 / 0.00	0.00 / 0.00	0.00 / 0.00	0.00 / 0.00	0.00 / 0.00	0.00 / 0.00	0.00 / 0.00	0.00 / 0.00	0.00 / 0.00	0.00 / 0.00	0.00 / 0.00	0.00 / 0.00	0.00 / 0.00	0.00 / 0.00	0.00 / 0.00	0.00 / 0.00
34	Glu 36	0.00 / 0.00	0.00 / 0.00	0.00 / 0.00	0.00 / 0.00	0.00 / 0.00	0.00 / 0.00	0.00 / 0.00	0.00 / 0.00	0.00 / 0.00	0.00 / 0.00	0.00 / 0.00	0.00 / 0.00	0.00 / 0.00	0.00 / 0.00	0.00 / 0.00	0.00 / 0.00	0.00 / 0.00	0.00 / 0.00	0.00 / 0.00	0.00 / 0.00	0.00 / 0.00	0.00 / 0.00	0.00 / 0.00	0.00 / 0.00	0.00 / 0.00	0.00 / 0.00	0.00 / 0.00	0.00 / 0.00	0.00 / 0.00	0.00 / 0.00	0.00 / 0.00	0.00 / 0.00	0.00 / 0.00	0.00 / 0.00	0.00 / 0.00	0.00 / 0.00	0.00 / 0.00	0.00 / 0.00	0.00 / 0.00	0.00 / 0.00
35	Arg 37	0.00 / 0.00	0.00 / 0.00	0.00 / 0.00	0.00 / 0.00	0.00 / 0.00	0.00 / 0.00	0.00 / 0.00	0.00 / 0.00	0.00 / 0.00	0.00 / 0.00	0.00 / 0.00	0.00 / 0.00	0.00 / 0.00	0.00 / 0.00	0.00 / 0.00	0.00 / 0.00	0.00 / 0.00	0.00 / 0.00	0.00 / 0.00	0.00 / 0.00	0.00 / 0.00	0.00 / 0.00	0.00 / 0.00	0.00 / 0.00	0.00 / 0.00	0.00 / 0.00	0.00 / 0.00	0.00 / 0.00	0.00 / 0.00	0.00 / 0.00	0.00 / 0.00	0.00 / 0.00	0.00 / 0.00	0.00 / 0.00	0.00 / 0.00	0.00 / 0.00	0.00 / 0.00	0.00 / 0.00	0.00 / 0.00	0.00 / 0.00
36	Asp 38	0.00 / 0.00	0.00 / 0.00	0.00 / 0.00	0.00 / 0.00	0.00 / 0.00	0.00 / 0.00	0.00 / 0.00	0.00 / 0.00	0.00 / 0.00	0.00 / 0.00	0.00 / 0.00	0.00 / 0.00	0.00 / 0.00	0.00 / 0.00	0.00 / 0.00	0.00 / 0.00	0.00 / 0.00	0.00 / 0.00	0.00 / 0.00	0.00 / 0.00	0.00 / 0.00	0.00 / 0.00	0.00 / 0.00	0.00 / 0.00	0.00 / 0.00	0.00 / 0.00	0.00 / 0.00	0.00 / 0.00	0.00 / 0.00	0.00 / 0.00	0.00 / 0.00	0.00 / 0.00	0.00 / 0.00	0.00 / 0.00	0.00 / 0.00	0.00 / 0.00	0.00 / 0.00	0.00 / 0.00	0.00 / 0.00	0.00 / 0.00
37	Thr 39	0.00 / 0.00	0.00 / 0.00	0.00 / 0.00	0.00 / 0.00	0.00 / 0.00	0.00 / 0.00	0.00 / 0.00	0.00 / 0.00	0.00 / 0.00	0.00 / 0.00	0.00 / 0.00	0.00 / 0.00	0.00 / 0.00	0.00 / 0.00	0.00 / 0.00	0.00 / 0.00	0.00 / 0.00	0.00 / 0.00	0.00 / 0.00	0.00 / 0.00	0.00 / 0.00	0.00 / 0.00	0.00 / 0.00	0.00 / 0.00	0.00 / 0.00	0.00 / 0.00	0.00 / 0.00	0.00 / 0.00	0.00 / 0.00	0.00 / 0.00	0.00 / 0.00	0.00 / 0.00	0.00 / 0.00	0.00 / 0.00	0.00 / 0.00	0.00 / 0.00	0.00 / 0.00	0.00 / 0.00	0.00 / 0.00	0.00 / 0.00
38	Gln 40	10.69 / -0.18	12.28 / -0.14	[illegible]	[illegible]	[illegible]	[illegible]	[illegible]	[illegible]	[illegible]	[illegible]	[illegible]	[illegible]	[illegible]	[illegible]	[illegible]	[illegible]	[illegible]	[illegible]	[illegible]	[illegible]	[illegible]	[illegible]	[illegible]	[illegible]	[illegible]	[illegible]	[illegible]	[illegible]	[illegible]	[illegible]	[illegible]	[illegible]	[illegible]	[illegible]	[illegible]	[illegible]	[illegible]	[illegible]	[illegible]	[illegible]
39	Gly 41	0.00 / 0.00	0.00 / 0.00	0.00 / 0.00	0.00 / 0.00	0.00 / 0.00	0.00 / 0.00	0.00 / 0.00	0.00 / 0.00	0.00 / 0.00	0.00 / 0.00	0.00 / 0.00	0.00 / 0.00	0.00 / 0.00	0.00 / 0.00	0.00 / 0.00	0.00 / 0.00	0.00 / 0.00	0.00 / 0.00	0.00 / 0.00	0.00 / 0.00	0.00 / 0.00	0.00 / 0.00	0.00 / 0.00	0.00 / 0.00	0.00 / 0.00	0.00 / 0.00	0.00 / 0.00	0.00 / 0.00	0.00 / 0.00	0.00 / 0.00	0.00 / 0.00	0.00 / 0.00	0.00 / 0.00	0.00 / 0.00	0.00 / 0.00	0.00 / 0.00	0.00 / 0.00	0.00 / 0.00	0.00 / 0.00	0.00 / 0.00
40	Leu 42	41.75 / 0.67	44.94 / 0.72	[illegible]	[illegible]	[illegible]	[illegible]	[illegible]	[illegible]	[illegible]	[illegible]	[illegible]	[illegible]	[illegible]	[illegible]	[illegible]	[illegible]	[illegible]	[illegible]	[illegible]	[illegible]	[illegible]	[illegible]	[illegible]	[illegible]	[illegible]	[illegible]	[illegible]	[illegible]	[illegible]	[illegible]	[illegible]	[illegible]	[illegible]	[illegible]	[illegible]	[illegible]	[illegible]	[illegible]	[illegible]	[illegible]
41	Asp 43	10.19 / -0.12	12.64 / -0.14	[illegible]	[illegible]	[illegible]	[illegible]	[illegible]	[illegible]	[illegible]	[illegible]	[illegible]	[illegible]	[illegible]	[illegible]	[illegible]	[illegible]	[illegible]	[illegible]	[illegible]	[illegible]	[illegible]	[illegible]	[illegible]	[illegible]	[illegible]	[illegible]	[illegible]	[illegible]	[illegible]	[illegible]	[illegible]	[illegible]	[illegible]	[illegible]	[illegible]	[illegible]	[illegible]	[illegible]	[illegible]	[illegible]
42	Gly 44	10.68 / 0.13	11.36 / 0.15	[illegible]	[illegible]	[illegible]	[illegible]	[illegible]	[illegible]	[illegible]	[illegible]	[illegible]	[illegible]	[illegible]	[illegible]	[illegible]	[illegible]	[illegible]	[illegible]	[illegible]	[illegible]	[illegible]	[illegible]	[illegible]	[illegible]	[illegible]	[illegible]	[illegible]	[illegible]	[illegible]	[illegible]	[illegible]	[illegible]	[illegible]	[illegible]	[illegible]	[illegible]	[illegible]	[illegible]	[illegible]	[illegible]
43	Trp 45	74.74 / 0.44	73.64 / 0.42	[illegible]	[illegible]	[illegible]	[illegible]	[illegible]	[illegible]	[illegible]	[illegible]	[illegible]	[illegible]	[illegible]	[illegible]	[illegible]	[illegible]	[illegible]	[illegible]	[illegible]	[illegible]	[illegible]	[illegible]	[illegible]	[illegible]	[illegible]	[illegible]	[illegible]	[illegible]	[illegible]	[illegible]	[illegible]	[illegible]	[illegible]	[illegible]	[illegible]	[illegible]	[illegible]	[illegible]	[illegible]	[illegible]
44	Trp 46	25.26 / 0.00	25.26 / 0.00	[illegible]	[illegible]	[illegible]	[illegible]	[illegible]	[illegible]	[illegible]	[illegible]	[illegible]	[illegible]	[illegible]	[illegible]	[illegible]	[illegible]	[illegible]	[illegible]	[illegible]	[illegible]	[illegible]	[illegible]	[illegible]	[illegible]	[illegible]	[illegible]	[illegible]	[illegible]	[illegible]	[illegible]	[illegible]	[illegible]	[illegible]	[illegible]	[illegible]	[illegible]	[illegible]	[illegible]	[illegible]	[illegible]
45	Leu 47	0.00 / 0.00	0.00 / 0.00	0.00 / 0.00	0.00 / 0.00	0.00 / 0.00	0.00 / 0.00	0.00 / 0.00	0.00 / 0.00	0.00 / 0.00	0.00 / 0.00	0.00 / 0.00	0.00 / 0.00	0.00 / 0.00	0.00 / 0.00	0.00 / 0.00	0.00 / 0.00	0.00 / 0.00	0.00 / 0.00	0.00 / 0.00	0.00 / 0.00	0.00 / 0.00	0.00 / 0.00	0.00 / 0.00	0.00 / 0.00	0.00 / 0.00	0.00 / 0.00	0.00 / 0.00	0.00 / 0.00	0.00 / 0.00	0.00 / 0.00	0.00 / 0.00	0.00 / 0.00	0.00 / 0.00	0.00 / 0.00	0.00 / 0.00	0.00 / 0.00	0.00 / 0.00	0.00 / 0.00	0.00 / 0.00	0.00 / 0.00
46	Cys 48	0.00 / 0.00	0.00 / 0.00	0.00 / 0.00	0.00 / 0.00	0.00 / 0.00	0.00 / 0.00	0.00 / 0.00	0.00 / 0.00	0.00 / 0.00	0.00 / 0.00	0.00 / 0.00	0.00 / 0.00	0.00 / 0.00	0.00 / 0.00	0.00 / 0.00	0.00 / 0.00	0.00 / 0.00	0.00 / 0.00	0.00 / 0.00	0.00 / 0.00	0.00 / 0.00	0.00 / 0.00	0.00 / 0.00	0.00 / 0.00	0.00 / 0.00	0.00 / 0.00	0.00 / 0.00	0.00 / 0.00	0.00 / 0.00	0.00 / 0.00	0.00 / 0.00	0.00 / 0.00	0.00 / 0.00	0.00 / 0.00	0.00 / 0.00	0.00 / 0.00	0.00 / 0.00	0.00 / 0.00	0.00 / 0.00	0.00 / 0.00
47	Ser 49	0.00 / 0.00	0.00 / 0.00	0.00 / 0.00	0.00 / 0.00	0.00 / 0.00	0.00 / 0.00	0.00 / 0.00	0.00 / 0.00	0.00 / 0.00	0.00 / 0.00	0.00 / 0.00	0.00 / 0.00	0.00 / 0.00	0.00 / 0.00	0.00 / 0.00	0.00 / 0.00	0.00 / 0.00	0.00 / 0.00	0.00 / 0.00	0.00 / 0.00	0.00 / 0.00	0.00 / 0.00	0.00 / 0.00	0.00 / 0.00	0.00 / 0.00	0.00 / 0.00	0.00 / 0.00	0.00 / 0.00	0.00 / 0.00	0.00 / 0.00	0.00 / 0.00	0.00 / 0.00	0.00 / 0.00	0.00 / 0.00	0.00 / 0.00	0.00 / 0.00	0.00 / 0.00	0.00 / 0.00	0.00 / 0.00	0.00 / 0.00
48	Leu 50	0.00 / 0.00	0.00 / 0.00	0.00 / 0.00	0.00 / 0.00	0.00 / 0.00	0.00 / 0.00	0.00 / 0.00	0.00 / 0.00	0.00 / 0.00	0.00 / 0.00	0.00 / 0.00	0.00 / 0.00	0.00 / 0.00	0.00 / 0.00	0.00 / 0.00	0.00 / 0.00	0.00 / 0.00	0.00 / 0.00	0.00 / 0.00	0.00 / 0.00	0.00 / 0.00	0.00 / 0.00	0.00 / 0.00	0.00 / 0.00	0.00 / 0.00	0.00 / 0.00	0.00 / 0.00	0.00 / 0.00	0.00 / 0.00	0.00 / 0.00	0.00 / 0.00	0.00 / 0.00	0.00 / 0.00	0.00 / 0.00	0.00 / 0.00	0.00 / 0.00	0.00 / 0.00	0.00 / 0.00	0.00 / 0.00	0.00 / 0.00
49	His 51	0.00 / 0.00	0.00 / 0.00	0.00 / 0.00	0.00 / 0.00	0.00 / 0.00	0.00 / 0.00	0.00 / 0.00	0.00 / 0.00	0.00 / 0.00	0.00 / 0.00	0.00 / 0.00	0.00 / 0.00	0.00 / 0.00	0.00 / 0.00	0.00 / 0.00	0.00 / 0.00	0.00 / 0.00	0.00 / 0.00	0.00 / 0.00	0.00 / 0.00	0.00 / 0.00	0.00 / 0.00	0.00 / 0.00	0.00 / 0.00	0.00 / 0.00	0.00 / 0.00	0.00 / 0.00	0.00 / 0.00	0.00 / 0.00	0.00 / 0.00	0.00 / 0.00	0.00 / 0.00	0.00 / 0.00	0.00 / 0.00	0.00 / 0.00	0.00 / 0.00	0.00 / 0.00	0.00 / 0.00	0.00 / 0.00	0.00 / 0.00
50	Gly 52	0.00 / 0.00	0.00 / 0.00	0.00 / 0.00	0.00 / 0.00	0.00 / 0.00	0.00 / 0.00	0.00 / 0.00	0.00 / 0.00	0.00 / 0.00	0.00 / 0.00	0.00 / 0.00	0.00 / 0.00	0.00 / 0.00	0.00 / 0.00	0.00 / 0.00	0.00 / 0.00	0.00 / 0.00	0.00 / 0.00	0.00 / 0.00	0.00 / 0.00	0.00 / 0.00	0.00 / 0.00	0.00 / 0.00	0.00 / 0.00	0.00 / 0.00	0.00 / 0.00	0.00 / 0.00	0.00 / 0.00	0.00 / 0.00	0.00 / 0.00	0.00 / 0.00	0.00 / 0.00	0.00 / 0.00	0.00 / 0.00	0.00 / 0.00	0.00 / 0.00	0.00 / 0.00	0.00 / 0.00	0.00 / 0.00	0.00 / 0.00
51	Arg 53	0.00 / 0.00	0.00 / 0.00	0.00 / 0.00	0.00 / 0.00	0.00 / 0.00	0.00 / 0.00	0.00 / 0.00	0.00 / 0.00	0.00 / 0.00	0.00 / 0.00	0.00 / 0.00	0.00 / 0.00	0.00 / 0.00	0.00 / 0.00	0.00 / 0.00	0.00 / 0.00	0.00 / 0.00	0.00 / 0.00	0.00 / 0.00	0.00 / 0.00	0.00 / 0.00	0.00 / 0.00	0.00 / 0.00	0.00 / 0.00	0.00 / 0.00	0.00 / 0.00	0.00 / 0.00	0.00 / 0.00	0.00 / 0.00	0.00 / 0.00	0.00 / 0.00	0.00 / 0.00	0.00 / 0.00	0.00 / 0.00	0.00 / 0.00	0.00 / 0.00	0.00 / 0.00	0.00 / 0.00	0.00 / 0.00	0.00 / 0.00
52	Gln 54	0.00 / 0.00	0.00 / 0.00	0.00 / 0.00	0.00 / 0.00	0.00 / 0.00	0.00 / 0.00	0.00 / 0.00	0.00 / 0.00	0.00 / 0.00	0.00 / 0.00	0.00 / 0.00	0.00 / 0.00	0.00 / 0.00	0.00 / 0.00	0.00 / 0.00	0.00 / 0.00	0.00 / 0.00	0.00 / 0.00	0.00 / 0.00	0.00 / 0.00	0.00 / 0.00	0.00 / 0.00	0.00 / 0.00	0.00 / 0.00	0.00 / 0.00	0.00 / 0.00	0.00 / 0.00	0.00 / 0.00	0.00 / 0.00	0.00 / 0.00	0.00 / 0.00	0.00 / 0.00	0.00 / 0.00	0.00 / 0.00	0.00 / 0.00	0.00 / 0.00	0.00 / 0.00	0.00 / 0.00	0.00 / 0.00	0.00 / 0.00
53	Gly 55	0.00 / 0.00	0.00 / 0.00	0.00 / 0.00	0.00 / 0.00	0.00 / 0.00	0.00 / 0.00	0.00 / 0.00	0.00 / 0.00	0.00 / 0.00	0.00 / 0.00	0.00 / 0.00	0.00 / 0.00	0.00 / 0.00	0.00 / 0.00	0.00 / 0.00	0.00 / 0.00	0.00 / 0.00	0.00 / 0.00	0.00 / 0.00	0.00 / 0.00	0.00 / 0.00	0.00 / 0.00	0.00 / 0.00	0.00 / 0.00	0.00 / 0.00	0.00 / 0.00	0.00 / 0.00	0.00 / 0.00	0.00 / 0.00	0.00 / 0.00	0.00 / 0.00	0.00 / 0.00	0.00 / 0.00	0.00 / 0.00	0.00 / 0.00	0.00 / 0.00	0.00 / 0.00	0.00 / 0.00	0.00 / 0.00	0.00 / 0.00
54	Ile 56	9.04 / 0.14	9.71 / 0.16	[illegible]	[illegible]	[illegible]	[illegible]	[illegible]	[illegible]	[illegible]	[illegible]	[illegible]	[illegible]	[illegible]	[illegible]	[illegible]	[illegible]	[illegible]	[illegible]	[illegible]	[illegible]	[illegible]	[illegible]	[illegible]	[illegible]	[illegible]	[illegible]	[illegible]	[illegible]	[illegible]	[illegible]	[illegible]	[illegible]	[illegible]	[illegible]	[illegible]	[illegible]	[illegible]	[illegible]	[illegible]	[illegible]
55	Val 57	0.00 / 0.00	0.00 / 0.00	0.00 / 0.00	0.00 / 0.00	0.00 / 0.00	0.00 / 0.00	0.00 / 0.00	0.00 / 0.00	0.00 / 0.00	0.00 / 0.00	0.00 / 0.00	0.00 / 0.00	0.00 / 0.00	0.00 / 0.00	0.00 / 0.00	0.00 / 0.00	0.00 / 0.00	0.00 / 0.00	0.00 / 0.00	0.00 / 0.00	0.00 / 0.00	0.00 / 0.00	0.00 / 0.00	0.00 / 0.00	0.00 / 0.00	0.00 / 0.00	0.00 / 0.00	0.00 / 0.00	0.00 / 0.00	0.00 / 0.00	0.00 / 0.00	0.00 / 0.00	0.00 / 0.00	0.00 / 0.00	0.00 / 0.00	0.00 / 0.00	0.00 / 0.00	0.00 / 0.00	0.00 / 0.00	0.00 / 0.00
56	Pro 58	21.76 / 0.35	23.26 / 0.37	[illegible]	[illegible]	[illegible]	[illegible]	[illegible]	[illegible]	[illegible]	[illegible]	[illegible]	[illegible]	[illegible]	[illegible]	[illegible]	[illegible]	[illegible]	[illegible]	[illegible]	[illegible]	[illegible]	[illegible]	[illegible]	[illegible]	[illegible]	[illegible]	[illegible]	[illegible]	[illegible]	[illegible]	[illegible]	[illegible]	[illegible]	[illegible]	[illegible]	[illegible]	[illegible]	[illegible]	[illegible]	[illegible]
57	Gly 59	0.44 / -0.00 -0.00	0.29 / -0.00 -0.00	[illegible]	[illegible]	[illegible]	[illegible]	[illegible]	[illegible]	[illegible]	[illegible]	[illegible]	[illegible]	[illegible]	[illegible]	[illegible]	[illegible]	[illegible]	[illegible]	[illegible]	[illegible]	[illegible]	[illegible]	[illegible]	[illegible]	[illegible]	[illegible]	[illegible]	[illegible]	[illegible]	[illegible]	[illegible]	[illegible]	[illegible]	[illegible]	[illegible]	[illegible]	[illegible]	[illegible]	[illegible]	[illegible]
58	Asn 60	55.22 / -0.47 / H	57.75 / -0.31 / H	[illegible]	[illegible]	[illegible]	[illegible]	[illegible]	[illegible]	[illegible]	[illegible]	[illegible]	[illegible]	[illegible]	[illegible]	[illegible]	[illegible]	[illegible]	[illegible]	[illegible]	[illegible]	[illegible]	[illegible]	[illegible]	[illegible]	[illegible]	[illegible]	[illegible]	[illegible]	[illegible]	[illegible]	[illegible]	[illegible]	[illegible]	[illegible]	[illegible]	[illegible]	[illegible]	[illegible]	[illegible]	[illegible]
59	Arg 61	63.35 / -1.37 / H	66.28 / -1.28 / H	[illegible]	[illegible]	[illegible]	[illegible]	[illegible]	[illegible]	[illegible]	[illegible]	[illegible]	[illegible]	[illegible]	[illegible]	[illegible]	[illegible]	[illegible]	[illegible]	[illegible]	[illegible]	[illegible]	[illegible]	[illegible]	[illegible]	[illegible]	[illegible]	[illegible]	[illegible]	[illegible]	[illegible]	[illegible]	[illegible]	[illegible]	[illegible]	[illegible]	[illegible]	[illegible]	[illegible]	[illegible]	[illegible]
60	Leu 62	0.00 / 0.00	0.00 / 0.00	0.00 / 0.00	0.00 / 0.00	0.00 / 0.00	0.00 / 0.00	0.00 / 0.00	0.00 / 0.00	0.00 / 0.00	0.00 / 0.00	0.00 / 0.00	0.00 / 0.00	0.00 / 0.00	0.00 / 0.00	0.00 / 0.00	0.00 / 0.00	0.00 / 0.00	0.00 / 0.00	0.00 / 0.00	0.00 / 0.00	0.00 / 0.00	0.00 / 0.00	0.00 / 0.00	0.00 / 0.00	0.00 / 0.00	0.00 / 0.00	0.00 / 0.00	0.00 / 0.00	0.00 / 0.00	0.00 / 0.00	0.00 / 0.00	0.00 / 0.00	0.00 / 0.00	0.00 / 0.00	0.00 / 0.00	0.00 / 0.00	0.00 / 0.00	0.00 / 0.00	0.00 / 0.00	0.00 / 0.00
61	Lys 63	0.00 / 0.00	0.00 / 0.00	0.00 / 0.00	0.00 / 0.00	0.00 / 0.00	0.00 / 0.00	0.00 / 0.00	0.00 / 0.00	0.00 / 0.00	0.00 / 0.00	0.00 / 0.00	0.00 / 0.00	0.00 / 0.00	0.00 / 0.00	0.00 / 0.00	0.00 / 0.00	0.00 / 0.00	0.00 / 0.00	0.00 / 0.00	0.00 / 0.00	0.00 / 0.00	0.00 / 0.00	0.00 / 0.00	0.00 / 0.00	0.00 / 0.00	0.00 / 0.00	0.00 / 0.00	0.00 / 0.00	0.00 / 0.00	0.00 / 0.00	0.00 / 0.00	0.00 / 0.00	0.00 / 0.00	0.00 / 0.00	0.00 / 0.00	0.00 / 0.00	0.00 / 0.00	0.00 / 0.00	0.00 / 0.00	0.00 / 0.00
62	Ile 64	0.00 / 0.00	0.00 / 0.00	0.00 / 0.00	0.00 / 0.00	0.00 / 0.00	0.00 / 0.00	0.00 / 0.00	0.00 / 0.00	0.00 / 0.00	0.00 / 0.00	0.00 / 0.00	0.00 / 0.00	0.00 / 0.00	0.00 / 0.00	0.00 / 0.00	0.00 / 0.00	0.00 / 0.00	0.00 / 0.00	0.00 / 0.00	0.00 / 0.00	0.00 / 0.00	0.00 / 0.00	0.00 / 0.00	0.00 / 0.00	0.00 / 0.00	0.00 / 0.00	0.00 / 0.00	0.00 / 0.00	0.00 / 0.00	0.00 / 0.00	0.00 / 0.00	0.00 / 0.00	0.00 / 0.00	0.00 / 0.00	0.00 / 0.00	0.00 / 0.00	0.00 / 0.00	0.00 / 0.00	0.00 / 0.00	0.00 / 0.00
63	Leu 65	0.00 / 0.00	0.00 / 0.00	0.00 / 0.00	0.00 / 0.00	0.00 / 0.00	0.00 / 0.00	0.00 / 0.00	0.00 / 0.00	0.00 / 0.00	0.00 / 0.00	0.00 / 0.00	0.00 / 0.00	0.00 / 0.00	0.00 / 0.00	0.00 / 0.00	0.00 / 0.00	0.00 / 0.00	0.00 / 0.00	0.00 / 0.00	0.00 / 0.00	0.00 / 0.00	0.00 / 0.00	0.00 / 0.00	0.00 / 0.00	0.00 / 0.00	0.00 / 0.00	0.00 / 0.00	0.00 / 0.00	0.00 / 0.00	0.00 / 0.00	0.00 / 0.00	0.00 / 0.00	0.00 / 0.00	0.00 / 0.00	0.00 / 0.00	0.00 / 0.00	0.00 / 0.00	0.00 / 0.00	0.00 / 0.00	0.00 / 0.00
64	Val 66	0.00 / 0.00	0.00 / 0.00	0.00 / 0.00	0.00 / 0.00	0.00 / 0.00	0.00 / 0.00	0.00 / 0.00	0.00 / 0.00	0.00 / 0.00	0.00 / 0.00	0.00 / 0.00	0.00 / 0.00	0.00 / 0.00	0.00 / 0.00	0.00 / 0.00	0.00 / 0.00	0.00 / 0.00	0.00 / 0.00	0.00 / 0.00	0.00 / 0.00	0.00 / 0.00	0.00 / 0.00	0.00 / 0.00	0.00 / 0.00	0.00 / 0.00	0.00 / 0.00	0.00 / 0.00	0.00 / 0.00	0.00 / 0.00	0.00 / 0.00	0.00 / 0.00	0.00 / 0.00	0.00 / 0.00	0.00 / 0.00	0.00 / 0.00	0.00 / 0.00	0.00 / 0.00	0.00 / 0.00	0.00 / 0.00	0.00 / 0.00
65	Gly 67	0.00 / 0.00	0.00 / 0.00	0.00 / 0.00	0.00 / 0.00	0.00 / 0.00	0.00 / 0.00	0.00 / 0.00	0.00 / 0.00	0.00 / 0.00	0.00 / 0.00	0.00 / 0.00	0.00 / 0.00	0.00 / 0.00	0.00 / 0.00	0.00 / 0.00	0.00 / 0.00	0.00 / 0.00	0.00 / 0.00	0.00 / 0.00	0.00 / 0.00	0.00 / 0.00	0.00 / 0.00	0.00 / 0.00	0.00 / 0.00	0.00 / 0.00	0.00 / 0.00	0.00 / 0.00	0.00 / 0.00	0.00 / 0.00	0.00 / 0.00	0.00 / 0.00	0.00 / 0.00	0.00 / 0.00	0.00 / 0.00	0.00 / 0.00	0.00 / 0.00	0.00 / 0.00	0.00 / 0.00	0.00 / 0.00	0.00 / 0.00
66	Met 68	0.00 / 0.00	0.00 / 0.00	0.00 / 0.00	0.00 / 0.00	0.00 / 0.00	0.00 / 0.00	0.00 / 0.00	0.00 / 0.00	0.00 / 0.00	0.00 / 0.00	0.00 / 0.00	0.00 / 0.00	0.00 / 0.00	0.00 / 0.00	0.00 / 0.00	0.00 / 0.00	0.00 / 0.00	0.00 / 0.00	0.00 / 0.00	0.00 / 0.00	0.00 / 0.00	0.00 / 0.00	0.00 / 0.00	0.00 / 0.00	0.00 / 0.00	0.00 / 0.00	0.00 / 0.00	0.00 / 0.00	0.00 / 0.00	0.00 / 0.00	0.00 / 0.00	0.00 / 0.00	0.00 / 0.00	0.00 / 0.00	0.00 / 0.00	0.00 / 0.00	0.00 / 0.00	0.00 / 0.00	0.00 / 0.00	0.00 / 0.00
67	Tyr 69	0.00 / 0.00	0.00 / 0.00	0.00 / 0.00	0.00 / 0.00	0.00 / 0.00	0.00 / 0.00	0.00 / 0.00	0.00 / 0.00	0.00 / 0.00	0.00 / 0.00	0.00 / 0.00	0.00 / 0.00	0.00 / 0.00	0.00 / 0.00	0.00 / 0.00	0.00 / 0.00	0.00 / 0.00	0.00 / 0.00	0.00 / 0.00	0.00 / 0.00	0.00 / 0.00	0.00 / 0.00	0.00 / 0.00	0.00 / 0.00	0.00 / 0.00	0.00 / 0.00	0.00 / 0.00	0.00 / 0.00	0.00 / 0.00	0.00 / 0.00	0.00 / 0.00	0.00 / 0.00	0.00 / 0.00	0.00 / 0.00	0.00 / 0.00	0.00 / 0.00	0.00 / 0.00	0.00 / 0.00	0.00 / 0.00	0.00 / 0.00
68	Asp 70	0.00 / 0.00	0.00 / 0.00	0.00 / 0.00	0.00 / 0.00	0.00 / 0.00	0.00 / 0.00	0.00 / 0.00	0.00 / 0.00	0.00 / 0.00	0.00 / 0.00	0.00 / 0.00	0.00 / 0.00	0.00 / 0.00	0.00 / 0.00	0.00 / 0.00	0.00 / 0.00	0.00 / 0.00	0.00 / 0.00	0.00 / 0.00	0.00 / 0.00	0.00 / 0.00	0.00 / 0.00	0.00 / 0.00	0.00 / 0.00	0.00 / 0.00	0.00 / 0.00	0.00 / 0.00	0.00 / 0.00	0.00 / 0.00	0.00 / 0.00	0.00 / 0.00	0.00 / 0.00	0.00 / 0.00	0.00 / 0.00	0.00 / 0.00	0.00 / 0.00	0.00 / 0.00	0.00 / 0.00	0.00 / 0.00	0.00 / 0.00
69	Lys 71	0.00 / 0.00	0.00 / 0.00	0.00 / 0.00	0.00 / 0.00	0.00 / 0.00	0.00 / 0.00	0.00 / 0.00	0.00 / 0.00	0.00 / 0.00	0.00 / 0.00	0.00 / 0.00	0.00 / 0.00	0.00 / 0.00	0.00 / 0.00	0.00 / 0.00	0.00 / 0.00	0.00 / 0.00	0.00 / 0.00	0.00 / 0.00	0.00 / 0.00	0.00 / 0.00	0.00 / 0.00	0.00 / 0.00	0.00 / 0.00	0.00 / 0.00	0.00 / 0.00	0.00 / 0.00	0.00 / 0.00	0.00 / 0.00	0.00 / 0.00	0.00 / 0.00	0.00 / 0.00	0.00 / 0.00	0.00 / 0.00	0.00 / 0.00	0.00 / 0.00	0.00 / 0.00	0.00 / 0.00	0.00 / 0.00	0.00 / 0.00
70	Lys 72	0.00 / 0.00	0.00 / 0.00	0.00 / 0.00	0.00 / 0.00	0.00 / 0.00	0.00 / 0.00	0.00 / 0.00	0.00 / 0.00	0.00 / 0.00	0.00 / 0.00	0.00 / 0.00	0.00 / 0.00	0.00 / 0.00	0.00 / 0.00	0.00 / 0.00	0.00 / 0.00	0.00 / 0.00	0.00 / 0.00	0.00 / 0.00	0.00 / 0.00	0.00 / 0.00	0.00 / 0.00	0.00 / 0.00	0.00 / 0.00	0.00 / 0.00	0.00 / 0.00	0.00 / 0.00	0.00 / 0.00	0.00 / 0.00	0.00 / 0.00	0.00 / 0.00	0.00 / 0.00	0.00 / 0.00	0.00 / 0.00	0.00 / 0.00	0.00 / 0.00	0.00 / 0.00	0.00 / 0.00	0.00 / 0.00	0.00 / 0.00
71	Pro 73	0.00 / 0.00	0.00 / 0.00	0.00 / 0.00	0.00 / 0.00	0.00 / 0.00	0.00 / 0.00	0.00 / 0.00	0.00 / 0.00	0.00 / 0.00	0.00 / 0.00	0.00 / 0.00	0.00 / 0.00	0.00 / 0.00	0.00 / 0.00	0.00 / 0.00	0.00 / 0.00	0.00 / 0.00	0.00 / 0.00	0.00 / 0.00	0.00 / 0.00	0.00 / 0.00	0.00 / 0.00	0.00 / 0.00	0.00 / 0.00	0.00 / 0.00	0.00 / 0.00	0.00 / 0.00	0.00 / 0.00	0.00 / 0.00	0.00 / 0.00	0.00 / 0.00	0.00 / 0.00	0.00 / 0.00	0.00 / 0.00	0.00 / 0.00	0.00 / 0.00	0.00 / 0.00	0.00 / 0.00	0.00 / 0.00	0.00 / 0.00
72	Ala 74	0.00 / 0.00	0.00 / 0.00	0.00 / 0.00	0.00 / 0.00	0.00 / 0.00	0.00 / 0.00	0.00 / 0.00	0.00 / 0.00	0.00 / 0.00	0.00 / 0.00	0.00 / 0.00	0.00 / 0.00	0.00 / 0.00	0.00 / 0.00	0.00 / 0.00	0.00 / 0.00	0.00 / 0.00	0.00 / 0.00	0.00 / 0.00	0.00 / 0.00	0.00 / 0.00	0.00 / 0.00	0.00 / 0.00	0.00 / 0.00	0.00 / 0.00	0.00 / 0.00	0.00 / 0.00	0.00 / 0.00	0.00 / 0.00	0.00 / 0.00	0.00 / 0.00	0.00 / 0.00	0.00 / 0.00	0.00 / 0.00	0.00 / 0.00	0.00 / 0.00	0.00 / 0.00	0.00 / 0.00	0.00 / 0.00	0.00 / 0.00

Supplementary table S5. Interface analysis of CAS SH3-Vinculin (SH3 part).
Hydrogen bonds and nonpolar contacts between Cas SH3 and its ligands were analyzed with PISA with default settings. Prior to analysis, the linker sequence (residues 75-82) was removed from the chimeric proteins. Solvent accessible residues are colored yellow and inaccessible residues are blue. ΔG represents free energy of dissociation (solvation energy effect, kcal/mol). BSA means Buried Surface Area (Å²) and is displayed with bars, one bar per 10%. HSDC indicates residues that contain the across-interface hydrogen bond, salt bridge, or disulfide bond atoms. The corresponding table cells have a brown background and contain letter H for a hydrogen bond, S for a salt bridge, and HS for a combination of hydrogen bond and salt bridge.
http://www.ebi.ac.uk/msd-srv/prot_int/pistart.html

(CAS SH3)-Vinculin structure number — BSA// ΔiG /HSDC

position	Interface residue	01 Vcl	02 Vcl	03 Vcl	04 Vcl	05 Vcl	06 Vcl	07 Vcl	08 Vcl	09 Vcl	10 Vcl
-6	LEU 83	0.00 / 0.00	19.58 / 0.30	0.00 / 0.00	0.00 / 0.00	51.71 / 0.83	0.00 / 0.00	0.00 / 0.00	11.89 / 0.19	0.00 / 0.00	0.00 / 0.00
-5	THR 84	0.00 / 0.00	0.00 / 0.00	0.00 / 0.00	0.00 / 0.00	0.00 / 0.00	0.00 / 0.00	6.69 / 0.11	0.00 / 0.00	0.00 / 0.00	0.00 / 0.00
-4	ASP 85	0.00 / 0.00	0.00 / 0.00	0.00 / 0.00	0.00 / 0.00	0.00 / 0.00	0.00 / 0.00	0.00 / 0.00	0.00 / 0.00	0.00 / 0.00	0.00 / 0.00
-3	**GLU 86**	62.99 / -0.29 S	45.22 / -0.24 HS	47.54 / -0.15 HS	62.39 / -0.43 HS	28.96 / -0.21 HS	48.78 / -0.10 HS	44.53 / -0.13 HS	52.57 / 0.16 HS	52.23 / -0.11 HS	67.29 / -0.27 HS
-2	LEU 87	8.02 / -0.09	8.02 / -0.05	8.02 / -0.09	8.02 / -0.04	8.02 / -0.07	8.02 / -0.07	8.02 / -0.04	8.02 / -0.07	8.02 / -0.06	8.02 / -0.06
-1	**ALA 88**	51.01 / 0.71	49.59 / 0.71	51.88 / 0.71	54.55 / 0.71	48.00 / 0.71	49.60 / 0.71	53.04 / 0.71	53.99 / 0.71	49.60 / 0.71	52.58 / 0.71
0	**PRO 89**	76.75 / 0.87 H	78.14 / 0.87 H	79.81 / 0.91 H	86.40 / 0.96 H	81.58 / 0.90 H	77.83 / 0.89 H	77.42 / 0.90 H	80.52 / 0.87 H	81.25 / 0.96 H	73.43 / 0.84 H
1	PRO 90	18.08 / -0.08 H	25.55 / -0.08 H	17.65 / -0.08 H	20.91 / -0.09	26.66 / -0.04 H	25.67 / -0.03 H	23.65 / -0.09 H	24.31 / -0.06 H	21.73 / -0.06	17.66 / -0.08 H
2	**LYS 91**	122.64 / -0.70 HS	128.59 / -0.59 HS	126.02 / -0.65 HS	123.19 / -0.62 HS	125.87 / -0.64 HS	128.05 / -0.61 HS	129.77 / -0.57 HS	128.75 / -0.55 HS	129.66 / -0.53 HS	136.92 / -0.45 HS
3	**PRO 92**	77.91 / 1.16	82.32 / 1.21	79.01 / 1.17	79.13 / 1.17	82.14 / 1.22	79.13 / 1.17	76.13 / 1.13	79.41 / 1.15	76.68 / 1.11	78.69 / 1.16
4	PRO 93	6.12 / -0.07	6.39 / -0.07	5.52 / -0.06	6.61 / -0.08	6.72 / -0.07	5.76 / -0.07	6.01 / -0.07	6.00 / -0.07	6.01 / -0.07	6.37 / -0.07
5	**LEU 94**	90.84 / 1.45	95.51 / 1.53	90.50 / 1.45	93.67 / 1.50	92.84 / 1.49	97.55 / 1.56	94.86 / 1.52	89.34 / 1.43	94.02 / 1.50	92.53 / 1.48
6	PRO 95	21.20 / 0.34	18.26 / 0.29	20.69 / 0.33	20.69 / 0.33	20.20 / 0.32	20.12 / 0.32	19.95 / 0.32	21.34 / 0.34	20.68 / 0.33	21.20 / 0.34
7	GLU 96	0.00 / 0.00	0.00 / 0.00	0.00 / 0.00	0.00 / 0.00	0.00 / 0.00	0.00 / 0.00	0.00 / 0.00	0.00 / 0.00	0.00 / 0.00	0.00 / 0.00
8	GLY 97	0.00 / 0.00	0.00 / 0.00	0.00 / 0.00	0.00 / 0.00	0.00 / 0.00	0.00 / 0.00	0.00 / 0.00	0.00 / 0.00	0.00 / 0.00	0.00 / 0.00
9	GLU 98	27.91 / 0.33	0.00 / 0.00	0.00 / 0.00	0.00 / 0.00	0.00 / 0.00	0.00 / 0.00	0.00 / 0.00	0.00 / 0.00	25.74 / 0.31	0.00 / 0.00
10	VAL 99	0.86 / -0.01	15.89 / 0.25	0.00 / 0.00	0.00 / 0.00	0.00 / 0.00	0.00 / 0.00	5.40 / -0.06	0.00 / 0.00	0.00 / 0.00	0.00 / 0.00

position	Interface residue	11 Vcl	12 Vcl	13 Vcl	14 Vcl	15 Vcl	16 Vcl	17 Vcl	18 Vcl	19 Vcl	20 Vcl
-6	LEU 83	0.00 / 0.00	0.00 / 0.00	0.00 / 0.00	11.96 / 0.02	0.00 / 0.00	0.00 / 0.00	26.28 / 0.42	0.00 / 0.00	0.00 / 0.00	25.44 / 0.41
-5	THR 84	0.00 / 0.00	0.00 / 0.00	0.00 / 0.00	0.17 / 0.00	0.00 / 0.00	0.00 / 0.00	18.15 / -0.20 H	0.00 / 0.00	18.41 / 0.29	0.00 / 0.00
-4	ASP 85	0.00 / 0.00	0.00 / 0.00	0.00 / 0.00	0.00 / 0.00	0.00 / 0.00	0.00 / 0.00	0.00 / 0.00	0.00 / 0.00	8.47 / -0.10 H	0.00 / 0.00
-3	**GLU 86**	64.51 / -0.31 HS	64.07 / -0.53 HS	60.87 / -0.39 HS	58.79 / -0.06 HS	54.20 / -0.18 S	58.39 / -0.36 S	57.71 / -0.23 S	63.87 / -0.32 HS	51.56 / -0.13 HS	63.37 / -0.41 S
-2	LEU 87	8.02 / -0.05	8.02 / -0.07	8.02 / -0.07	8.02 / -0.08	8.02 / -0.07	8.02 / -0.06	8.02 / -0.08	8.02 / -0.05	8.02 / -0.06	8.02 / -0.06
-1	**ALA 88**	50.09 / 0.71	44.60 / 0.71	54.78 / 0.71	50.73 / 0.71	49.84 / 0.71	50.01 / 0.71	53.90 / 0.71	53.81 / 0.71	48.65 / 0.71	50.96 / 0.71
0	**PRO 89**	80.48 / 0.94 H	83.54 / 0.93 H	82.11 / 0.94 H	74.07 / 0.83 H	80.39 / 0.93 H	78.75 / 0.90 H	76.23 / 0.87 H	82.41 / 0.90 H	76.88 / 0.89 H	86.60 / 1.00 H
1	PRO 90	23.25 / -0.10 H	24.12 / -0.08	23.22 / -0.08	23.87 / -0.10 H	22.11 / -0.07	24.55 / -0.04 H	24.14 / -0.07 H	25.08 / -0.04 H	23.25 / -0.05 H	21.87 / -0.07
2	**LYS 91**	138.04 / -0.36 HS	125.81 / -0.59 HS	120.02 / -0.70 HS	109.73 / -0.65 HS	126.13 / -0.62 HS	125.48 / -0.66 HS	134.22 / -0.42 HS	125.11 / -0.64 HS	128.70 / -0.63 HS	127.68 / -0.49 HS
3	**PRO 92**	82.15 / 1.19	79.24 / 1.15	80.65 / 1.25	78.48 / 1.20	78.14 / 1.18	77.66 / 1.18	82.51 / 1.19	78.59 / 1.16	79.17 / 1.19	79.56 / 1.20
4	PRO 93	7.21 / -0.07	6.14 / -0.07	5.15 / -0.06	5.88 / -0.07	6.50 / -0.07	5.65 / -0.06	6.50 / -0.07	5.76 / -0.07	6.39 / -0.07	8.47 / -0.10
5	**LEU 94**	93.00 / 1.49	94.50 / 1.51	87.36 / 1.40	86.32 / 1.38	89.01 / 1.42	92.36 / 1.48	91.51 / 1.46	99.00 / 1.58	90.18 / 1.44	93.89 / 1.50
6	PRO 95	22.69 / 0.36	18.35 / 0.29	20.44 / 0.33	19.53 / 0.31	21.79 / 0.35	19.33 / 0.31	21.33 / 0.34	21.47 / 0.34	20.77 / 0.33	19.60 / 0.31
7	GLU 96	0.00 / 0.00	0.00 / 0.00	0.00 / 0.00	0.00 / 0.00	14.60 / -0.17	9.94 / -0.11	0.00 / 0.00	0.00 / 0.00	0.00 / 0.00	1.23 / -0.01
8	GLY 97	0.00 / 0.00	0.00 / 0.00	0.00 / 0.00	0.00 / 0.00	0.00 / 0.00	0.00 / 0.00	0.00 / 0.00	0.00 / 0.00	0.00 / 0.00	0.00 / 0.00
9	GLU 98	0.00 / 0.00	11.32 / 0.18	0.00 / 0.00	0.00 / 0.00	0.00 / 0.00	0.00 / 0.00	8.26 / 0.05	37.88 / 0.04	0.00 / 0.00	0.00 / 0.00
10	VAL 99	0.00 / 0.00	51.55 / -0.24	0.00 / 0.00	0.00 / 0.00	0.00 / 0.00	0.00 / 0.00	0.00 / 0.00	0.00 / 0.00	0.00 / 0.00	0.00 / 0.00

position	Interface residue	21 Vcl	22 Vcl	23 Vcl	24 Vcl	25 Vcl	26 Vcl	27 Vcl	28 Vcl	29 Vcl	30 Vcl
-6	LEU 83	0.00 / 0.00	0.00 / 0.00	3.56 / -0.04	0.00 / 0.00	0.00 / 0.00	0.00 / 0.00	0.00 / 0.00	0.00 / 0.00	4.46 / -0.00	42.67 / 0.28
-5	THR 84	2.46 / -0.03	0.67 / 0.01	18.92 / 0.30	0.00 / 0.00	0.00 / 0.00	0.00 / 0.00	0.00 / 0.00	26.76 / 0.43	32.54 / 0.14 H	4.40 / 0.06
-4	ASP 85	0.00 / 0.00	0.00 / 0.00	7.49 / -0.09	0.00 / 0.00	0.00 / 0.00	0.00 / 0.00	0.00 / 0.00	12.53 / -0.14	9.45 / -0.11 H	0.00 / 0.00
-3	**GLU 86**	81.99 / -0.49 HS	65.99 / -0.33 HS	62.69 / -0.49 HS	52.67 / -0.45 S	52.67 / -0.45 S	52.67 / -0.45 S	65.21 / -0.50 HS	63.90 / -0.31 HS	45.03 / -0.09 H	66.63 / -0.33 HS
-2	LEU 87	8.02 / -0.05	8.02 / -0.06	8.02 / -0.05	8.02 / -0.06	8.02 / -0.06	8.02 / -0.06	8.02 / -0.06	8.02 / -0.02	8.02 / -0.02	8.02 / -0.02
-1	**ALA 88**	55.27 / 0.71	52.66 / 0.71	48.71 / 0.71	49.33 / 0.71	49.33 / 0.71	49.33 / 0.71	51.94 / 0.71	50.64 / 0.71	49.28 / 0.71	51.82 / 0.71
0	**PRO 89**	80.12 / 0.92 H	83.47 / 0.96 H	74.12 / 0.82 H	81.03 / 0.95 H	81.03 / 0.95 H	81.03 / 0.95 H	77.21 / 0.87 H	81.90 / 0.96 H	77.16 / 0.87 H	85.89 / 1.00 H
1	PRO 90	19.88 / -0.11	24.85 / -0.05 H	25.08 / -0.06 H	21.20 / -0.09	21.20 / -0.09	21.20 / -0.09	19.36 / -0.08 H	21.96 / -0.07	23.91 / -0.06 H	20.66 / -0.04 H
2	**LYS 91**	127.37 / -0.59 HS	132.06 / -0.45 HS	127.64 / -0.60 HS	126.81 / -0.60 HS	126.81 / -0.60 HS	126.81 / -0.60 HS	119.65 / -0.74 HS	125.36 / -0.62 HS	130.33 / -0.55 HS	130.75 / -0.56 HS
3	**PRO 92**	79.77 / 1.17	83.90 / 1.21	76.70 / 1.15	80.37 / 1.20	80.37 / 1.20	80.37 / 1.20	78.21 / 1.18	79.47 / 1.17	77.20 / 1.15	73.11 / 1.06
4	PRO 93	5.88 / -0.07	7.15 / -0.08	5.64 / -0.06	6.57 / -0.04	6.57 / -0.04	6.57 / -0.04	6.12 / -0.07	7.13 / -0.05	6.02 / -0.07	5.15 / -0.06
5	**LEU 94**	97.19 / 1.55	95.20 / 1.52	90.50 / 1.45	87.82 / 1.41	87.82 / 1.41	87.82 / 1.41	87.17 / 1.39	92.00 / 1.47	90.86 / 1.45	89.17 / 1.43
6	PRO 95	20.54 / 0.33	21.36 / 0.34	20.28 / 0.32	22.21 / 0.36	22.21 / 0.36	22.21 / 0.36	18.52 / 0.30	22.63 / 0.36	20.13 / 0.32	21.70 / 0.35
7	GLU 96	0.00 / 0.00	0.00 / 0.00	0.00 / 0.00	0.00 / 0.00	0.00 / 0.00	0.00 / 0.00	0.00 / 0.00	0.00 / 0.00	0.00 / 0.00	0.00 / 0.00
8	GLY 97	0.00 / 0.00	0.00 / 0.00	0.00 / 0.00	0.00 / 0.00	0.00 / 0.00	0.00 / 0.00	0.00 / 0.00	0.00 / 0.00	0.00 / 0.00	0.00 / 0.00
9	GLU 98	0.12 / -0.00	33.14 / 0.06	0.00 / 0.00	0.00 / 0.00	0.00 / 0.00	0.00 / 0.00	0.00 / 0.00	0.00 / 0.00	0.00 / 0.00	0.00 / 0.00
10	VAL 99	0.00 / 0.00	0.00 / 0.00	0.00 / 0.00	0.00 / 0.00	0.00 / 0.00	0.00 / 0.00	21.76 / 0.35	0.00 / 0.00	0.00 / 0.00	0.00 / 0.00

position	Interface residue	31 Vcl	32 Vcl	33 Vcl	34 Vcl	35 Vcl	36 Vcl	37 Vcl	38 Vcl	39 Vcl	40 Vcl
-6	LEU 83	5.94 / -0.04	0.00 / 0.00	14.32 / -0.16	0.00 / 0.00	0.00 / 0.00	8.53 / 0.14	64.78 / 0.42 H	7.29 / -0.02	4.66 / -0.05	0.00 / 0.00
-5	THR 84	0.00 / 0.00	0.00 / 0.00	3.18 / 0.05	0.00 / 0.00	0.00 / 0.00	1.60 / -0.02	35.11 / -0.14 H	76.17 / 0.90 H	0.00 / 0.00	0.00 / 0.00
-4	ASP 85	0.00 / 0.00	0.00 / 0.00	0.12 / -0.00	0.00 / 0.00	0.00 / 0.00	0.00 / 0.00	5.65 / -0.06 H	0.14 / -0.00	0.00 / 0.00	0.00 / 0.00
-3	**GLU 86**	67.91 / -0.35 HS	42.03 / -0.16 HS	56.29 / -0.38 S	53.03 / 0.06 HS	65.35 / -0.17 HS	65.25 / -0.35 S	52.00 / -0.16 HS	42.12 / 0.05	50.22 / -0.33 HS	60.45 / -0.00 S
-2	LEU 87	8.02 / -0.04	8.02 / -0.08	8.02 / -0.05	8.02 / -0.05	8.02 / -0.07	8.02 / -0.08	8.02 / -0.08	8.02 / -0.03	8.02 / -0.08	8.02 / -0.09
-1	**ALA 88**	57.60 / 0.71	48.09 / 0.71	52.29 / 0.71	48.52 / 0.71	51.48 / 0.71	48.11 / 0.71	46.74 / 0.71	50.49 / 0.71	47.98 / 0.71	50.55 / 0.71
0	**PRO 89**	80.63 / 0.89 H	81.39 / 0.94 H	85.03 / 0.98 H	80.28 / 0.93 H	82.45 / 0.97 H	81.48 / 0.97 H	77.69 / 0.89 H	78.86 / 0.91 H	82.70 / 0.96 H	75.31 / 0.85 H
1	PRO 90	24.34 / -0.07 H	21.42 / -0.12	22.68 / -0.07	26.07 / -0.03	17.00 / -0.08	18.78 / -0.09	24.25 / -0.05 H	22.18 / -0.06	16.92 / -0.08	22.22 / -0.08 H
2	**LYS 91**	127.07 / -0.59 HS	131.04 / -0.53 HS	125.92 / -0.60 HS	128.54 / -0.60 HS	134.20 / -0.48 HS	135.20 / -0.40 HS	137.71 / -0.36 HS	123.18 / -0.65 HS	133.98 / -0.44 HS	126.87 / -0.62 HS
3	**PRO 92**	79.93 / 1.18	81.36 / 1.19	80.16 / 1.16	81.02 / 1.20	79.12 / 1.11	85.78 / 1.26	87.96 / 1.28	77.63 / 1.15	82.94 / 1.22	79.53 / 1.21
4	PRO 93	6.50 / -0.07	6.26 / -0.07	6.38 / -0.07	6.68 / -0.07	5.28 / -0.06	8.33 / -0.07	17.32 / 0.07	4.54 / -0.05	9.61 / -0.07	5.77 / -0.07
5	**LEU 94**	93.18 / 1.49	92.67 / 1.48	88.04 / 1.41	92.35 / 1.48	84.68 / 1.35	93.36 / 1.49	88.87 / 1.42	92.37 / 1.48	83.85 / 1.34	86.84 / 1.39
6	PRO 95	21.20 / 0.34	20.03 / 0.32	20.70 / 0.33	20.70 / 0.33	22.38 / 0.36	20.68 / 0.33	21.01 / 0.34	18.88 / 0.30	22.20 / 0.36	21.59 / 0.35
7	GLU 96	0.00 / 0.00	0.00 / 0.00	0.00 / 0.00	0.00 / 0.00	0.00 / 0.00	0.00 / 0.00	0.00 / 0.00	0.86 / -0.01	0.00 / 0.00	0.00 / 0.00
8	GLY 97	0.00 / 0.00	0.00 / 0.00	0.00 / 0.00	0.00 / 0.00	0.00 / 0.00	0.00 / 0.00	0.00 / 0.00	0.00 / 0.00	0.00 / 0.00	0.00 / 0.00
9	GLU 98	0.00 / 0.00	18.27 / 0.26	0.00 / 0.00	0.00 / 0.00	0.00 / 0.00	0.00 / 0.00	0.00 / 0.00	11.15 / 0.18	0.00 / 0.00	0.00 / 0.00
10	VAL 99	0.00 / 0.00	0.00 / 0.00	0.00 / 0.00	33.64 / 0.46	0.00 / 0.00	0.00 / 0.00	0.00 / 0.00	0.00 / 0.00	0.00 / 0.00	19.90 / 0.32

	Vinculin			
SDE	-6	854	L	S
	-5	855	T	
	-4	856	D	
	-3	857	E	HS
	-2	858	L	
core	-1	859	**A**	
	0	860	**P**	H
	1	861	P	H
	2	862	**K**	HS
	3	863	**P**	
	4	864	P	
	5	865	**L**	
	6	866	P	
SDE	7	867	E	
	8	868	G	
	9	869	E	
	10	870	V	

| per 10% of BSA
-
|- ||
||| - ||||
||||| - ||||||
|||||||-|||||||||

BSA = burried of accessible surface area

Supplementary table S6. Interface analysis of CAS SH3-Vinculin (ligand part).
Hydrogen bonds and nonpolar contacts between Cas SH3 and its ligands were analyzed with PISA with default settings. Prior to analysis, the linker sequence (residues 75-82) was removed from the chimeric proteins. Solvent accessible residues are colored yellow and inaccessible residues are blue. ΔiG represents free energy of dissociation (solvation energy effect, kcal/mol). BSA means Buried Surface Area (Å²) and is displayed with bars, one bar per 10%. HSDC indicates residues that contain the across-interface hydrogen bond, salt bridge, or disulfide bond atoms. The corresponding table cells have a brown background and contain letter H for a hydrogen bond, S for a salt bridge, and HS for a combination of hydrogen bond and salt bridge.
http://www.ebi.ac.uk/msd-srv/prot_int/pistart.html

##	UNIPROT	CAS SH3	Vinculin		1	2	3	4	5	6	7	8	9	10	11	12	13	14	15	16	17	18	19	20	21	22	23	24	25	26	27	28	29	30	31	32	33	34	35	36	37	38	39	40	SDE	Pocket S1	Pocket S2	Pocket S3	SDE (ext. S3)
**Hydrogen bonds** (interface) XML					Structure (number)																																												
1	Arg25	A:ARG 27[NH1]	B:LEU 83[O]	-6																																					X				Arg25				
2	Lys26	A:LYS 28[NZ]	B:THR 84[OG1]	-5																	X																								Lys26				
3	Arg25	A:ARG 27[NH1]	B:THR 84[OG1]	-5																																	X	X			Arg25				
4		A:ARG 27[NH2]	B:THR 84[OG1]	-5																																		X							
5	Lys26	A:LYS 28[NZ]	B:THR 84[O]	-5																													X								X				Lys26				
6	Lys26	A:LYS 28[NZ]	B:ASP 85[O]	-4																			X										X								X				Lys26				
7	Lys69	A:LYS 71[NZ]	B:GLU 86[OE2]	-3																					X																				Lys69				
8	Lys26	A:LYS 28[NZ]	B:GLU 86[O]	-3																			X																						Lys26				
9	Tyr12	A:TYR 14[HH]	B:GLU 86[OE2]	-3																					X		X						X												Tyr12	Tyr12			
10	Lys26	A:LYS 28[NZ]	B:GLU 86[OE2]	-3		X	X					X		X	X	X	X									X	X				X	X		X	X			X	X		X		X		Lys26				
11		A:LYS 28[NZ]	B:GLU 86[OE1]	-3				X	X	X	X		X	X	X			X				X	X		X	X					X				X	X			X										
12	Arg59	A:ARG 61[HE]	B:PRO 89[O]	0	X	X	X	X	X	X	X	X	X	X	X	X	X	X	X	X	X	X	X	X	X	X	X	X	X	X	X	X	X	X	X	X	X	X	X	X	X	X	X	X		Arg59	Arg59		
13	Arg59	A:ARG 61[NH2]	B:PRO 89[O]	0	X	X	X	X	X	X	X	X	X	X	X	X	X	X	X	X	X	X	X	X	X	X	X	X	X	X	X	X	X	X	X	X	X	X	X	X		Arg59	Arg59		
14	Asn58	A:ASN 60[ND2]	B:PRO 90[O]	1	X	X	X	X	X	X	X	X		X	X			X		X	X	X	X			X	X				X		X	X	X						X			X		Asn58	Asn58		
15	Val15	A:VAL 17[O]	B:LYS 91[NZ]	2	X	X	X	X	X	X	X	X	X	X	X	X	X		X	X	X	X	X	X	X	X	X	X	X	X	X	X	X	X	X	X	X	X	X	X	X	X	X	X			Val15		
16	Glu21	A:GLU 23[OE1]	B:LYS 91[NZ]	2	X	X	X	X	X	X	X	X	X	X	X	X	X	X	X	X	X	X	X	X	X	X	X	X	X	X	X	X	X	X	X	X	X	X	X	X	X		X			Glu21	Glu21	
17		A:GLU 23[OE2]	B:LYS 91[NZ]	2	X	X	X	X	X	X	X	X	X	X	X		X	X	X	X	X	X	X		X		X	X	X	X	X	X	X	X	X	X	X	X	X	X	X	X	X	X					
18	Glu17	A:GLU 19[OE1]	B:LYS 91[NZ]	2								X	X				X			X	X		X	X		X	X	X	X	X	X		X	X		X				X	X	X		X			Glu17	Glu17	
19		A:GLU 19[OE2]	B:LYS 91[NZ]	2	X	X	X	X	X	X	X				X	X		X	X			X			X							X			X		X	X	X				X						

##	UNIPROT	CAS SH3	Vinculin		1	2	3	4	5	6	7	8	9	10	11	12	13	14	15	16	17	18	19	20	21	22	23	24	25	26	27	28	29	30	31	32	33	34	35	36	37	38	39	40	SDE	Pocket S1	Pocket S2	Pocket S3	SDE (ext. S3)
**Salt bridges** (Interface) XML					Structure (number)																																												
1	Lys26	A:LYS 28[NZ]	B:GLU 86[OE1]	-3	X	X	X	X	X	X	X		X	X	X	X	X	X	X	X	X	X	X	X	X	X	X	X	X	X	X			X	X	X	X		X	X				X	Lys26				
2		A:LYS 28[NZ]	B:GLU 86[OE2]	-3	X	X	X		X			X		X	X	X	X		X	X	X	X		X	X	X	X	X	X	X	X	X		X	X	X	X	X	X	X	X		X	X					
3	Glu21	A:GLU 23[OE1]	B:LYS 91[NZ]	2	X	X	X	X	X	X	X	X	X	X	X	X	X	X	X	X	X	X	X	X	X	X	X	X	X	X	X	X	X	X	X	X	X	X	X	X	X	X	X	X			Glu21	Glu21	
4		A:GLU 23[OE2]	B:LYS 91[NZ]	2	X	X	X	X	X	X	X	X	X	X	X	X	X	X	X	X	X	X	X	X	X	X	X	X	X	X	X	X	X	X	X	X	X	X	X	X	X	X	X	X					
5	Glu17	A:GLU 19[OE1]	B:LYS 91[NZ]	2					X			X	X	X			X			X	X		X	X		X	X	X	X	X	X		X	X		X				X	X	X		X			Glu17	Glu17	
6		A:GLU 19[OE2]	B:LYS 91[NZ]	2	X	X	X	X		X	X			X	X	X		X	X			X			X		X					X			X		X	X	X		X		X						

number of structures
0-5 %
6-20 %
21-50 %
51-80%
81-100%

SH3 numbering from UNIPROT: X-2

	Vinculin			UNIPROT
	-6	L	83	854
	-5	T	84	855
SDE	-4	D	85	856
	-3	E	86	857
	-2	L	87	858
	-1	**A**	88	859
	0	**P**	89	860
	1	P	90	861
core	2	**K**	91	862
	3	**P**	92	863
	4	P	93	864
	5	**L**	94	865
	6	P	95	866
	7	E	96	867
SDE	8	G	97	868
	9	E	98	869
	10	V	99	870

Supplementary table S7. Interface analysis of CAS SH3-Vinculin (hydrogen bonds).

Hydrogen bonds and salt bridges between Cas SH3 and its ligands were analyzed with PISA with default settings. Prior to analysis, the linker sequence (residues 75-82) was removed from the chimeric proteins. The atoms that form hydrogen bonds and salt bridges are indicated by crosses.

CAS_SH3-ligand_variant	Int.ene	Disp	H-bond
CAS-PTP_PEST-A12	-115.3	-55.8	-24.5
CAS-PTP_PEST-E12	-114.5	-58.3	-25.2
CAS-PTP_PEST-F12	-110.7	-60.7	-24.0
CAS-PTP_PEST-Y12	-124.6	-61.9	-30.8
CAS-PTP_PEST-pY12	-103.2	-57.8	-25.9

CAS_SH3-ligand_variant	Int.ene	Disp	H-bond
CAS-Vinculin-A12	-89.4	-56.5	-19.0
CAS-Vinculin-E12	-93.1	-61.8	-19.6
CAS-Vinculin-F12	-102.8	-63.5	-19.0
CAS-Vinculin-Y12	-104.1	-63.5	-19.1
CAS-Vinculin-pY12	-83.7	-57.5	-18.5

Supplementary table S8. Interaction energies – Quantum mechanics.
Interaction energies (Int. ene) and their dispersion (Disp) and hydrogen-bonding (H-bond) components of CAS vs. PTP-PEST/Vinculin binding for the wild-type, Tyr12 phosphorylated, and Tyr12 mutated CAS variants. All values are in kcal/mol.

Uncropped immunoblots for Figure 7:

C

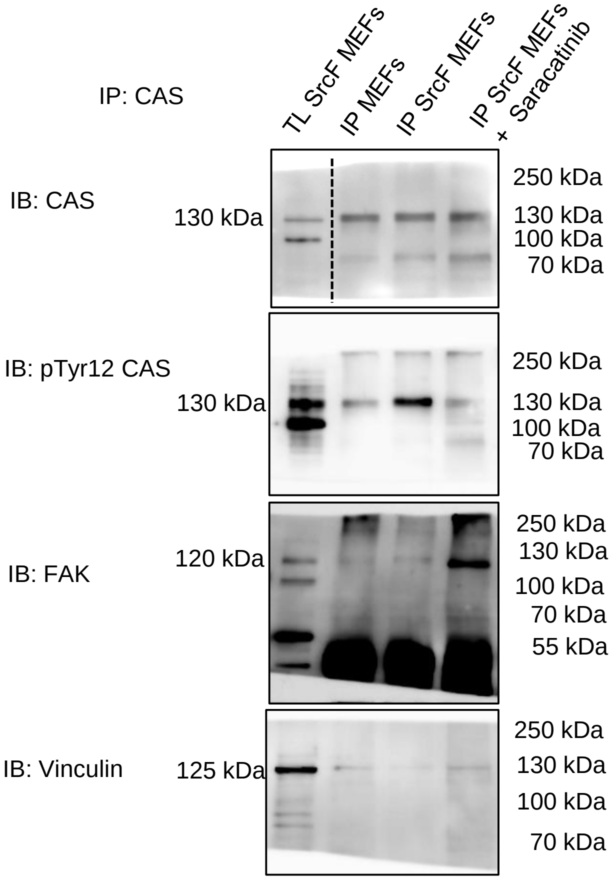

D

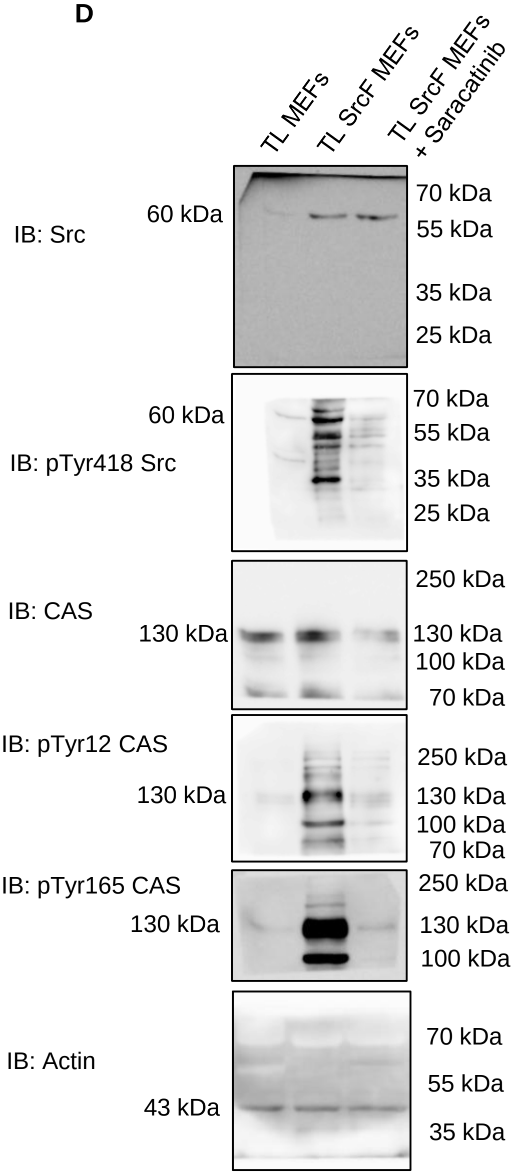

Uncropped immunoblots for Figure 8:

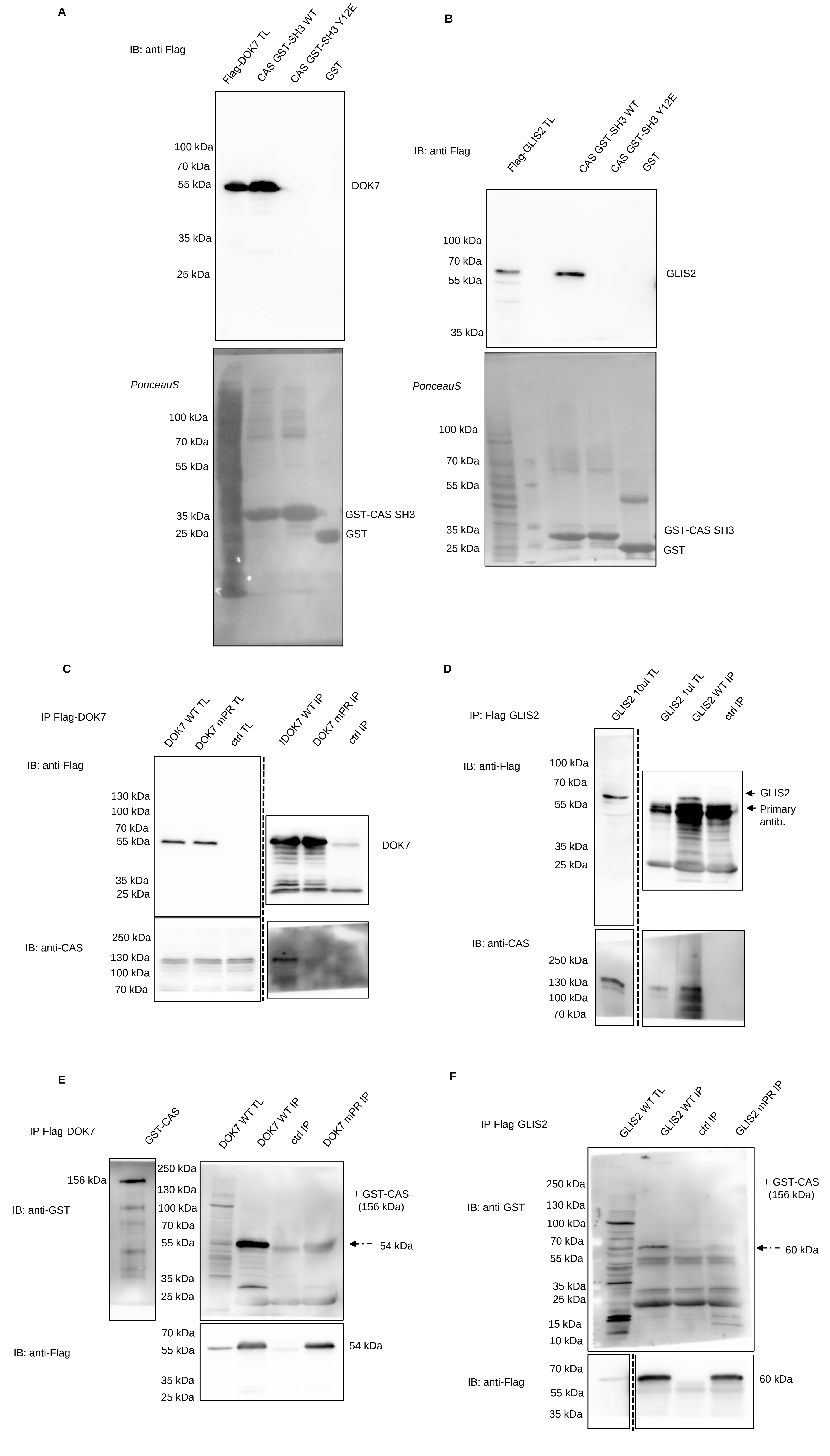

Supplement: Supplementary file 1 — Supplementary figures, tables and blots [file 41598_2017_8303_MOESM1_ESM.pdf]
